# Coordinating Environmental Genomics and Geochemistry Reveals Metabolic Transitions in a Hot Spring Ecosystem

**DOI:** 10.1371/journal.pone.0038108

**Published:** 2012-06-04

**Authors:** Wesley D. Swingley, D’Arcy R. Meyer-Dombard, Everett L. Shock, Eric B. Alsop, Heinz D. Falenski, Jeff R. Havig, Jason Raymond

**Affiliations:** 1 School of Natural Sciences, University of California Merced, Merced, California, United States of America; 2 Department of Earth and Environmental Sciences, University of Illinois at Chicago, Chicago, Illinois, United States of America; 3 School of Earth and Space Exploration, Arizona State University, Tempe, Arizona, United States of America; 4 Department of Chemistry and Biochemistry, Arizona State University, Tempe, Arizona, United States of America; Argonne National Laboratory, United States of America

## Abstract

We have constructed a conceptual model of biogeochemical cycles and metabolic and microbial community shifts within a hot spring ecosystem via coordinated analysis of the “Bison Pool” (BP) Environmental Genome and a complementary contextual geochemical dataset of ∼75 geochemical parameters. 2,321 16S rRNA clones and 470 megabases of environmental sequence data were produced from biofilms at five sites along the outflow of BP, an alkaline hot spring in Sentinel Meadow (Lower Geyser Basin) of Yellowstone National Park. This channel acts as a >22 m gradient of decreasing temperature, increasing dissolved oxygen, and changing availability of biologically important chemical species, such as those containing nitrogen and sulfur. Microbial life at BP transitions from a 92°C chemotrophic streamer biofilm community in the BP source pool to a 56°C phototrophic mat community. We improved automated annotation of the BP environmental genomes using BLAST-based Markov clustering. We have also assigned environmental genome sequences to individual microbial community members by complementing traditional homology-based assignment with nucleotide word-usage algorithms, allowing more than 70% of all reads to be assigned to source organisms. This assignment yields high genome coverage in dominant community members, facilitating reconstruction of nearly complete metabolic profiles and in-depth analysis of the relation between geochemical and metabolic changes along the outflow. We show that changes in environmental conditions and energy availability are associated with dramatic shifts in microbial communities and metabolic function. We have also identified an organism constituting a novel phylum in a metabolic “transition” community, located physically between the chemotroph- and phototroph-dominated sites. The complementary analysis of biogeochemical and environmental genomic data from BP has allowed us to build ecosystem-based conceptual models for this hot spring, reconstructing whole metabolic networks in order to illuminate community roles in shaping and responding to geochemical variability.

## Introduction

The continuous microbial community along the outflow of “Bison Pool” (BP), Yellowstone National Park (YNP), is an ideal system for understanding how the ecological stress of a temperature gradient, and the corresponding change in pH and geochemistry, drives microbial community development. BP, a slightly alkaline (pH 7.3–8.3) spring in the Sentinel Meadows basin, has been the target of recent interest linking geochemistry to microbial community structure, the formation of streamer biofilm communities in the chemosynthetic zones (>68°C), isotopic composition of biofilms, and protein inventories [1], [2], [3], [4]. This steady-flow, boiling spring features a silica-encrusted source pool with a single run-off channel that supports a gradient of microbial communities as the boiling source-water cools 40°C in just over 20 m. The physicochemical features of BP provide a test bed for hypotheses related to changes in microbial communities, metabolic capacity, and biogeochemical cycling as a function of downstream flow. Our guiding hypotheses were: 1) observations of shifts in microbial communities should mirror shifts in the dominance of metabolic “lifestyles”, with distance down the outflow channel. 2) community and genetic capacity for facilitating biogeochemical cycling should shift as a function of environmental conditions at different points in the outflow, and 3) while unique environments exist in the outflow channel, we will find evidence for both specialists and generalists in this thermophilic ecosystem. These relationships are explored here, using a metagenomic dataset collected at consecutive points down the BP outflow channel.

Metagenomic data have been reported from single communities, featuring as few as one dominant species [5], [6], [7], and from multiple geographically-distinct communities [8], [9], [10], [11], [12]. In communities with relatively few dominant species, the separation and assembly of species-specific sequences yielded complete or nearly complete genome assemblies [5], [6], [7]. Conversely, the complex, oligotrophic environments sampled during the *Sorcerer II* Global Ocean Sampling Expedition yielded few large assemblies, despite the addition of a long paired-end fosmid library designed to facilitate assembly [11], [12]. Here we present environmental genome sequence from biofilms at five contiguous sites along the source and outflow channel of BP-hereafter referred to as the “Bison Pool” Environmental Genome (BPEG). This is the first report of metagenome data of multiple, geochemically- and physically-related samples from a hydrothermal system.

Only within the past decade has whole-community DNA sequencing become feasible [5], [6], [7], [8], [9], [10], [11], [12], [13], [14], often necessitating the development of novel analysis packages in order to analyze these enormous datasets [15], [16], [17], [18], [19], [20], [21], [22], [23]. One of the key steps in dissecting the morass of data within a metagenome is the assignment of individual sequencing reads/scaffolds/contigs to taxonomic bins [24], [25]. This process, called “binning,” not only reduces the volume of information, but allows the reconstruction of metabolic models for single organisms in a complex environmental community. Traditional binning methods have relied on homology-based searches such as BLAST [26] in order to reliably match sequence reads to a reference sequence database [15], [16]; however, this method relies on comparison to whole-genome sequences from closely related organisms, which are often not yet available for hydrothermal ecosystems. One way to avoid this database bias is to assign metagenome reads using the unique nucleotide substitution bias found in all organisms [19], [27]. While GC content (a single-nucleotide “word”) is a common metric for distinguishing organisms, the genetic bias toward 2, 3, 4, and longer nucleotide “words” can provide even greater resolution in the differentiation between community members [17], [18], [19], [20], [21], [22], [28]. Using tetranucleotide (4-nucleotide word) frequency clustering to reinforce homology-based binning, we were able to bin a majority of BPEG reads allowing nearly complete genomic and metabolic reconstruction of key community members.

Previous work at BP made use of both geochemical and genetic datasets to identify shifts in community diversity in biofilms, demonstrate a novel ecotone between metabolic zones in the outflow channel, and reveal evidence for biological carbon and nitrogen cycling [2], [3], [4]. In addition, predictions of protein stability at BP locations were linked to BPEG data [1]. Together, these previous studies provide the background for our hypotheses, stated above. In this report, we present three primary results. We demonstrate that tetranucleotide-binning can confidently assign metagenome reads to their source phylum-a useful tool when reference genomes are lacking. We assign metabolic capacity to individual consensus genomes at each sample location. And, lastly, we coordinate geochemical and genetic evidence for biogeochemical cycling. Our analysis shows a continuum of microbial communities along a temperature and geochemical gradient, shifting both community structure and metabolic potential in response to changing environmental conditions. Further, we are able to identify microbial specialists vs. generalists in this system, and observe variation in the genomic potential for sulfur and nitrogen biogeochemical cycling.

## Methods

### Sample Collection

**Figure 1:**
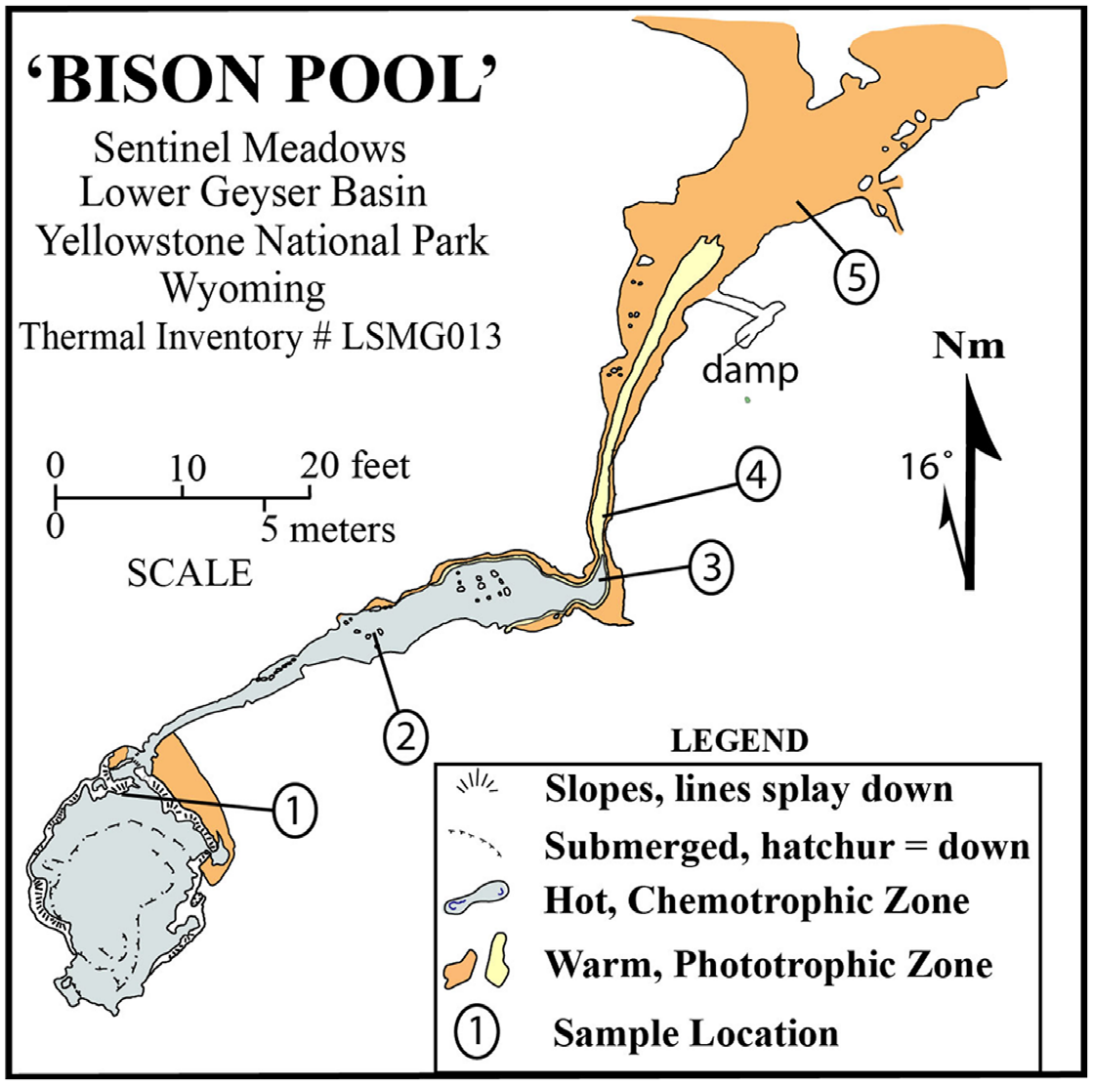
Map of BP, showing the five sample locations used in this study. Modified from Havig et al. (2011) and originally drafted by G.R. Osburn.

Samples of biofilm were collected on July 19, 2005 at “Bison Pool” (Thermal Inventory Number LSMG 013) with sterile implements from the main pool and four locations along the BP outflow, and named as sites 1–5, from source to bottom of the outflow. A map of BP at the time of sampling is shown in Figure 1, and images are found in Figure S1. Additional analyses and description of the BP location can be found in Meyer-Dombard et al. [4], Havig et al. [2] and Dick & Shock [1]. The five samples analyzed here consist of native Streamer Biofilm Communities (e.g. SBCs, [4]) at sites 1–3, and stratified photosynthetic mats at sites 4 and 5. Detailed photographs of each of the five collected biofilms are given in Havig et al. [2], and a few are available in Figure S1b, and S1c. SBC samples, which typically lay <1 cm below the water-air interface, were collected with forceps. Photosynthetic mats were sampled vertically from the surface of the mat down to the sediment interface, avoiding sediment particles as possible, and homogenized (individual layers of the mat were not separately analyzed). Photosynthetic mats were typically <0.5 cm thick, and the top most layers were a few mm below the water-air interface. Samples were placed in sterile vials on ice for several hours while in the field, and were frozen immediately on return to our field laboratory. Samples for geochemical analysis of spring fluids were specifically collected at each of the above sites 1–5 to coordinate with the samples destined for genomic analysis. Fluids were collected in the flowing portion of the runoff channel above the sediment interface (i.e. not pore fluids). Samples for analysis of trace elements, major ions, and redox sensitive species were collected, in accordance with previous methods [29], [30]. Measurements made in the field were done in replicate when possible, and we have previously estimated an uncertainty of ∼10%; IC and ICP-MS analyses are done in replicate and typical uncertainties are <1% [28]. Sample collection for field studies was carried out under National Park Service permit YELL-05434.

### Energy Estimates

Chemical affinities (A_r_) were estimated for 287 separate reactions, representing plausible sources of energy for chemolithotrophic metabolism, using the pH measurements at each of the 5 locations and the slopes and intercepts reported in Table 5 of Shock et al. 2010 [30]. “Bison Pool” was among the springs evaluated in the Shock et al. 2010 [30] dataset, so this method simply estimates the energy available via these reactions at BP in 2005. The 287 reactions are given in full in Table S3, but are also listed in Shock et al. 2010 [30].

### DNA Extraction

Environmental DNA was extracted from the biofilm samples using the CTAB method as proscribed by the Joint Genome Institute (JGI) (http://my.jgi.doe.gov/general/) and described in Meyer-Dombard et al. [3]. DNA was shipped to JGI as part of the 2005 Microbial Sequencing Program (DOE) for library construction, Sanger sequencing, assembly (of each site individually), and automated annotation (http://www.jgi.doe.gov/sequencing/protocols/prots_production.html). This included a DNA quality control step that produced 16S rRNA clone libraries for each location. 16S rRNA sequences, raw Sanger sequencing reads, contiguous assemblies (contigs), scaffolds (linked contigs), and protein predictions provided by JGI were used through the entirety of this analysis. Sequence data can be found on NCBI under project id PRJNA71319.

### 16S rRNA Analysis

Taxonomic identification of clone library 16S rRNA (from JGI’s quality control) as well as metagenome 16S rRNA sequences was performed using the Ribosome Database Project’s (RDP) Classifier tool (http://rdp.cme.msu.edu) [31]. Metagenome 16S rRNA sequences were located within the read library by BLAST (Basic Local Alignment Search Tool) homology to a number of bacterial and archaeal reference 16S rRNA sequences (*Escherichia coli*, *Staphylococcus aureus*, *Pyrobaculum aerophilum*, and *Halobacterium salinarum*; www.ncbi.nlm.nih.gov). Assignments below RDP classifier’s suggested cutoff (S_ab of 80% for full-length 16S rRNA and 50% for 16S rRNA fragments) were labeled as “unassigned”.

Sequences were aligned with ClustalW2 [32] and alignments were filtered using a Shannon information entropy cutoff of 2.0 and deletion of positions with >50% gaps or 100% identity. Resulting alignments were separated into Operational Taxonomic Units (OTUs) using furthest neighbor assignment based on methods introduced in DOTUR [33] at 97% similarity identity cutoffs. Reference sequences for phylogenetic reconstructions were taken from RDP and the National Center for Biotechnology Information (NCBI) (http://www.ncbi.nlm.nih.gov). Trees were constructed using maximum likelihood methodology with the program PHYLIP [34] with random input order and 10 replicates. Diversity measurements were calculated for 16S rRNA assignments using published methods by Shannon [35], Simpson [36], and Pielou [37].

### Homology Binning of BPEG Nucleotides

Two methods were employed in homology binning. 1) BP metagenome scaffolds and singleton reads (those unassigned to any contigs or scaffolds) were matched versus NCBI’s non-redundant protein database (*nr*) using BLAST’s blastx program, a translated protein vs. protein analysis. These data were imported into Megan [16], a last common ancestor (LCA) taxonomic identification tool. 2) BP sequences were also matched against all NCBI complete and draft microbial reference genomes (as of April 2011) using BLAST’s blastn program, a nucleotide vs. nucleotide analysis.

Final homology binning was based on a combination of Megan’s inclusive LCA assignments (Min Support of 5; Min Score of 35; Top Percent 10) and conservative NCBI blastn assignments (only matches with a bit-score above 300 were assigned to a bin). Sequences were assigned to a bin in the case of perfect agreement between both methods, stated above, at each major taxonomic level (superkingdom [domain], phylum, class, order, family, and genus). Assignments were also confirmed under three additional conditions: 1) Megan assigned the read at the species level; 2) blastn assignment carried a bit-score higher than 1000; and 3) the Megan and blastn assignment matched at two taxonomic levels deeper (more toward superkingdom) than the best Megan assignment, in which case the assignment closer to species-level was considered confident (in order to assign cases where, for example, class-level assignment matched using both methods, but one of the two methods was below the threshold for family-level assignment).

### Tetranucleotide Binning of BPEG Nucleotides

In order to assign BPEG sequences to their originating microbial taxon, BP metagenome scaffold and singleton-read tetranucleotide frequencies (that is, the frequencies of the 256 unique 4-nucleotide words) were calculated using J. Craig Venter Institute’s Upload Utility for Scatter Plot Viewer (http://gos.jcvi.org/openAccess/kmerAnalysisUploadForm.html), a principal components analysis (PCA) tool for metagenome reads. Homology bins (described above) were used as guidelines for the assignments of tetranucleotide principal components. We constructed a pair-wise matrix of the Pearson Correlation between the first ten PCA dimensions for all sequences within each site. Sequences that were over 900 nucleotides in length but unassigned using the above (Megan and blastn) methods were assigned to a taxonomic bin if their average correlation to all members of a homology-based taxonomic bin was greater than the average correlation between all sequences within the bin (internal read-to-read correlations) and no greater than that average for any other homology-based bins (in cases where a sequence matches more than one bin). In cases where a single paired-read was assigned to a particular bin and its mate was unassigned, both reads were assigned to that bin. While our conservative read assignment drastically limited the total number of assigned sequences (though this was increased by co-assigning mate pairs), it also maximized the probability that sequences were assigned to the correct bin. Diversity measurements were calculated for phylum-level bin assignments using published methods by Shannon [35], Simpson [36], and Pielou [37].

### Markov Clustering

BPEG predicted proteins were combined with all protein sequences from the Kyoto Encyclopedia of Genes and Genomes (KEGG) [38] that have Enzyme Commission (EC) numbers. An all-versus-all BLAST of this combined data set to itself was used to seed MCL [39], a Markov clustering program used to construct a database of protein families. MCL was used with an inflation parameter of 1.2 and an e-value cutoff of 10^−40^ to limit the separation of single EC numbers into multiple clusters and single clusters containing multiple EC numbers. BPEG proteins were then assigned EC numbers based on KEGG proteins within the clusters. Owing to the presence of synonymous and overlapping EC numbers, multiple EC numbers in a single cluster were assigned to BPEG proteins if they composed more than 1% of all EC numbers in that cluster.

### Metabolic Networks

By applying the KEGG metabolic pathway maps [38] to our Markov protein families (above), we calculated the estimated coverage (percentage of genome size that has been sequenced) within each taxonomic bin and sample site. Small homology bins containing less than 0.2% of the total BPEG sequences were omitted from these calculations. For full pathway analysis, all EC numbers assigned to BPEG proteins were treated as binary presence/absence, meaning only a single copy of a given enzyme need be present in any sample dataset. In order to limit the bias caused by differences in the relative proportion of single community members, binary EC values within each sample were normalized against the average duplication (presence of multiple copies of any EC) given as the total raw number of EC-containing proteins (non-binary) divided by the total of EC families. An EC was scored as present if the normalized presence/absence value was above 0.25, an arbitrary cutoff that affected the least change on all but those bins with >4-fold coverage of their protein library.

## Results and Discussion

### I. The Physical and Chemical Environment of “Bison Pool”

In any hydrothermal setting, geochemical reactions are driven by physical processes, such as cooling, evaporation, or mixing with other fluids or the atmosphere. As fluids cool, reaction rates slow, resulting in oxidation/reduction disequilibria, which provide the chemical energy sources tapped by chemotrophs [30]. Likewise, the temperature of a given hot spring is in part driven by surges of hydrothermal fluids and gas. The consequences of geochemical changes can be assessed by analyzing individual processes within the context of the geologic environment from which measurements were made.

The outflow channel of BP stretches ∼22 m from the edge of the source pool until spilling into the surrounding meadow (Fig. 1, Fig. S1). At the time of sampling, the outflow channel experienced a temperature gradient of 36°C and a pH gradient of 1 pH unit at the sample locations (reported in Table S1). Both the source pool and the first ∼7 m of outflow channel are encrusted (or “walled”) with siliceous sinter (Fig. S1d, e, j, i). The effect of this mineralization, affecting sites 1 and 2, is that fluid flow progresses as if through a chute with rapid flow rates of ∼0.5 m/s, with minimal interaction with material derived from the meadow surrounding the site, except when affected by a large precipitation event. The physical arrangement of the outflow channel changes drastically at site 3, continuing on through the end of the outflow and sites 4 and 5, where the outflow broadens and the edges of the outflow channel are no longer “walled” with silica, allowing flow to slow to <0.1 m/s and spread beyond the boundary of the channel (Fig. S1f, g, h, j). In fact, as of 2010 the lower outflow channel has changed direction from a northerly track shown in Figure 1, to a more easterly and southerly track. This broadening of the outflow changes flow rates, allowing fluids to cool more quickly at the shallow edges, particularly outside the thalweg. Beyond the sinter-lined portion of the system, cooled fluids from shallow reaches can remix with thalweg fluids in a direction not parallel with the flow path. This allows an opportunity for mixing with accumulated meadow fluids (pooled precipitation and condensed hydrothermal steam) containing organic material such as plants, insects, and animal excrement [2] (Fig. S1g, h, j). Fluid temperature at BP fluctuates over short (few hours) time frames, as shown by the ranges reported in Table S1, but also with diurnal and seasonal cooling.

**Figure 2:**
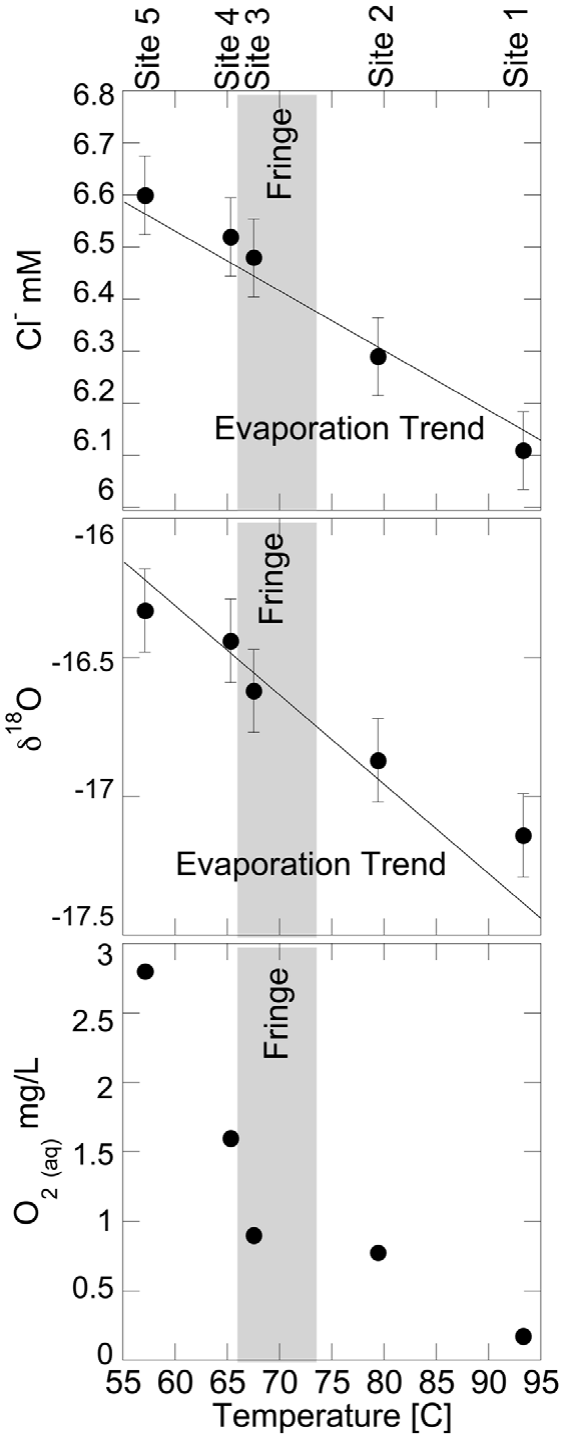
Selected geochemical trends moving downstream at BP. Top and middle; chloride and oxygen isotope (of water) measurements, respectively, showing calculated evaporation trendlines imposed on the data; the slopes of the lines are set by the extent of evaporation required to account for the temperature decrease. Bottom; dissolved oxygen concentrations, representing redox processes in BP. All plots show chemosynthetic (far right), transition “fringe” (grey bar), and photosynthetic zones (far left).

**Figure 3:**
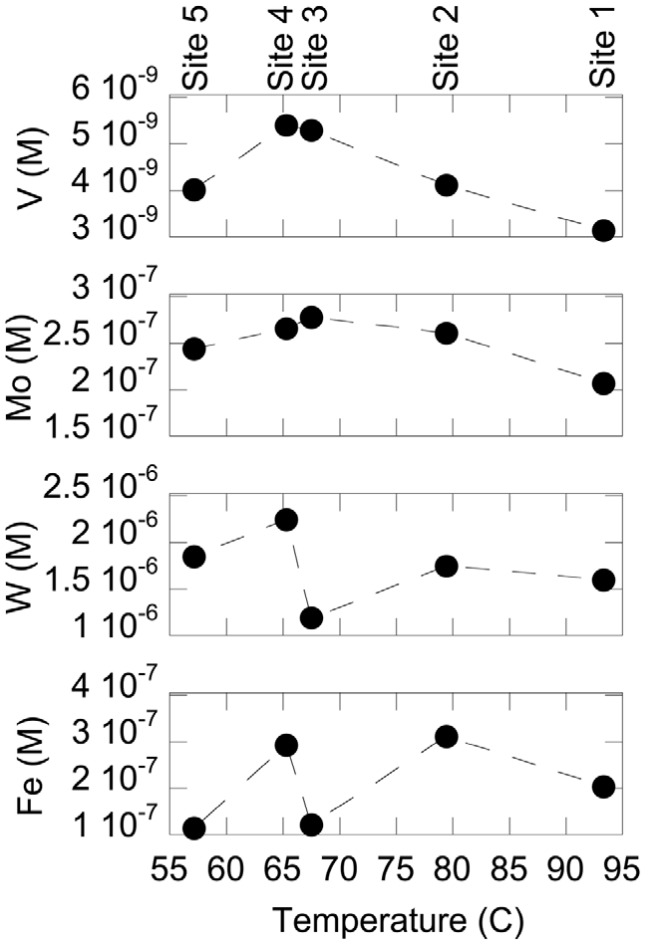
Selected aqueous trace element concentrations downstream at BP. Vanadium, molybdenum, tungsten, and iron total molar concentrations were measured for each BP sample site.

The above description of the BP outflow represents the “typical” configuration during the summer months, when we are most apt to be sampling the hot spring. Atypical events, such as very heavy precipitation, have a dramatic affect on the flow of fluid in and around BP. We have observed substantial meadow “input” (organic-rich fluids and solids) in the source pool and entire outflow channel following an hour of steady, hard rainfall (Fig. S1k, l). This enrichment from the meadow brings a heretofore unquantified amount of carbon, nitrogen, and other nutrients into the system for several days. In addition, YNP has a short warm season of 2–3 months. The shallow cooling and mixing processes may be greatly enhanced in the colder months (September-June) when the air temperature may be >60°C colder than the summer months, and indeed our observations indicate that the photosynthetic fringe moves upstream in the winter [2]. Evidence of evaporation and redox processes are readily observed in the BP geochemical dataset (full dataset given in Table S1). Downstream values of δ^18^O and conservative ions such as Cl^–^ (Figure 2) increase in accordance with the extent of evaporation required to account for the temperature decrease, while dissolved oxygen (DO) increases slightly up to site 3, likely as a result of atmospheric mixing, before a sharp increase proposed to be due to photosynthetic production of O_2_ at sites 4 and 5. Trace element concentrations in the outflow behave variably as physical and potentially biological parameters engage. For example, trace element concentrations typically rise through sites 1 and 2 due to probable evaporative effects (Figure 3). Between sites 3 and 5, where meadow influence and reduced stream flow affect the outflow channel, concentrations of measured trace elements may decrease due to dilution by meadow input or increase again with continued evaporation (e.g., W and Fe) (Figure 3). The four example trends given in Figure 3 (V, Mo, W, and Fe) are typical of trace elements measured, and concentrations appear to either follow a steady increase up to site 4, followed by a decrease at site 5 (as shown by V and Mo), or experience a sharp decrease in concentration at site 3 (as shown by Fe and W). In short, the physical and chemical nature of the outflow channel is regulated by expected processes, but may be highly variable over short distances and over time.

As a result of this physical reality at BP, the metabolic capacity shifts with distance downstream, from a strictly chemosynthetic zone in the hottest portion of the outflow (sites 1, 2) to a zone which allows growth of photosynthetic organisms (sites 4 and 5). Between these is a transition zone (site 3), previously identified as a unique ecotone wherein the precise location of the chemosynthetic-photosynthetic boundary shifts with seasonally-influenced fluid temperatures. While this ecotone boundary may be defined by fluid temperature (and possibly other environmental factors), we explore here the finer-scale changes in diversity and genetic metabolic capacity within each zone.

### II. Community Composition, Dominant Genomes, and Downstream Transitions

#### BPEG sequence analysis

The BPEG consists of a total of 474,256 reads, constituting more than 470 megabases of sequence (Table 1). Contig assembly was comparable across all five sites, ranging from 60–72% of total sequenced bases, but a high average contig size at site 1 (2480 bases) (Table 1) suggests a particularly low community diversity in this sample. On average, BPEG contigs were assembled from more than six reads, contributing to the mean 3.4 fold coverage per contig. The high-temperature sites 1 and 2 have lower GC percentages than the other three sites, reflecting a bias caused by low-GC Aquificae (discussed below).

**Table 1:** BPEG sequence statistics.

	Site 1	Site 2	Site 3	Site 4	Site 5	Total
**Reads**	74183	88801	76401	134669	100202	474256
**Average Read Length**	1111	889	1145	957	932	1007
**Total Sequence**	82446031	78909662	87473573	128927040	93352445	471108751
**GC Percentage**	49	51	59	57	58	55
**Contigs**	7725	12033	8773	19172	17998	65701
**Average Contig Size**	2480	1751	2087	1854	1698	1974
**Largest Contig**	22475	23575	20435	53450	13378	26663
**Contig Coverage**	3.9	3.2	4.2	3.0	2.4	3.4
**Reads per Contig**	8.3	6.2	7.2	5.5	4.2	6.3
**% bases in Contigs**	71.27	66.26	63.74	61.25	60.85	64.67

#### Community diversity

**Figure 4:**
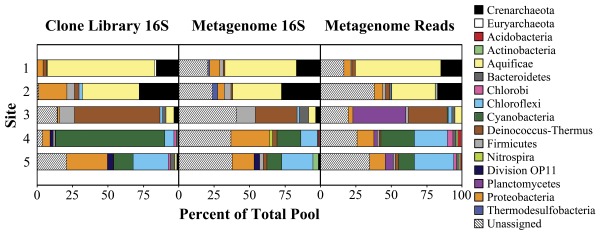
Taxonomic diversity and richness in “Bison Pool”. Left panel shows diversity and richness as measured by 16S rRNA clone libraries, middle panel shows 16S rRNA genes identified in the BPEG, and right panel shows phylum-level assignments for BPEG reads. 16S rRNA sequences from the clone library and the BPEG (metagenome) were assigned using RDP’s classifier. Unassigned sequences represent those scoring below their respective S_ab cutoff (80% for clones and 50% for metagenome sequences). The percentage of total Archaea (Crenarchaeota plus Euryarchaeota) in each clone library are shown normalized to the metagenome total. Metagenome reads were assigned using coordinated homology and tetranucleotide binning. All assignments to phyla containing less than 1% of any community are included within the unassigned.

Initial estimates of community composition and diversity were performed on a clone library of 2,359 16S rRNA sequences covering the five BP sites (Figure 4, left panel, and Figure S2). 16S rRNA data suggest that phylum-level microbial diversity (Shannon Index and Simpson Index) and evenness (Shannon Index and Pielou Index) generally increase along the outflow channel, interrupted by a large decrease in these measures at site 4 (where Cyanobacteria dominate the clone library) (Table S2).

In addition to the 16S rRNA clone library, we also probed the metagenome reads for 16S rRNA sequences in order to assess how well these two datasets agree (Figure 4, middle panel). While this *ad hoc* metagenomic 16S rRNA library is limited to organisms that compose a significant proportion of the community (statistically high enough to catch a portion of the 1,500 bp gene in a 2–8 Mb genome), more than 63 hits were found at each site. RDP analysis of these sequences identified a similar community structure to the 16S rRNA clone library, albeit with much higher uncertainty due to the small portion of 16S rRNA covered in a single Sanger read. This method does allow the quantification of archaea and bacteria in a single analysis, revealing that any archaeal 16S rRNA in the chemotrophic sites 1 and 2 all but disappear after the transition site 3 and into the phototrophic sites 4 and 5. At the phototrophic sites, the BPEG reveals communities co-dominated by the two phototrophic phyla Chloroflexi and Cyanobacteria and the chemotrophic Proteobacteria, unlike the dominant Cyanobacteria sequences in the 16S rRNA clone library, leading to a more linear trend for the diversity and evenness indices for BPEG 16S rRNA (Table S2). This comparison suggests a potential methodological bias in traditional clone library techniques, most notably the overrepresentation in the clone library for Cyanobacteria, Aquificae, and Deinococcus-Thermus, and an underrepresentation for many others (Figure 4).

The final measure of community diversity we assessed was BPEG taxonomic read assignment through homology binning and subsequent tetranucleotide analysis (Figure 4, right panel). As discussed below, the conservative nature of this assignment limited tetranucleotide analysis to only those bins with enough initial homology matches to form a confident PCA cluster. Thus, smaller bins (those containing fewer than 50 scaffolds) were analyzed using only homology binning. Despite this limitation, the binning process allowed us to taxonomically assign as much or more of the community when compared to the assignment of BPEG 16S rRNA clones, as can be seen by comparing the “unassigned” data among the three panels in Figure 4.

Binning assignments agree quite well between 16S rRNA clone and BPEG 16S rRNA analysis with one major exception. Site 3, which has the highest percentage of uncharacterized BPEG 16S rRNA sequences, contains a previously unidentified phylum-level bin related to the Actinobacteria and Planctomycetes–a consequence of relatively few homology hits to these two taxa (labeled as Planctomycetes in Figure 4 due to the absence of significant Actinobacteria hits at sites 4 & 5). Thirty nine 16S rRNA clones shared greater than 99% identity to a single 16S rRNA sequence found in this bin, and all were unassigned at a phylum-level using RDP (out of 53 total unassigned at site 3), and most fell well below the suggested 80% S_ab score cutoff (averaging 44%). With <84% 16S rRNA nucleotide identity to several sequenced Actinobacteria species and <92% identity to other environmental clones, this organism likely constitutes a unique phylum- or class-level classification related to the Actinobacteria or Planctomycetes.

#### Homology binning of BPEG nucleotides

The utility of homology-based methods in binning environmental metagenome reads is highly dependent on the availability of relevant reference genomes. A relative dearth of completed genomes from hydrothermal systems limits the number of closely matched sequences, especially in the hottest sites 1 and 2. Due to a lack of any available useful reference genomes for comparison, a major community member at site 3 was entirely missed by using homology binning (Table 2). We analyzed homology binning strictness thresholds that ranged from general (phylum-level binning) to stringent (genus-level binning) in order to understand the level of conservation in BPEG sequences compared to their closest sequenced relatives.

**Table 2:** BPEG binning and consensus genome statistics.

		Read assignment (genus)	Read assignment (phylum)	Read assignment (tetra)	Proteins	Sequence (Mb)	% of total sequenceat site	Estimated genomesize (Mb)	Depth	Coverage	average % aa identity to closest reference	Closest Reference(by % aa identity)
**Site 1**	Aquificae	11287	40875	44806	18005	50.1	60.7%	1.6	31.28	100.0%	67.8%	*H. thermophilus* TK 6
	Crenarchaeota	4854	10599	11065	6656	12.3	14.9%	2	6.13	99.8%	74.4%	*P. islandicum* DSM 4184
	Deinococcus	1643	1745	1881	1718	2.0	2.4%	2.1	0.96	61.6%	81.7%	*T. scotoductus* SA 01
	Proteobacteria	2092	3455	3743	3801	4.2	5.1%	5	0.84	56.9%	84.5%	*E. cloacae* ATCC 13047
**Site 2**	Aquificae	6694	24489	27138	10649	24.8	31.4%	1.6	15.49	100.0%	67.5%	*H. thermophilus* TK 6
	Crenarchaeota	5951	14710	15274	7989	13.6	17.2%	2	6.80	99.9%	74.0%	*P. islandicum* DSM 4184
	Deinococcus	2054	2256	2686	2098	2.4	3.0%	2.1	1.13	67.6%	81.7%	*T. thermophilus* HB8
	Proteobacteria	2780	4799	5279	4709	4.8	6.1%	5	0.96	61.6%	82.1%	*E. cloacae* ATCC 13047
**Site 3**	Actinobacteria-Planctomycetes	0	895	28374	11239	33.4	38.2%	5	6.67	99.9%	45.0%	*R. xylanophilus* DSM 9941
	Aquificae	287	3118	3780	3337	4.4	5.0%	1.6	2.73	93.5%	66.6%	*H. thermophilus* TK 6
	Chloroflexi	1051	1904	1950	1560	2.3	2.6%	5.8	0.39	32.4%	94.3%	*T. roseum* DSM5159
	Deinococcus	19526	20371	20778	8958	23.1	26.4%	2.1	10.98	100.0%	82.8%	*T. thermophilus* HB27
	Proteobacteria	344	2392	2557	1711	3.0	3.4%	5	0.60	44.9%	76.6%	*E. cloacae* ATCC 13047
**Site 4**	Chlorobi	613	3086	5263	3135	5.1	3.9%	3.3	1.54	78.5%	58.2%	*C. thalassium* ATCC35110
	Chloroflexi	27869	30089	30855	19487	29.6	23.0%	5.8	5.11	99.4%	96.6%	*Roseiflexus* RS-1
	Cyanobacteria	26458	30674	31497	15381	29.8	23.1%	3	9.94	100.0%	98.2%	*Synechococcus* sp. B’
	Deinococcus	940	1329	1614	1383	1.6	1.2%	2.1	0.74	52.4%	64.7%	*T. thermophilus* HB8
	Planctomycetes	0	471	3185	2332	3.1	2.4%	5	0.61	45.7%	53.7%	*G. obscuriglobus* UQM2246
	Proteobacteria	6883	11636	15752	7922	15.0	11.6%	5	3.00	95.0%	88.6%	*E. cloacae* ATCC 13047
**Site 5**	Chlorobi	291	1418	1883	1508	1.8	1.9%	3.3	0.54	42.0%	58.2%	*C. thalassium* ATCC35110
	Chloroflexi	24638	26850	27462	17247	25.9	27.8%	5.8	4.47	98.9%	91.6%	*Roseiflexus* RS-1
	Cyanobacteria	9120	10828	11217	7382	10.6	11.4%	3	3.54	97.1%	96.7%	*Synechococcus* sp. A
	Deinococcus	1339	1790	2207	2128	2.0	2.2%	2.1	0.97	61.9%	76.9%	*T. scotoductus* SA 01
	Planctomycetes	0	360	5539	4021	5.2	5.6%	5	1.04	64.7%	55.3%	*G. obscuriglobus* UQM2246
	Proteobacteria	6778	9734	10951	7552	10.3	11.0%	5	2.06	87.3%	88.7%	*E. cloacae* ATCC 13047

* Read assignment. ^1^With the exception of homology binning information, all other statistics shown use phylum-level tetranucleotide binning data.

Genus-level homology binning assigned less than 19% of total BPEG sequence data in site 1; lowering the specificity to family-level increased overall assignment to nearly 55%. This indicates that members of the 92°C site 1 community share, at best, only a family-level relationship with available reference genomes. Indeed, site 1 sequences assigned to bins average only 65–70% nucleotide identity with reference sequences and just 51–68% amino acid identity with best reciprocal protein hits (Table 2). Conservative phylum-level binning assigned 40% of bases to the Aquificae, 12% to the Crenarchaeota, and 4% to the Proteobacteria. These numbers changed little over the phylum–family assignment level. In contrast, phylum/class/order-level assignment at site 2 assigns only 17% of bases of Aquificae, 10% to the Crenarchaeota, 3% to the Proteobacteria, and 3% to the Deinococcus-Thermus, suggesting a community shift toward the poorly understood (at least with regards to sequenced genomes) ‘ecotone’ community at site 3 (described below).

The dominant microbial taxon in the site 3 BPEG is a member of the phylum Deinococcus-Thermus, accounting for less than 18% of total nucleotides at each taxonomic assignment level. The assigned sequences match reasonably well with sequenced *Thermus* genomes, ∼74% nucleotide identity, but may be too distant for a genus-level taxonomic assignment. The Deinococcus-Thermus bin was also the only major BPEG component with a number of BLAST matches to a previous YNP metagenome project [9], >80% nucleotide identity to the *Thermus*-dominated Calcite Springs. Other site 3 community members account for only a small portion of the remaining sequences: notably Proteobacteria at 5% and Aquificae at 5%. The phylum-level unassigned sequence percentage, 63%, is the highest among all sites. At the time of sampling, and as previously reported by Meyer-Dombard et al. [4], site 3 represented a fringe “ecotone” between the strictly chemosynthetic and photosynthetic zones of the outflow channel. This ecological condition may present metabolic opportunities that explain the unique nature of the site 3 metagenome.

Genus-level binning assigns more than 62% of reads in the site 4 BPEG and nearly 58% at site 5, with no improvement below the family-level. This is primarily due to two phyla at sites 4 and 5 that are well-represented in sequenced genomes, Cyanobacteria and Chloroflexi, which share as much as 80–85% nucleotide identity with their respective BPEG sequences. Chloroflexi account for 23% and 28% of bases in site 4 and 5, respectively, while Cyanobacteria account for 23% and 11%, respectively, in the same two sites.

#### Tetranucleotide binning

Like the homology binning described above, tetranucleotide binning was performed at five major taxonomic levels (phylum, class, order, family, genus). Tetranucleotide binning dramatically improved BPEG sequence assignment at the genus-level, which was often too specific for the homology binning technique, and the percentage of assigned reads remained consistent with assignment at every taxonomic level between genus and phylum, where homology binning was most successful (Table 2). In this way, homology and tetranucleotide binning are complementary, as each has strengths where the other is weakest.

Site 1, where genus-level homology binning was weak, showed the most dramatic improvement using tetranucleotide binning. Tetranucleotide binning revealed three major clusters correlating to the Aquificae, Crenarchaeota, and Proteobacteria (Figure 4). The Aquificae cluster contains matches that are most closely related to three genera, *Hydrogenobacter*, *Sulfurihydrogenibium*, and *Thermocrinis*, and accounts for 60% of all site 1 bases (Table 2). The next largest cluster, Crenarchaeota, most closely matches the genera *Pyrobaculum* and *Thermoproteus*, members of order Thermoproteales, with 15% of all bases accounted for, similar to the percentage assigned using order-level homology binning. Site 2 produced similar results, with a large cluster around an Aquificae bin (31%) and a smaller cluster for Crenarchaeota (17%). However, 38% of site 2 bases remain unassigned, the highest at any BPEG community. Proteobacterial sequences at site 1 share the same best reciprocal match, *Enterobacter cloacae* ATCC 13047 [40], with all other BPEG sites, suggesting this taxon is potentially either cosmopolitan or an outside contaminant.

Genus-level homology binning at site 3 yielded a relatively low percentage of assigned sequences (Table 2). However, phylum-level tetranucleotide binning succeeded where previous methods failed in identifying a previously unidentified cluster labeled only as Actinobacteria-Planctomycetes (Figure S3). This cluster, representing an organism functioning as a specialist at site 3 and defined by just a few homology-binned sequences, is distinct from other taxonomic groups and easily assigned using tetranucleotide methods.

Homology binning assigned two distinct taxonomic groups at site 4 and 5, and even our conservative tetranucleotide binning cutoffs recruited, on average, just 6 additional sequences for these bins at sites 4 and 5. This indicates that two of the most dominant BPEG phyla, Cyanobacteria and Chloroflexi, contain relatively few unique genes in relation to reference sequenced genomes.

#### Protein clustering and annotation of the BPEG dataset

**Figure 5:**
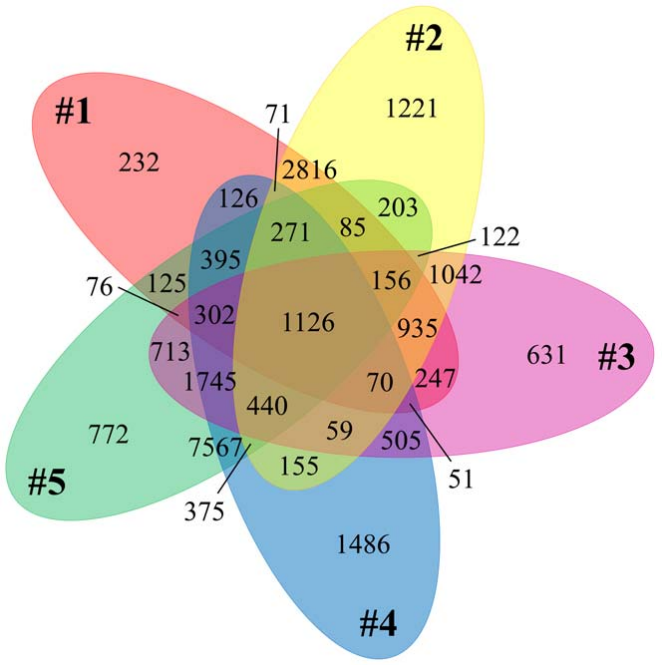
Distribution of Markov protein families in “Bison Pool”. 5-way Venn diagram of the overlap between protein families containing at least one BPEG sequence.

Markov clustering of protein families yielded 38,352 clusters containing more than a single protein, though 14,232 clusters were composed entirely of KEGG database sequences. The conservation of protein families between sites is shown in Figure 5. The two largest overlaps occur in the chemotrophic site 1 & 2 pair (2,816 protein families) and the phototrophic sites 4 & 5 (7,567 protein families), with the latter being nearly 3-fold larger than the former. This disparity likely stems from the larger protein library in the relatively large Chloroflexi/Cyanobacteria genomes in the phototrophic sites versus the smaller Aquificae/Crenarchaeota genomes in chemotrophic sites.

Although site 3 shares a stronger taxonomic similarity to sites 1 and 2–including the presence of Aquificae and Crenarchaeota–there are twice as many protein families common to sites 3, 4, and 5 (1745 families) than sites 1, 2, and 3 (935 families) (Figure 5). This disparity between metabolic and taxonomic diversity is most likely due to the conserved heterotrophic organisms present primarily in sites 3, 4, and 5 (see below).

The unique metabolic potential at any given site is markedly different moving down the BP outflow. Sites 2 and 4 contain a full order of magnitude more Markov protein families than their neighboring sites (1221 and 1486 versus 232, 631, and 772 in Figure 5). This wealth of unique sequence at sites 2 and 4 may be due to a relatively large population of a particular phylum at each site (Crenarchaeota and Cyanobacteria, respectively, Figure 4), or the larger diversity of species within each phylum.

#### Primary taxonomic bins: Reconstruction of consensus genomes of dominant organisms

The combination of homology binning and tetranucleotide analysis yielded several bins at each site with high enough putative coverage (Table 2) to reconstruct putative genomes and, ultimately, metabolic networks; we term these genomes “consensus genomes” due to the fact that they represent a composite view of the heterogeneous BP community members that fall within a given phylum. Our data suggest that most of these bins represent single organisms within a given phylum, showing a range of species- and strain-level diversity. Eight of these bins contained enough reads to cover greater than 99% of their predicted genome size (based on their nearest sequenced neighbor), allowing assignment of consensus genomes. However, the lack of a long paired-end sequence library and the non-clonal environmental sequences prevented the construction of single, contiguous genome assemblies, even in the most well-sampled bins. In total, more than 320 Mb of the BPEG (68%) is located in 25 bins, containing upwards of 83% of the reads from a single site.

The most complete coverage for a single organism was the Aquificae consensus genome at site 1. Over 50 Mb of sequence data suggests a genome coverage of over 30×, due to the relatively small size of most sequenced Aquificae (∼1.6 Mb). Proteins from the site 1 Aquificae match equally well to *Hydrogenobacter thermophilus* TK-6 [41] and *Thermocrinis albus* DSM 14484 [42], at ∼67% amino acid identity in reciprocal best hits, with lower homology to *Aquifex aeolicus* VF5 [43] (61%) and the two closest *Sulfurihydrogenibium* genomes (51%). The best match to the site 1 Aquificae consensus genome was, in fact, the 25 mb Aquificae consensus genome from site 2, which shared greater than 95% amino acid identity of reciprocal best hits, suggesting that these two sites may share nearly identical ecotypes. The relatively low identity to sequenced species reinforces previous suggestions that many hot spring Aquificae belong to a new clade, quite distant from cultured Aquificae [44].

Sites 1 and 2 both contain a Crenarchaeota consensus genome that was covered at ∼6× depth (at each of the two sites) in the BPEG. This group, which matches most closely with order Thermoproteales, particularly the genera *Pyrobaculum* and *Thermoproteus*, likely represents a new, uncultured genus within this order functioning as a specialist in the hottest zones of the hydrothermal system. While the Crenarchaeota proteins from both sites share the highest number of reciprocal protein matches with *Thermoproteus neutrophilus* V24Sta (81% of the genome versus 79% for *Pyrobaculum islandicum* DSM 4184), the amino acid identity is slightly higher in *P. islandicum*. 16S rRNA homology indicates that this species is most closely related to uncultured Thermoproteales identified from previous YNP hot spring studies [3], where they were implicated in the reduction of sulfate. This metabolic capability is further reinforced by a number of sulfate reduction genes assigned to this consensus genome (discussed below).

The unique nature of the transition site 3, as discussed by Meyer-Dombard et al. [4], is confirmed by the fact that neither of the two dominant phylum bins at this site represent >6% of the composition at any other BP site. The taxonomic identities of these two bins are quite distinct: one is a Deinococcus-Thermus species with sequence identity high enough to belong to the genus *Thermus*; while the other is a bin only nominally identified as Actinobacteria-Planctomycetes by virtue of a relatively low number of homology matches to these two phyla. With only 82% 16S rRNA identity to any sequenced organisms, this Actinobacteria-Planctomycetes consensus genome likely represents a novel member of a new phylum only distantly related to these two phyla. Amino acid identity to its nearest sequenced matches is consequently low and only represents a small portion of the reference genomes (fewer than 25% of *Rubrobacter xylanophilus* DSM 9941 proteins). Preliminary analysis of genes within this bin predicts that this species is Gram-positive, aerobic, and chemoheterotrophic by the presence of cytochrome *c*-oxidase and key enzymes in oxidative phosphorylation and glycolysis/gluconeogenesis, as well as the absence of any apparent lithoautotrophic metabolism. BLAST results from the site 3 consensus genome against the other BPEG sites suggests that this organism is also prevalent in sites 4 and 5, preliminary comparisons to site 3 Actinobacteria-Planctomycetes sequences suggests that it may account for up to 15% of community sequence at sites 4 and 5, but was only nominally detected in sites 1 and 2. Targeted culturing and direct biochemical analysis will hopefully reveal more about the nature of this novel hot spring phylum.

At a glance, the presence of a small number of site 3 reads classified as Chloroflexi suggests that some overlap with the phototrophic communities could be occurring at this transition site. However, upon closer analysis, we discovered that site 3 Chloroflexi proteins are vastly different from those prevalent at the phototrophic sites 4 and 5. Unlike the sequences from sites 4 and 5, which match closely to those from the sequenced *Roseiflexus* strain RS-1, site 3 reads match strikingly well to *Thermomicrobium roseum* DSM 5159 [45], an aerobic, non-phototrophic organism that was only recently moved to phylum Chloroflexi [46]. Conversely, Chloroflexi proteins from sites 4 & 5 match greater than 82% of the *Roseiflexus* RS-1 protein library. Many of the proteins unique to the BPEG bins (in relation to *Roseiflexus*) are present in *Chloroflexus aurantiacus* J-10-fl [47], suggesting that this bin may represent a mix of *Chloroflexus*/*Roseiflexus* ecotypes or a single ecotype that is a transition point or early branch between these two genera. Among these proteins are arsenite reductase and aerobic CO dehydrogenase, suggesting that these enzymes may play a role in the survival of BP Chloroflexi.

Cyanobacteria consensus genomes from sites 4 & 5 present further evidence of the dominance of the particular clade of *Synechococcus* previously sequenced from other YNP hot springs [48]. These bins represent the only case at BP where there is a detectable strain-level community shift between two adjacent sites in the BPEG data. Our analysis reveals a community shift from *Synechococcus* sp. strain B-prime prevalence at site 4 to *Synechococcus* sp. strain A at site 5 (Table 2). In fact, unlike the Aquificae consensus genomes from sites 1 and 2, Cyanobacteria at sites 4 & 5 match more closely to their sequenced reference genomes than they do to one another (>96% amino acid identity versus 88%, respectively). It should be noted that this temperature relationship trends in the opposite direction as observations by Bhaya et al., where strain A typically dominated at higher temperatures [48]. However, variable chemistry and ∼10 degree temperature ranges at each sampling site likely account for this disparity rather than a temperature effect, as both sites 4 and 5 are within the published ranges for both strains. The absence of B-prime-specific genes for phosphonate utilization and cyanophycin synthesis in site 5 reinforces our observation of this role-reversal; however, the presence of some B-prime genes in site 5 suggests that either both sites contain a B-prime-like strain, or species present at BP represent new strains carrying characteristics of both A and B-prime.

Our ability to reconstruct genomes of dominant organisms at each location allows assignment of genes affiliated with metabolic function to specific organisms. In effect, the BPEG provides not only the metabolic potential at each site, but identifies which organisms possess that potential. The coordination of this genomic database with geochemical and environmental data is discussed in the following section.

### III. Conceptual Modeling of the Metabolic Landscape of BP

#### The chemo(litho)trophic buffet

As a means of evaluating the energy available to chemolithotrophs at BP sites 1–5, the chemical affinity (A_r_) was estimated for hundreds of reactions (see Table S3). For a full review of A_r_, readers are advised to consult Shock et al. [30]. Briefly, the chemical affinity for the r^th^ reaction is related to the familiar concept of the change in the overall Gibbs energy of a chemical system, or ΔG, with respect to the progress of the r^th^ reaction (ξ_r_)
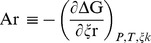
and quantified by comparing the equilibrium constant for a reaction (K_r_) and the activity product (Q_r_) for the reaction via



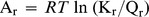
By definition, energy availability is indicated by positive values of A_r_.

In the BP outflow, the pH of the fluids only varies by ∼1 pH unit. As a consequence, large changes in the energy availability as a function of downstream sampling are only estimated for ∼15% of the reactions evaluated. If A_r_ values are ranked for each reaction from most positive to most negative (see Table S4), there is little variation among the top ten reactions that yield the most energy at each site, although the order of these reactions shifts slightly downstream. Reactions 1, 5, 10, 11, 13, 14, 15, 18, and 19 fall among the ten most energy-yielding reactions for all five locations, for example. Furthermore, we see that the top fifteen reactions all require O_2_ as an oxidant, regardless of reductants involved. In general, the order of the most effective oxidants at all sites is O_2_, NO_3_^−^, NO_2_^−^, S°. At site 1, the lower ranking reactions typically use SO_4_^−2^, magnetite, hematite, goethite, CO, CO_2_, and pyrite as oxidants (in that order), and this is essentially the same at the end of the outflow at site 5, with CO and CO_2_ ranking somewhat higher than the iron minerals. In general, all reactions utilizing the oxidant O_2_, and most reactions using the oxidants NO_3_^−^, NO_2_^−^, and S°, yield energy at all locations. In fact, the oxidation of CH_4_, H_2_S, and magnetite generally only yield energy when O_2_, NO_3_^−^, and S° are the oxidants, with a few exceptions. Chemical affinities for Fe^+2^ oxidation are almost always positive, particularly toward the end of outflow. Typically, there is no energy to be gained at BP via the following: reactions using pyrite as an oxidant, reactions where SO_4_^−2^, CO or CO_2_ are reduced with NH_4_^+^ or NO_2_^−^, reactions reducing magnetite, hematite, or göetite (with a few exceptions), or reactions oxidizing NH_4_^+^ or NO_2_^−^ (except when O_2_ is the oxidant).

Potentially more interesting are chemolithotrophic reactions where A_r_ changes from negative to positive, or A_r_ values that vary considerably across locations. As an example, we can examine the reduction of SO_4_^−2^ (reaction numbers 119–162). When Fe^+2^ is used to reduce SO_4_^−2^ the energy yield increases downstream, sometimes moving from no energy available at site 1 (reactions 123–125), to positive (although paltry) A_r_ at site 5, or changing by an order of magnitude from source to beginning of the runoff channel (reactions 153–155). In contrast, when CO or CH_4_ are used to reduce SO_4_^−2^, A_r_ decreases downstream, sometimes going from positive to negative (e.g. reactions 131, 143, 159–161).

#### Metabolic complexity downstream at BP

Reactions that hover around the equilibrium (A_r_ = 0) have the potential to dictate differences in community composition between locations. If there is no energy to be exploited by catalyzing a reaction, there should be no organisms that use that reaction for energy. Organisms with multiple metabolic talents, which can take advantage of variable energy regimes, *may* have an advantage over specialists at particular points in the outflow channel. A metabolic map of the BP locations can be proposed based on the metagenomic and geochemical revelations presented here. Sites 1 and 2 present environments where hyperthermophilic chemolithoautotrophs and chemoheterotrophs compete for nutrients and other resources. In fact, Windman et al. [49] report that values of A_r_ calculated for the oxidation of the simple organic acid formate (using, for example, O_2_, NO_3_^−^, or NO_2_^−^ as oxidants) at the pH of BP are comparable to those predicted for the chemolithoautotrophic reactions presented here, suggesting that both chemolithoautotrophy and chemoheterotrophy may be equally profitable at sites 1 and 2. Further downstream at site 3, the fluid has cooled such that lower-temperature thermophiles take advantage of fluid mixing and meadow influences. Here, the temperature is low enough that chemoheterotrophs and photoautotrophs likely have a competitive edge over the strict chemolithoautotrophs, though we see no evidence of phototrophy at site 3 in the BPEG. Sites 4 and 5 are dominated by phototrophs and chemoheterotrophs, with chemoautotrophs likely inhabiting only the lower layers of photosynthetic mats. The community response to this shifting metabolic landscape is the enabling of specialists to inhabit distinct niches (e.g. Crenarchaeota at sites 1 and 2, *Thermomicrobium* at site 3, or Cyanobacteria at sites 4 and 5), as well as heterotrophic generalists with broad environmental tolerances (e.g. Proteobacteria, *Thermus*, and Firmicutes). This geochemical model of the metabolic potential at BP aligns with the identity of organisms and their metabolic capacity as predicted from the BPEG, as discussed below.

#### The sulfur cycle

**Figure 6:**
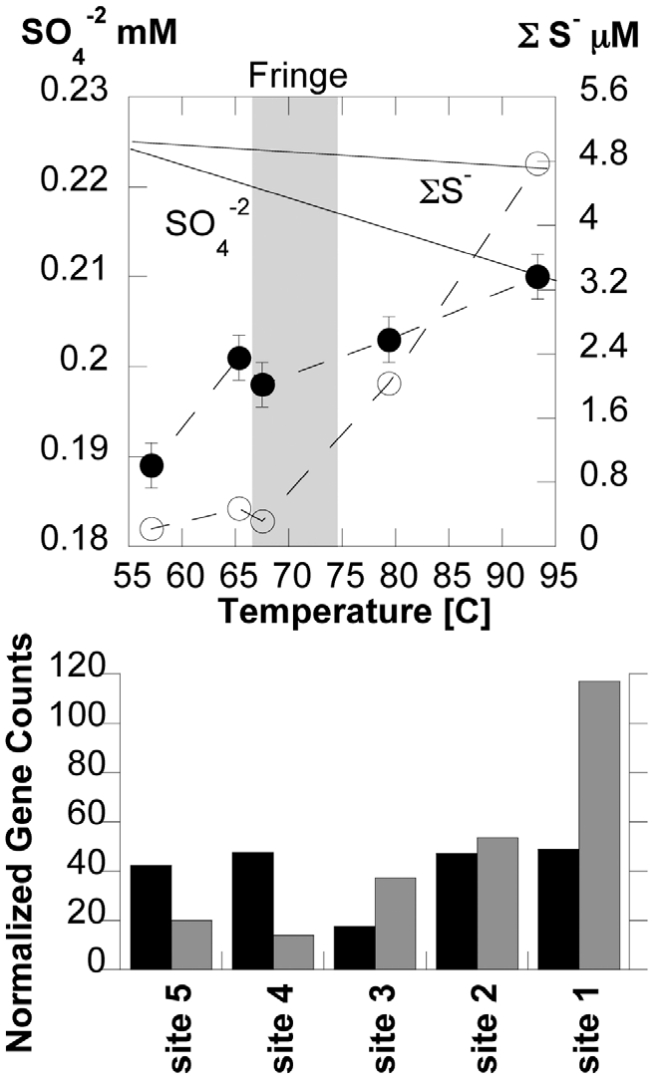
Sulfur cycle in BP. Top plot; concentrations of sulfate (solid circles) and “total sulfide” (open circles), which includes H_2_S and HS^–^, as a function of downstream flow. Calculated evaporation trends are shown (solid lines). Plot shows chemosynthetic (far right), transition “fringe” (grey bar), and phostosynthetic zones (far left). Bottom histogram shows total counts of genes associated with sulfate reduction (black bars) and sulfide oxidation (grey bars), normalized to the smallest total dataset. Sulfate reduction genes counted include desulfite reductase (*dsr*), phosphoadenosine phosphosulfate reductase (*apr*), and sulfate adenyltransferase (*sat*). Sulfide oxidation genes counted include sulfite oxidoreductase (*sor*), adenylylsulfate:phosphate adenylyltransferase (APAT), sulfide oxidase (*soxABCDXYZ*), thiosulfate quinone oxidoreductase (*tqr*), sulfide quinone reductase (*sqr*), and sulfide dehydrogenase flavocytochrome (*fcsd*), but did not include the *dsr*, *apr*, and *sat* genes which are used in both sulfide oxidation and sulfate reduction.

Overall concentrations of SO_4_^−2^ and total sulfide decrease with downstream sampling, and are examples of ions that do not appear to respond to strictly abiological influences in the runoff channel. Variation in sulfate and total sulfide concentrations between the source pool and site 5 are far below what is predicted by evaporation models, indicating that biological intervention is likely (Figure 6). Total sulfide decreases at a rate that is too high for abiological oxidation as found in previous studies [50], [51], and concentrations of sulfate do not in total account for the loss of sulfide. The largest loss of total sulfide in the BP outflow occurs between sites 1 and 2 (Figure 6). In general, there is energy available via H_2_S oxidation throughout BP, but only minimal energy available for SO_4_^−2^ reduction with inorganic reductants (Table S3). For example, oxidation of H_2_S or S with O_2_, NO_3_^−^, or NO_2_^−^ is estimated to yield between 7.3–24.3 kcal/(mol e^­^ transferred) across all sites sampled. Conversely, the reduction of SO_4_^−2^ is rarely energy yielding in BP, yielding at most 3.4 kcal/mol e^−^ transferred, written as strictly a chemoautotrophic process. There is also ∼6 kcal/mol e^−^ transferred if SO_4_^−2^ is coupled to formate oxidation in ∼pH 8 environments in Yellowstone [49], and sulfate reduction coupled to other organic compounds (such as acetate) are likely energetically favorable. Using the results from BPEG phylum-level binning, as described above, we are able to assign genes associated specifically with sulfur cycling and metabolism to specific dominant taxa (Table S5). Total counts of identified genes associated with S oxidation and reduction in the BPEG consensus genomes are shown in Figure 6. The total sum of S oxidation genes at site 1, which includes the genes *sor*, APAT, *sox*, *tqo*, and *sqr*, is more than twice that found at site 2. At sites 1 and 2, the Aquificae consensus genomes appear to have the genetic capacity for H_2_S oxidation, as *soxABXYZ* were identified, in addition to other S-oxidation genes. These Aquificae appear to lack the *soxCD* genes, which indicates they likely possess a version of the “branched” S-oxidation system and may have the capacity to deposit sulfur globules internally (periplasmic deposition), or externally to their cellular membranes [52]. At site 3, the role of S-oxidation passes to the Deinococcus-Thermus consensus genomes, which possesses a variety of S-oxidation genes, including *sor*, *soxABCDF*, *fcsd*, and *dsr*, *apr*, and *sat*. The latter three genes are also used in SO_4_^−2^ reduction, and while the Deinococcus-Thermus at site 3 does not have a full complement of S-oxidation genes, it is likely that these organisms are capable of S-oxidation in some capacity. Members of both Thermus and Aquificae are known to oxidize S in pure cultures [53]. The only apparent genetic capacity for chemotrophic SO_4_^−2^ reduction is shown by the Thermoproteales consensus genomes identified at sites 1 and 2. These organisms have all three critical genes for SO_4_^−2^ reduction (*dsr*, *apt*, and *sat*), and no genes associated strictly with S-oxidation. In pure cultures, strains of *Pyrobaculum* and other Thermoproteales are known to reduce SO_4_^−2^ coupled to the oxidation of organic carbon. While discussion has thus far been focused primarily on the strictly chemotrophic portions of the BP outflow channel, the Cyanobacterial, Chloroflexi, and Proteobacterial species at sites 4 and 5 also have copies of *dsr*, *apt*, and *sat*, indicating the capacity for SO_4_^−2^ reduction within the photosynthetic mat communities.

#### The nitrogen cycle

**Figure 7:**
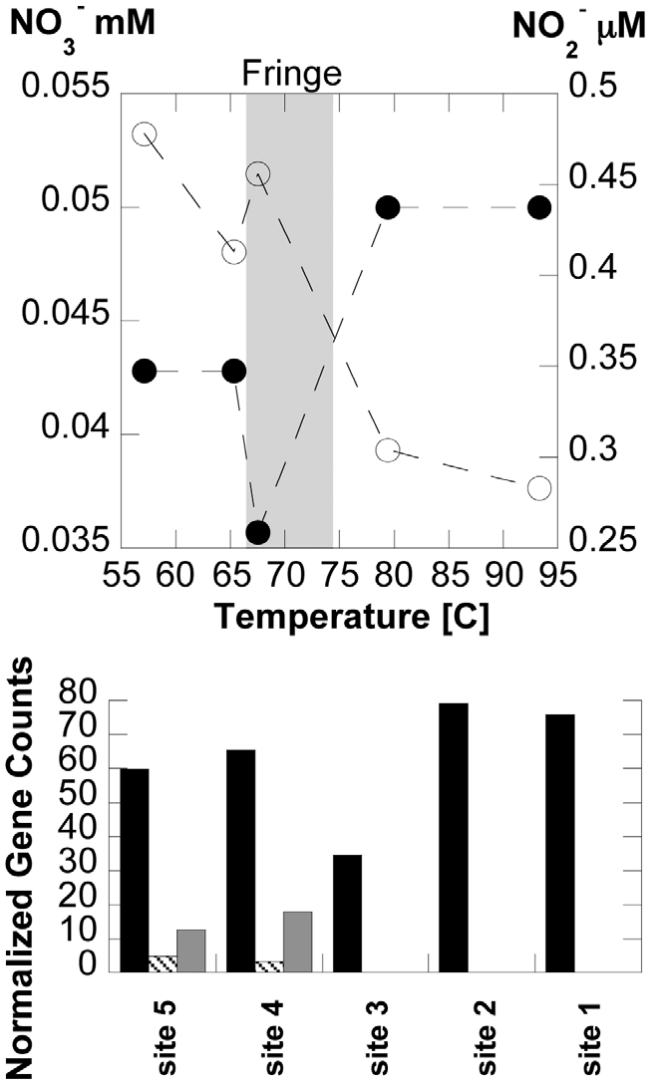
Nitrogen cycle in BP. Top plot; concentrations of nitrate (solid circles) and nitrite (open circles) as a function of downstream flow. Plot shows chemosynthetic (far right), transition “fringe” (grey bar), and phostosynthetic zones (far left). Bottom histogram shows total counts of genes associated with denitrification (black bars), N_2_-fixation (grey bars), and nitrification (diagonal filled bars) normalized to the smallest total dataset. Denitrification genes included nitrate reductase (*nar*), nitrite reductase (*nir*), nitric oxide reductase (*nor*), and nitrous oxide reductase (*nos*). N_2_-fixation was counted by nitrogenase (*nif*), and nitrification genes included hydroxylamine oxidase *hao* and ammonia monooxygenase (*amo*, which was not found in any sample).

Concentrations of NO_3_^−^ and NO_2_^−^ at the time samples were taken for the BPEG analysis are shown in Figure 7. Nitrate concentrations decrease from 0.045 mM at sites 1 and 2 to 0.035 mM at the site 3 transitional fringe environment before increasing again in the photosynthetic zone. Simultaneously, NO_2_^−^ increases from ∼0.3 µM to ∼0.5 µM. Ammonia is below detection limits in BP fluids using the spectrophotometric methods described. While not all the nitrogen as NO_3_^−^ lost is accounted for as NO_2_^−^, these data may indicate microbial reduction of NO_3_^−^ in the outflow channel.

Querying the consensus genomes constructed at each site, we have identified the genetic capacity of specific taxa for nitrogen cycle processes (Table S5). As shown in Figure 7, total counts of genes associated with denitrification and NO_3_^−^ reduction (*nar*, *nir*, *nor*, *nos*, *nap*, *nrf*) are high throughout the outflow, though site 3 has half the number of genes or less when compared to the other sites. In the chemosynthetic zone samples, the Deinococcus-Thermus, Aquificae, and Thermoproteales consensus genomes have only *nar* and *nir*, indicating a capacity for NO_3_^−^ and NO_2_^−^ reduction, but not full denitrification, similar to findings in other alkaline systems in the Great Basin [54]. It is possible that these genes may allow the use of NO_3_^−^ and NO_2_^−^ as oxidants in energy production at these locations. Indeed, the use of NO_3_^−^ as an oxidant (Table S3, reactions 21–54) is almost always energetically favorable, including several reactions in the 20 highest energy yielding reactions (Table S4), yielding between 2–17.5 kcal/mol e^−^ transferred. Further down the outflow channel, the full suite of denitrification genes are only found in the “unassigned” bin, indicating that their organism(s) of origin has not been determined and that no single taxon has the capacity for full denitrification.

The ability to oxidize NH_4_^+^ or NO_2_^−^ at BP does not seem likely, as predicted by the BPEG. The *amo* genes were not found at any location, and hydroxylamine oxidase (*hao*) and *nor* were only found in “unassigned” bins in the photosynthetic zones. Unlike other terrestrial hot spring systems [55], [56], no evidence was found for *Nitrosocaldus* sp. in the 16S rRNA or BPEG datasets, including associated *amo* genes. It should be noted that the oxidation of NH_4_^+^ and NO_2_^−^ seldom yields energy in BP, the exception being reactions using O_2_ as the oxidant, which yields only between 7–10 kcal/mol e^−^ transferred (Table S3). Therefore, the increase in nitrite in Figure 7 could be interpreted as a result of NO_3_^−^ reduction, rather than NH_4_^+^ oxidation. Additional processes must be responsible for the decreasing concentrations of NO_3_^−^ down-channel, the most likely being denitrification and/or incorporation into biomass. Another possible outside source of nitrogen for BP is N_2_-fixation occurring at sites 4 and 5, quantified by the presence of the *nifH* gene assigned to Chloroflexi and Cyanobacteria consensus genomes at these locations. This agrees with previously reported nitrogen isotopic values in the BP biofilms [2], which indicate that nitrogen is recycled throughout the chemosynthetic zone of the outflow, becoming increasingly isotopically enriched down the outflow, but approaching atmospheric isotopic ratios within the photosynthetic zone as nitrogen fixation occurs. In summary, genetic and geochemical evidence for nitrification in the outflow or nitrogen fixation in the chemosynthetic zone are lacking. Further, genetic and isotopic evidence for denitrification/nitrogen recyling throughout the outflow, and the agreement between genetic and geochemical datasets that nitrogen fixation is occurring in the photosynthetic zone, support the coupling of genetic and geochemical datasets as a powerful tool for understanding biogeochemical processes in natural systems.

#### Carbon fixation and heterotrophy

**Figure 8:**
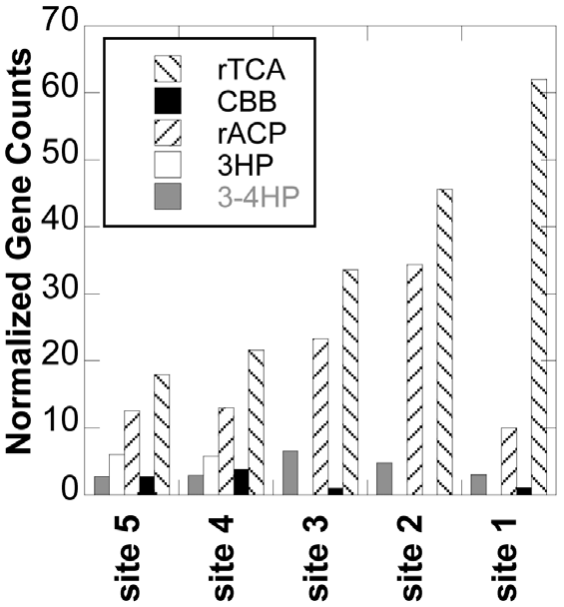
Total counts of genes associated with carbon-fixation via five cycles/pathways. Shown are genes associated with the reductive tricarboxylic-acid cycle (rTCA), including citryl co-A synthase/lyase, and pyruvate ferridoxin oxidoreductase (*oorABCD*). The Calvin cycle (CBB) is represented by the gene for ribulose-1,5-bisphosphate carboxylase, and the reductive acetyl Co-A pathway (rACP) is estimated by CO dehydrogenase. Malonate semialdehyde reductase and 4-hydroxybutyryl-CoA dehydratase are used as proxies for the 3-hydroxypropionate cycle (3-HP), and the 3-hydroxypropionate/4-hydroxybutyrate cycle (3-4HP), respectively. All columns are normalized to the smallest total dataset.

Concentrations of dissolved organic and inorganic carbon (DOC, and DIC) vary little downstream at BP, ∼5 ppm C and ∼80 ppm C, respectively. In Figure 8, total counts of genes within the consensus genomes associated with five of the six recognized CO_2_-fixation schemes are given, outlining a progression of CO_2_-fixation processes as a function of downstream community shifting (Table S5). At site 1, the hottest, strictly chemotrophic location, the majority of carbon cycling genes found in the BPEG are related to the reverse tricarboxylic acid (rTCA) cycle. Here, evidence for carbon fixation rests with citryl-CoA synthase and citryl-CoA lyase genes found in the Aquificae consensus genome, and the Thermoproteales consensus genome, which also code for *oorABCD*. Values of Δ^13^C for sites 1–5 were previously reported [2], and those data suggested that the rTCA cycle contributed the most to the Δ^13^C signature of site 1 biofilms. Further downstream, at sites 2 and 3, the CO_2_-fixation genes found in the BPEG were dominated by both rTCA and reductive acetyl co-A pathway (rACP) genes. While the rACP gene, CO dehydrogenase, is not unique to this pathway, the bins containing a CO dehydrogenase match are from phyla known to perform this reaction [53]. The Δ^13^C data from the biofilms at these locations, in the range of ∼ −5 to −12‰, support these assignments [28]. In the photosynthetic zone, sites 4 and 5, it might be expected that the majority of CO_2_-fixation genes would belong to the Calvin cycle (reductive pentose phosphate cycle), due to the prevalence of photosynthetic organisms. However, total gene counts in the BPEG indicate that more rTCA genes than Calvin cycle genes are found in these samples, and the rACP processes appear to have a larger impact on the Δ^13^C of the photosynthetic biofilms [2]. In addition, a single set of ribulose-1,5-bisphosphate carboxylase (the key enzyme in the Calvin cycle) genes were identified at site 3 (BPEG3_14745 and BPEG3_14746). This sequence shares 89% amino acid identity with a predicted protein from *Meiothermus silvanus* DSM 9946 (YP_003686579) [57], though Thermales are not known to be autotrophic. Malonate semialdehyde reductase, associated with the 3-hydroxypropionate cycle (3HP) was also found in the Chloroflexi consensus genome from sites 4 and 5, which was expected as this cycle is specific to the Chloroflexi [58].

While DIC concentrations are likely largely dependent on gas produced in the hydrothermal system, DOC may be directly affected by meadow input. In the chemosynthetic sites 1 and 2 the dominant organisms –are known to use primarily chemoheterotrophic and chemoautotrophic metabolisms in pure cultures. Site 3, however, is dominated by an organism from a putatively novel phylum. This organism, (which, based on the consensus genome appears to be an aerobic heterotroph) would be in direct competition with not only other heterotrophs, but also chemolithotrophs related to those upstream and phototrophs related to those downstream. In fact, the apparent lack of any phototrophs at site 3 in BP (which appears within the suggested bounds of phototrophy [50]), could be a consequence of this competition.

Comparison of nucleotide sequences from this novel phylum with earlier YNP environmental genomes has revealed putative relatives at similar temperatures (60–65°C) at Mushroom and Octopus Springs [10]. While 23% of the reciprocal nucleotide BLAST matches were assigned to an aerobic chemoorganoheterotrophic cluster (Cluster-7), the majority of matches (73.5%) were unassigned at a higher average percent identity than the Cluster-7 matches (94.9% vs 93.4% for Cluster-7). Though this cluster was not a large component of the Mushroom/Octopus environmental genome (<10% of assigned sequences), the novel BPEG phylum matches nearly 70% of Mushroom/Octopus Cluster-7 sequences. Similar analysis to another YNP environmental genome [9] revealed almost no matches, though this was expected, as the Inskeep et al. study sampled no sites with similar temperature or pH. Further environmental sequencing will hopefully reveal the extent to which this novel phylum is represented in hydrothermal systems and how its particular metabolism shapes the community around it.

### Conclusion

Here we present large-scale community sequencing from five sites along an environmental gradient. The “Bison Pool” outflow channel follows several broad changes in key environmental factors, including changes in redox, major ion, and trace element chemistry. These changes in environmental conditions accompany shifts in community structure and metabolic capacity. The hot, strictly chemotrophic sites 1 and 2 are dominated by Aquificae, and a Thermoproteales relative whose consensus genomes appear to have the capacity for S-oxidation and SO_4_^−2^-reduction, respectively. Each of these organisms has the genetic capacity for both autotrophic and heterotrophic growth. Furthermore, there is evidence that the Aquificae, Thermoproteales, and Deinococcus-Thermus at sites 1, 2 and 3 can utilize NO_3_^−^ as an oxidant for anaerobic growth. Though dominant only at the transition site 3, members of Deinococcus-Thermus and the novel Actinobacteria-Planctomycetes phylum continue their heterotrophic lifestyle within the phototrophic communities at sites 4 & 5. Site 4 & 5 phototroph-dominated communities rapidly change their local environment by replenishing nitrogen levels (through N_2_-fixation) and enriching their immediate surroundings with oxygen (through oxygenic photosynthesis). BP exhibits an incomplete nitrogen cycle, apparently only supporting partial capacity for denitrification throughout the outflow, and N_2_-fixation only in the photosynthetic zones.

This work illustrates the power of a long, Sanger-read metagenome library in generating near-complete microbial genomes capable of revealing key secrets to their metabolic lifestyle. At this depth of coverage, we were able to assess not only metabolic capability of individual organisms and shifts in community composition, but also ecotype specialization between adjacent communities. In an ever-growing sea of DNA sequence data from both natural environments and single genomes, we were able to use the combined strengths of genomic and geochemical datasets to model sulfur, nitrogen, and carbon biogeochemical cycles between 5 geographically and physically related, but distinct, sample locations within the same hot spring ecosystem.

## Supporting Information

Figure S1 **Images of sampling area.** [a] “Bison Pool”, source pool and upper 1 m of outflow. View is roughly westerly. [b] Area of outflow near site 2, in the chemosynthetic zone. The three arrows point to tufts of steamer biofilms *in situ.* [c] Close up image of streamer biofilms near site 2. Arrows point to trapped organic material such as soil, bison excrement, or insects. Note range of pigmentation in streamer biofilms in photos ‘b’ and ‘c’. [d] Beginning of outflow channel, showing mineralized edges and bottom of channel bed, the narrow width of the channel bed, and the rapid fluid flow through this part of the outflow. Temperature range  = 74–86°C. [e] View of first two sampling sites, showing clearly the change in the nature of the outflow channel from very narrow and mineralized with rapid fluid flow, to wide and shallow with slower flow. [f] View downstream showing sites 2–4. Sites 3 and 4 are in the less mineralized portion of the outflow, where ready exchange with meadow materials occurs regularly. [g] Sites 2–5. Lower portion of the outflow channel has copious exchange with meadow material, shown also in photo ‘h’. [i] Close up view of hard, mineralized edge of main source pool. This mineralization provides a “wall” to the edge of the upper, chemosynthetic reaches of the outflow. This changing nature of the outflow is also seen in photo ‘j’. [j] An “aerial” view of site 2, taken from ∼15 ft in the air. Flow is from right to left. The dashed line shows an approximate boundary between “walled” outflow upstream, and outflow with a “softer” edge downstream. The sharp, distinct white colored sinter edge is evident in the upstream section, while the downstream section has a less distinct and transient edge. Also shown is an example of meadow fluids (within the drawn circle) collected in the hoof prints of bison. Photo was taken on a dry, sunny day – moisture is the result of condensation from steam, and is a typical condition surrounding the BP outflow. This fluid mixes easily with the “softer” edged lower outflow, but only during large precipitation events does the upper outflow receive large-scale meadow input. [k] “Aerial” photo (∼15 ft up) taken in 2011, at the west end of the source pool following a ∼1 hour heavy rain event. Within the drawn circle, collected rain has mixed with soil and bison excrement, which entered the source pool. This material can be easily seen in the source pool in photo ‘l’, which is brown in this photo. Compare to the “typical” color of BP in photo ‘m’.
(TIF)

Figure S2 **Maximum Likelihood (ML) phylogenetic reconstruction of BP 16S rRNA clone data.** BP 16S rRNA sequences were grouped into OTUs using a nearest-neighbor 92% identity minimum cutoff; branch width indicates the number of sequences in an OTU. Branch color indicates sample location: red - site 1; orange - site 2; magenta - site 3; blue - site 4; and green - site 5. ML was run 10 times using randomized input order.
(TIF)

Figure S3 **Principal Component Analysis (PCA) plot of BPEG tetranucleotides from site 3.** Unassigned sequences are shown as black dots. Sequences assigned using homology and tetranucleotide binning are colored: blue - Aquificae; yellow - Deinococcus-Thermus; and red Actinobacteria-Planctomycetes. Image is a 2D projection of a 3D plot using the first 3 PCA dimensions (bin assignment used all 10 dimensions).
(TIF)

Table S1 **Geochemical data measurements from BP. Data from 2005 unless otherwise indicated.**
(XLS)

Table S2 **Diversity and Evenness measurements for BP sequence data.**
(DOC)

Table S3 **Predicted energy availability based on slope/intercept pairs as reported in Shock et al., 2010.** Reactions sorted by oxidants. Reaction numbers and reactions are the same as in Table 5 of Shock et al., 2010. Numbers in brackets indicate the number of electrons transferred in each reaction. Reactions in italics indicate that no energy is available for the reaction as written at any site. Reactions in bold type indicate processes that pass from positive values of Affinity to negative values (or vice versa) down the outflow.
(DOC)

Table S4 **Reactions for each site ranked in order from highest to lowest Affinity values.** Ranking number is given in far left column. Reaction numbers correspond to those in Table S3.
(DOC)

Table S5 **Counts of functional genes found in consensus genomes (non-normalized to the size of the datasets).**
(DOC)

## References

[1] Dick JM, Shock EL (2011). Calculation of the relative chemical stabilities of proteins as a function of temperature and redox chemistry in a hot spring.. PLoS One.

[2] Havig JR, Raymond J, Meyer-Dombard DR, Zolotova N, Shock EL (2011). Merging isotopes and community genomics in a siliceous sinter-depositing hot spring.. J Geophys Res.

[3] Meyer-Dombard DR, Shock EL, Amend JP (2005). Archaeal and bacterial communities in geochemically diverse hot springs of Yellowstone National Park, USA.. Geobiol.

[4] Meyer-Dombard DR, Swingley W, Raymond J, Havig J, Shock EL (2011). Hydrothermal ecotones and streamer biofilm communities in the Lower Geyser Basin, Yellowstone National Park.. Environ Microbiol.

[5] Chivian D, Brodie EL, Alm EJ, Culley DE, Dehal PS (2008). Environmental genomics reveals a single-species ecosystem deep within Earth.. Science.

[6] Dick GJ, Andersson AF, Baker BJ, Simmons SL, Thomas BC (2009). Community-wide analysis of microbial genome sequence signatures.. Genome Biol.

[7] Tyson GW, Chapman J, Hugenholtz P, Allen EE, Ram RJ (2004). Community structure and metabolism through reconstruction of microbial genomes from the environment.. Nature.

[8] Breitbart M, Salamon P, Andresen B, Mahaffy JM, Segall AM (2002). Genomic analysis of uncultured marine viral communities.. Proc Natl Acad Sci U S A.

[9] Inskeep WP, Rusch DB, Jay ZJ, Herrgard MJ, Kozubal MA (2010). Metagenomes from high-temperature chemotrophic systems reveal geochemical controls on microbial community structure and function.. PLoS One.

[10] Klatt CG, Wood JM, Rusch DB, Bateson MM, Hamamura N (2011). Community ecology of hot spring cyanobacterial mats: predominant populations and their functional potential.. ISME J.

[11] Rusch DB, Halpern AL, Sutton G, Heidelberg KB, Williamson S (2007). The *Sorcerer II* Global Ocean Sampling Expedition: Northwest Atlantic through Eastern Tropical Pacific.. PLoS Biol.

[12] Venter JC, Remington K, Heidelberg JF, Halpern AL, Rusch D (2004). Environmental genome shotgun sequencing of the Sargasso Sea.. Science.

[13] DeLong EF (2005). Microbial community genomics in the ocean.. Nat Rev Microbiol.

[14] DeLong EF, Preston CM, Mincer T, Rich V, Hallam SJ (2006). Community genomics among stratified microbial assemblages in the ocean’s interior.. Science.

[15] Haque MM, Ghosh TS, Komanduri D, Mande SS (2009). SOrt-ITEMS: Sequence orthology based approach for improved taxonomic estimation of metagenomic sequences.. Bioinformatics.

[16] Huson DH, Auch AF, Qi J, Schuster SC (2007). MEGAN analysis of metagenomic data.. Genome Res.

[17] Kislyuk A, Bhatnagar S, Dushoff J, Weitz J (2009). Unsupervised statistical clustering of environmental shotgun sequences.. BMC Bioinformatics.

[18] Krause L, Diaz NN, Goesmann A, Kelley S, Nattkemper TW (2008). Phylogenetic classification of short environmental DNA fragments..

[19] Sandberg R, Winberg G, Bränden C-I, Kaske A, Ernberg I (2001). Capturing whole-genome characteristics in short sequences using a naïve Bayesian classifier.. Genome Res.

[20] Teeling H, Meyerdierks A, Bauer M, Amann R, Glöckner FO (2004). Application of tetranucleotide frequencies for the assignment of genomic fragments.. Environ Microbiol.

[21] Teeling H, Waldmann J, Lombardot T, Bauer M, Glockner FO (2004). TETRA: a web-service and a stand-alone program for the analysis and comparison of tetranucleotide usage patterns in DNA sequences.. BMC Bioinformatics.

[22] McHardy AC, Martin HG, Tsirigos A, Hugenholtz P, Rigoutsos I (2007). Accurate phylogenetic classification of variable-length DNA fragments.. Nat Meth.

[23] McHardy AC, Rigoutsos I (2007). What’s in the mix: phylogenetic classification of metagenome sequence samples.. Curr Opin Microbiol.

[24] Kunin V, Copeland A, Lapidus A, Mavromatis K, Hugenholtz P (2008). A bioinformatician’s guide to metagenomics.. Microbiol Mol Biol Rev.

[25] Eisen JA (2007). Environmental shotgun sequencing: its potential and challenges for studying the hidden world of microbes.. PLoS Biol.

[26] Altschul SF, Gish W, Miller W, Myers EW, Lipman DJ (1990). Basic Local Alignment Search Tool.. J Mol Biol.

[27] Nakashima H, Ota M, Nishikawa K, Ooi T (1998). Genes from nine genomes are separated into their organisms in the dinucleotide composition space.. DNA Res.

[28] Abe T, Kanaya S, Kinouchi M, Ichiba Y, Kozuki T (2003). Informatics for unveiling hidden genome signatures.. Genome Res.

[29] Meyer-Dombard DR, Shock EL, Amend JP (2012). Effects of trace element concentrations on culturing thermophiles.. Extremophiles.

[30] Shock EL, Holland M, Meyer-Dombard D, Amend JP, Osburn GR (2010). Quantifying inorganic sources of geochemical energy in hydrothermal ecosystems, Yellowstone National Park, USA.. Geochim Cosmochim Acta.

[31] Cole JR, Wang Q, Cardenas E, Fish J, Chai B (2009). The Ribosomal Database Project: improved alignments and new tools for rRNA analysis.. Nucleic Acids Res.

[32] Larkin MA, Blackshields G, Brown NP, Chenna R, McGettigan PA (2007). Clustal W and Clustal X version 2.0.. Bioinformatics.

[33] Schloss PD, Handelsman J (2005). Introducing DOTUR, a computer program for defining operational taxonomic units and estimating species richness.. Appl Environ Microbiol.

[34] Felsenstein J (1989). PHYLIP–Phylogenetics Inference Package (Version 3.2).. Cladistics.

[35] Shannon CE (1948). A mathematical theory of communication.. Bell Syst Tech J 27: 379–423,.

[36] Simpson EH (1949). Measurement of Diversity.. Nature.

[37] Pielou EC (1966). The measurement of diversity in different types of biological collections.. J Theor Biol.

[38] Kanehisa M, Araki M, Goto S, Hattori M, Hirakawa M (2008). KEGG for linking genomes to life and the environment.. Nucleic Acids Res.

[39] Van Dongen S (2000). Graph clustering by flow simulation..

[40] Ren Y, Ren Y, Zhou Z, Guo X, Li Y (2010). Complete genome sequence of *Enterobacter cloacae* subsp. cloacae type strain ATCC 13047.. J Bacteriol.

[41] Arai H, Kanbe H, Ishii M, Igarashi Y (2010). Complete genome sequence of the thermophilic, obligately chemolithoautotrophic hydrogen-oxidizing bacterium *Hydrogenobacter thermophilus* TK-6.. J Bacteriol.

[42] Wirth R, Sikorski J, Brambilla E, Misra M, Lapidus A (2010). Complete genome sequence of *Thermocrinis albus* type strain (HI 11/12(T)).. Standards in Genomic Sciences.

[43] Deckert G, Warren PV, Gaasterland T, Young WG, Lenox AL (1998). The complete genome of the hyperthermophilic bacterium *Aquifex aeolicus*.. Nature.

[44] Hall JR, Mitchell KR, Jackson-Weaver O, Kooser AS, Cron BR (2008). Molecular characterization of the diversity and distribution of a thermal spring microbial community by using rRNA and metabolic genes.. Appl Environ Microbiol.

[45] Wu D, Raymond J, Wu M, Chatterji S, Ren Q (2009). Complete genome sequence of the aerobic CO-oxidizing thermophile *Thermomicrobium roseum*.. PLoS One.

[46] Hugenholtz P, Stackebrandt E (2004). Reclassification of *Sphaerobacter thermophilus* from the subclass *Sphaerobacteridae* in the phylum *Actinobacteria* to the class *Thermomicrobia* (emended description) in the phylum *Chloroflexi* (emended description).. Int J Syst Evol Microbiol.

[47] Tang K-H, Barry K, Chertkov O, Dalin E, Han CS (2011). Complete genome sequence of the filamentous anoxygenic phototrophic bacterium *Chloroflexus aurantiacus*.. BMC Genomics.

[48] Bhaya D, Grossman AR, Steunou A-S, Khuri N, Cohan FM (2007). Population level functional diversity in a microbial community revealed by comparative genomic and metagenomic analyses.. ISME Journal.

[49] Windman T, Zolotova N, Schwandner F, Shock EL (2007). Formate as an energy source for microbial metabolism in chemosynthetic zones of hydrothermal ecosystems.. Astrobiology.

[50] Cox A, Shock EL, Havig JR (2011). The transition to microbial photosynthesis in hot spring ecosystems.. Chem Geol.

[51] D’Imperio S, Lehr CR, Oduro H, Druschel G, Kuhl M (2008). Relative importance of H_2_ and H_2_S as energy sources for primary production in geothermal springs.. Appl Environ Microbiol.

[52] Ghosh W, Dam B (2009). Biochemistry and molecular biology of lithotrophic sulfur oxidation by taxonomically and ecologically diverse bacteria and archaea.. FEMS Microbiol Rev.

[53] Boone DR, Castenholz RW (2001). Bergey’s Manual of Systematic Bacteriology - Volume One:The *Archaea* and the Deeply Branching and Phototrophic *Bacteria*..

[54] Hedlund BP, McDonald AI, Lam J, Dodsworth JA, Brown JR (2011). Potential role of *Thermus thermophilus* and *T. oshimai* in high rates of nitrous oxide (N_2_O) production in ∼80°C hot springs in the US Great Basin.. Geobiol.

[55] de la Torre JR, Walker CB, Ingalls AE, Könneke M, Stahl DA (2008). Cultivation of a thermophilic ammonia oxidizing archaeon synthesizing crenarchaeol.. Environ Microbiol.

[56] Zhang CL, Ye Q, Huang Z, Li W, Chen J (2008). Global occurrence of archaeal *amoA* genes in terrestrial hot springs.. Appl Environ Microbiol.

[57] Sikorski J, Tindall BJ, Lowry S, Lucas S, Nolan M (2010). Complete genome sequence of *Meiothermus silvanus* type strain (VI-R2(T)).. Standards in Genomic Sciences.

[58] Berg IA, Kockelkorn D, Ramos-Vera WH, Say RF, Zarzycki J (2010). Autotrophic carbon fixation in archaea.. Nat Rev Microbiol.

