# Supplementary figures and images for: Coordinating Environmental Genomics and Geochemistry Reveals Metabolic Transitions in a Hot Spring Ecosystem
Source: PLoS One. 2012 Jun 4;7(6):e38108. doi: 10.1371/journal.pone.0038108 (PMC3367023; doi:10.1371/journal.pone.0038108)

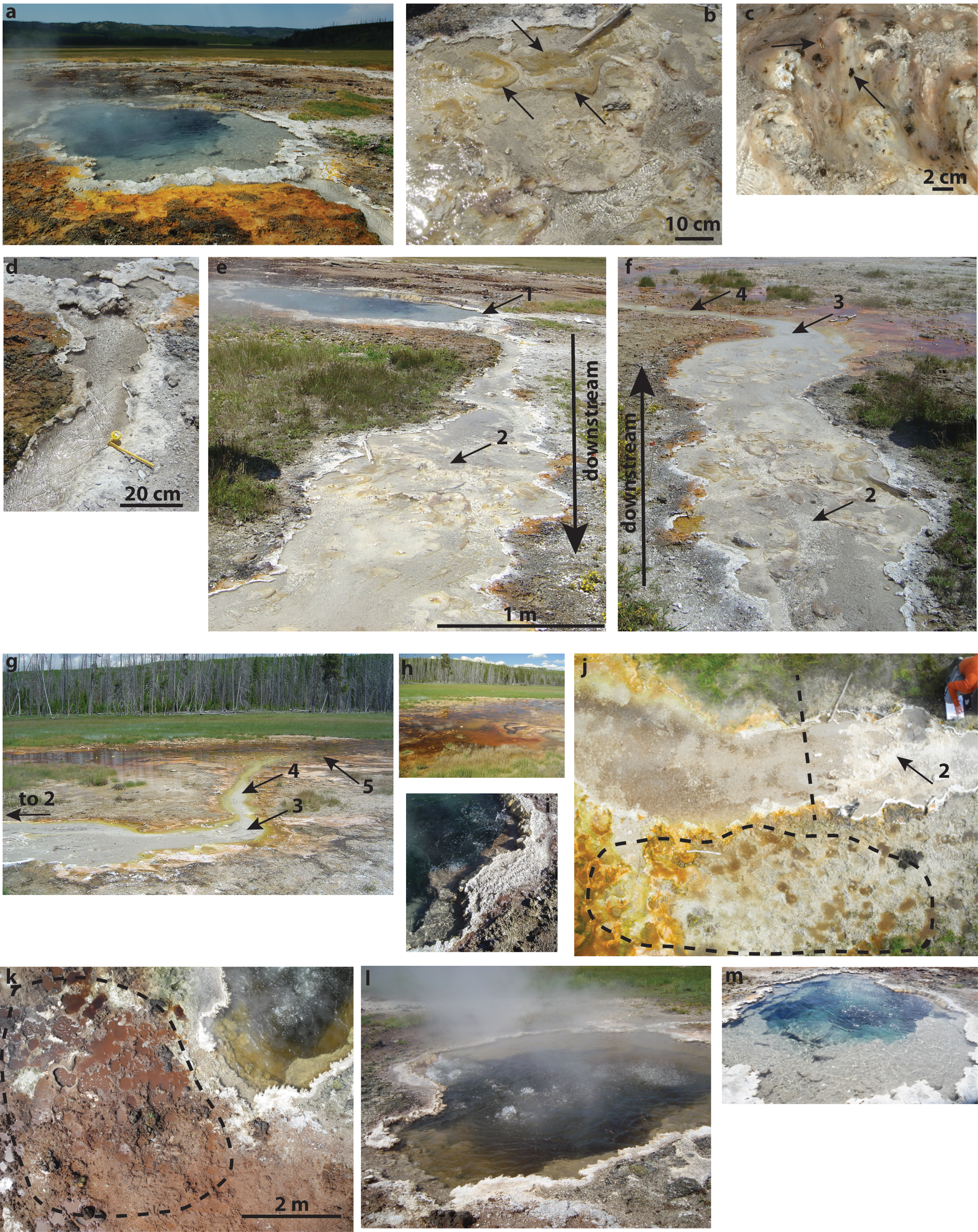

Supplement: Figure S1 — Images of sampling area. [a] “Bison Pool”, source pool and upper 1 m of outflow. View is roughly westerly. [b] Area of outflow near site 2, in the chemosynthetic zone. The three arrows point to tufts of steamer biofilms in situ. [c] Close up image of streamer biofilms near site 2. Arrows point to trapped organic material such as soil, bison excrement, or insects. Note range of pigmentation in streamer biofilms in photos ‘b’ and ‘c’. [d] Beginning of outflow channel, showing mineralized edges and bottom of channel bed, the narrow width of the channel bed, and the rapid fluid flow through this part of the outflow. Temperature range = 74–86°C. [e] View of first two sampling sites, showing clearly the change in the nature of the outflow channel from very narrow and mineralized with rapid fluid flow, to wide and shallow with slower flow. [f] View downstream showing sites 2–4. Sites 3 and 4 are in the less mineralized portion of the outflow, where ready exchange with meadow materials occurs regularly. [g] Sites 2–5. Lower portion of the outflow channel has copious exchange with meadow material, shown also in photo ‘h’. [i] Close up view of hard, mineralized edge of main source pool. This mineralization provides a “wall” to the edge of the upper, chemosynthetic reaches of the outflow. This changing nature of the outflow is also seen in photo ‘j’. [j] An “aerial” view of site 2, taken from ∼15 ft in the air. Flow is from right to left. The dashed line shows an approximate boundary between “walled” outflow upstream, and outflow with a “softer” edge downstream. The sharp, distinct white colored sinter edge is evident in the upstream section, while the downstream section has a less distinct and transient edge. Also shown is an example of meadow fluids (within the drawn circle) collected in the hoof prints of bison. Photo was taken on a dry, sunny day – moisture is the result of condensation from steam, and is a typical condition surrounding the BP outflow. This fluid mixes eas [file pone.0038108.s001.tif]

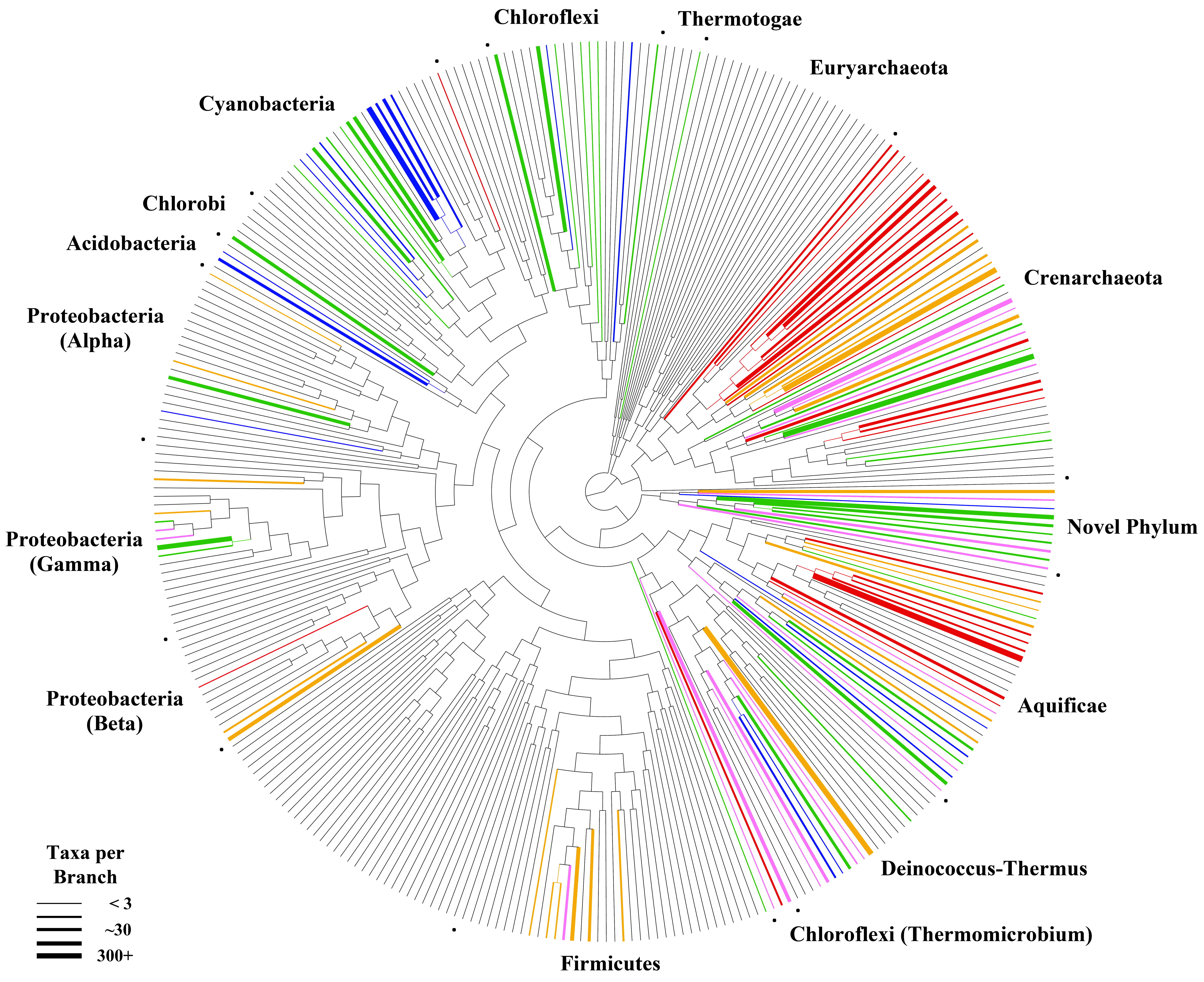

Supplement: Figure S2 — Maximum Likelihood (ML) phylogenetic reconstruction of BP 16S rRNA clone data. BP 16S rRNA sequences were grouped into OTUs using a nearest-neighbor 92% identity minimum cutoff; branch width indicates the number of sequences in an OTU. Branch color indicates sample location: red - site 1; orange - site 2; magenta - site 3; blue - site 4; and green - site 5. ML was run 10 times using randomized input order. (TIF) [file pone.0038108.s002.tif]

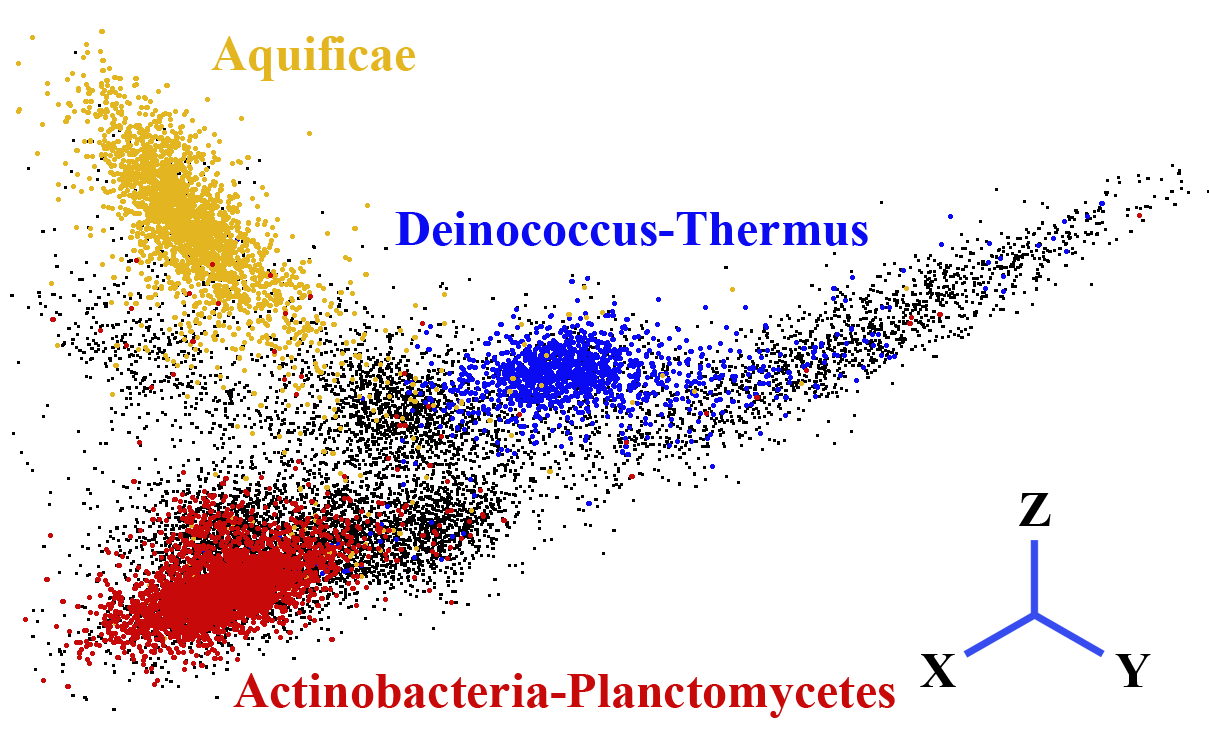

Supplement: Figure S3 — Principal Component Analysis (PCA) plot of BPEG tetranucleotides from site 3. Unassigned sequences are shown as black dots. Sequences assigned using homology and tetranucleotide binning are colored: blue - Aquificae; yellow - Deinococcus-Thermus; and red Actinobacteria-Planctomycetes. Image is a 2D projection of a 3D plot using the first 3 PCA dimensions (bin assignment used all 10 dimensions). (TIF) [file pone.0038108.s003.tif]
