# Supplementary material for: Coordinating Environmental Genomics and Geochemistry Reveals Metabolic Transitions in a Hot Spring Ecosystem
Source: PLoS One. 2012 Jun 4;7(6):e38108. doi: 10.1371/journal.pone.0038108 (PMC3367023; doi:10.1371/journal.pone.0038108)
Supplement: Table S2 — Diversity and Evenness measurements for BP sequence data. (DOC) [file pone.0038108.s005.doc]

Supplemental Table 2. Diversity and Evenness measurements for BP sequence data.

|  |  | Shannon Index |  |  |  | Simpson Index |  |  |  | Pielou Index |  |
| --- | --- | --- | --- | --- | --- | --- | --- | --- | --- | --- | --- |
|  | Clone 16S | BPEG 16S | Reads |  | Clone 16S | BPEG 16S | Reads |  | Clone 16S | BPEG 16S | Reads |
| Site 1 | 0.783 | 1.058 | 0.854 |  | 0.440 | 0.457 | 0.559 |  | 0.377 | 0.509 | 0.411 |
| Site 2 | 1.335 | 1.303 | 1.436 |  | 0.313 | 0.326 | 0.331 |  | 0.537 | 0.524 | 0.578 |
| Site 3 | 0.861 | 1.346 | 1.405 |  | 0.395 | 0.327 | 0.334 |  | 0.359 | 0.561 | 0.586 |
| Site 4 | 0.716 | 1.461 | 1.845 |  | 0.692 | 0.276 | 0.224 |  | 0.253 | 0.516 | 0.651 |
| Site 5 | 1.578 | 1.721 | 1.829 |  | 0.229 | 0.207 | 0.241 |  | 0.527 | 0.574 | 0.611 |
