# Supplementary material for: Coordinating Environmental Genomics and Geochemistry Reveals Metabolic Transitions in a Hot Spring Ecosystem
Source: PLoS One. 2012 Jun 4;7(6):e38108. doi: 10.1371/journal.pone.0038108 (PMC3367023; doi:10.1371/journal.pone.0038108)
Supplement: Table S3 — Predicted energy availability based on slope/intercept pairs as reported in Shock et al., 2010. Reactions sorted by oxidants. Reaction numbers and reactions are the same as in Table 5 of Shock et al., 2010. Numbers in brackets indicate the number of electrons transferred in each reaction. Reactions in italics indicate that no energy is available for the reaction as written at any site. Reactions in bold type indicate processes that pass from positive values of Affinity to negative values (or vice versa) down the outflow. (DOC) [file pone.0038108.s006.doc]

**Supplemental Table 3.** Predicted energy availability based on slope/intercept pairs as reported in Shock et al., 2010. Reactions sorted by oxidants. Reaction numbers and reactions are the same as in Table 5 of Shock et al., 2010. Numbers in brackets indicate the number of electrons transferred in each reaction. Reactions in italics indicate that no energy is available for the reaction as written at any site. Reactions in bold type indicate processes that pass from positive values of Affinity to negative values (or vice versa) down the outflow.

| **Reaction #** | **Reactions** | **oxidant** | **reductant** | **intercept** | **slope** | **Affinity, site 1 [kcal/mol e-]** | **Affinity, site2 [kcal/mol e-]** | **Affinity, site 3 [kcal/mol e-]** | **Affinity, site 4 [kcal/mol e-]** | **Affinity, site 5 [kcal/mol e-]** |
| --- | --- | --- | --- | --- | --- | --- | --- | --- | --- | --- |
| 1 | 2H_2_(g) + O_2_(aq) --> 2H_2_O [4] | O2 | H2 | 24770 | -150 | 23.67 | 23.62 | 23.58 | 23.57 | 23.53 |
| 2 | NH_4_^+^ + (3/2)O_2_(aq) --> NO_2_^-^ + 2H^+^ + H_2_O [6] | O2 | NH4+ | 7410 | 430 | 10.57 | 10.71 | 10.82 | 10.85 | 10.96 |
| 3 | NH_4_^+^ + 2O_2_(aq) --> NO_3_^-^ + 2H^+^ + H_2_O [8] | O2 | NH4+ | 7440 | 320 | 9.79 | 9.90 | 9.98 | 10.00 | 10.08 |
| 4 | NO_2_^-^ + (1/2)O_2_(aq) --> NO_3_^-^ [2] | O2 | NO2- | 7690 | -30 | 7.47 | 7.46 | 7.45 | 7.45 | 7.44 |
| 5 | 2H_2_S(aq) + Fe^+2^ + (1/2)O_2_(aq) --> PYRITE + 2H^+^ + H_2_O [2] | O2 | H2S | 17540 | 980 | 24.74 | 25.06 | 25.31 | 25.38 | 25.63 |
| 6 | H_2_S(aq) + (1/2)O_2_(aq) --> SULFUR + H_2_O [2] | O2 | H2S | 20150 | -260 | 18.24 | 18.15 | 18.09 | 18.07 | 18.00 |
| 7 | H_2_S(aq) + 2O_2_(aq) --> SO_4_^-2^ + 2H^+^ [8] | O2 | H2S | 19590 | 375 | 22.35 | 22.47 | 22.56 | 22.59 | 22.69 |
| 8 | PYRITE + 2H^+^ + (1/2)O_2_(aq) --> 2SULFUR + Fe^+2^ + H_2_O [2] | O2 | Pyrite | 21570 | -1210 | 12.68 | 12.28 | 11.97 | 11.90 | 11.58 |
| 9 | PYRITE + (7/2)O_2_(aq) + H_2_O --> 2SO_4_^-2^ + Fe^+2^ + 2H^+^ [14] | O2 | Pyrite | 19680 | 340 | 22.18 | 22.29 | 22.38 | 22.40 | 22.49 |
| 10 | SULFUR + (3/2)O_2_(aq)+ H_2_O --> SO_4_^-2^ + 2H^+^ [6] | O2 | S | 19400 | 590 | 23.74 | 23.93 | 24.08 | 24.12 | 24.27 |
| 11 | 2Fe^+2^ + (1/2)O_2_(aq) + 2H_2_O --> HEMATITE + 4H^+^ [2] | O2 | Fe+2 | 4570 | 2490 | 22.87 | 23.69 | 24.32 | 24.48 | 25.13 |
| 12 | 3Fe^+2^ + (1/2)O_2_(aq) + 3H_2_O --> MAGNETITE + 6H^+^ [2] | O2 | Fe+2 | -4770 | 3720 | 22.57 | 23.79 | 24.74 | 24.97 | 25.95 |
| 13 | Fe^+2^ + (1/4)O_2_(aq)+ (3/2)H_2_O --> GOETHITE + 2H^+^ [1] | O2 | Fe+2 | 4670 | 2490 | 22.97 | 23.79 | 24.42 | 24.58 | 25.23 |
| 14 | 2MAGNETITE + (1/2)O_2_(aq) --> 3HEMATITE [2] | O2 | Magnetite | 23400 | -10 | 23.33 | 23.32 | 23.32 | 23.32 | 23.32 |
| 15 | 2MAGNETITE + (1/2)O_2_(aq) + 3H_2_O --> 6GOETHITE [2] | O2 | Magnetite | 23780 | -35 | 23.52 | 23.51 | 23.50 | 23.50 | 23.49 |
| 16 | CH_4_(g) + (3/2)O_2_(aq) --> CO(g) + 2H_2_O [6] | O2 | CH4 | 22000 | 25 | 22.18 | 22.19 | 22.20 | 22.20 | 22.21 |
| 17 | CH_4_(g) + 2O_2_(aq) --> CO_2_(g) + 2H_2_O [8] | O2 | CH4 | 23000 | -40 | 22.71 | 22.69 | 22.68 | 22.68 | 22.67 |
| 18 | CH_4_(g) + 2O_2_(aq) --> HCO_3_^-^ + H^+^ + H_2_O [8] | O2 | CH4 | 23540 | -80 | 22.95 | 22.93 | 22.91 | 22.90 | 22.88 |
| 19 | CO(g) + (1/2)O_2_(aq) --> CO_2_(g) [2] | O2 | CO | 25960 | -260 | 24.05 | 23.96 | 23.90 | 23.88 | 23.81 |
| 20 | H_2_(g) + NO_3_^-^ --> NO_2_^-^ + H_2_O [2] | NO3- | H2 | 17080 | -190 | 15.68 | 15.62 | 15.57 | 15.56 | 15.51 |
| 21 | 2H_2_S(aq) + NO_3_^-^ + Fe^+2^ --> PYRITE + NO_2_^-^ + 2H^+^ + H_2_O [2] | NO3- | H2S | 11130 | 780 | 16.86 | 17.12 | 17.32 | 17.37 | 17.57 |
| 22 | H_2_S(aq) + NO_3_^-^ --> SULFUR + NO_2_^-^ + H_2_O [2] | NO3- | H2S | 12810 | -300 | 10.61 | 10.51 | 10.43 | 10.41 | 10.33 |
| 23 | H_2_S(aq) + 4NO_3_^-^ --> SO_4_^-2^ + 4NO_2_^-^ + 2H^+^ [8] | NO3- | H2S | 11900 | 400 | 14.84 | 14.97 | 15.07 | 15.10 | 15.20 |
| 24 | PYRITE + NO_3_^-^ + 2H^+^ --> 2SULFUR + Fe^+2^ + NO_2_^-^ + H_2_O [2] | NO3- | Pyrite | 13920 | -1210 | 5.03 | 4.63 | 4.32 | 4.25 | 3.93 |
| 25 | PYRITE + 7NO_3_^-^ + H_2_O --> Fe^+2^ + 2SO_4_^-2^ + 7NO_2_^-^ + 2H^+^ [14] | NO3- | Pyrite | 12100 | 340 | 14.60 | 14.71 | 14.80 | 14.82 | 14.91 |
| 26 | SULFUR + 3NO_3_^-^ + H_2_O --> SO_4_^-2^ + 3NO_2_^-^ + 2H^+^ [6] | NO3- | S | 11630 | 620 | 16.19 | 16.39 | 16.55 | 16.59 | 16.75 |
| 27 | 2Fe^+2^ + NO_3_^-^ + 2H_2_O --> HEMATITE + NO_2_^-^ + 4H^+^ [2] | NO3- | Fe+2 | -2730 | 2470 | 15.42 | 16.23 | 16.86 | 17.02 | 17.66 |
| 28 | 3Fe^+2^ + NO_3_^-^ + 3H_2_O --> MAGNETITE + NO_2_^-^ + 6H^+^ [2] | NO3- | Fe+2 | -11950 | 3700 | 15.25 | 16.46 | 17.40 | 17.63 | 18.60 |
| 29 | 2Fe^+2^ + NO_3_^-^ + 3H_2_O --> 2GOETHITE + NO_2_^-^ + 4H^+^ [2] | NO3- | Fe+2 | -2660 | 2470 | 15.49 | 16.30 | 16.93 | 17.09 | 17.73 |
| 30 | 2MAGNETITE + NO_3_^-^ --> 3HEMATITE + NO_2_^-^ [2] | NO3- | Magnetite | 15640 | 20 | 15.79 | 15.79 | 15.80 | 15.80 | 15.81 |
| 31 | 2MAGNETITE + NO_3_^-^ + H_2_O --> 6GOETHITE + NO_2_^-^ [2] | NO3- | Magnetite | 15950 | 0 | 15.95 | 15.95 | 15.95 | 15.95 | 15.95 |
| 32 | CH_4_(g) + 3NO_3_^-^ --> CO(g) + 3NO_2_^-^ + 2H_2_O [6] | NO3- | CH4 | 15160 | 30 | 15.38 | 15.39 | 15.40 | 15.40 | 15.41 |
| 33 | CH_4_(g) + 4NO_3_^-^ --> CO_2_(g) + 4NO_2_^-^ + H_2_O [8] | NO3- | CH4 | 16480 | -180 | 15.16 | 15.10 | 15.05 | 15.04 | 14.99 |
| 34 | CH_4_(g) + 4NO_3_^-^ --> HCO_3_^-^ + 4NO_2_^-^ + H^+^ + H_2_O [8] | NO3- | CH4 | 24410 | -1390 | 14.19 | 13.74 | 13.38 | 13.30 | 12.93 |
| 35 | CO(g) + NO_3_^-^ --> CO_2_(g) + NO_2_^-^ [2] | NO3- | CO | 18640 | -140 | 17.61 | 17.57 | 17.53 | 17.52 | 17.48 |
| *36* | *NO_3_^-^ --> NO_2_^-^ + (1/2)O_2_(aq) [2]* | *NO3-* | *N/A* | *-7690* | *30* | *-7.47* | *-7.46* | *-7.45* | *-7.45* | *-7.44* |
| 37 | NH_4_^+^ + 3NO_3_^-^ --> 2H^+^ + 4NO_2_^-^ + H_2_O [6] | NO3- | NH4+ | 10 | 390 | 2.88 | 3.00 | 3.10 | 3.13 | 3.23 |
| 38 | 4H_2_(g) + NO_3_^-^ + 2H^+^ --> NH_4_^+^ + 3H_2_O [8] | NO3- | H2 | 15640 | -260 | 13.73 | 13.64 | 13.58 | 13.56 | 13.49 |
| 39 | 8H_2_S(aq) + NO_3_^-^ + 4Fe^+2^ --> 4PYRITE + NH_4_^+^ + 6H^+^ + 3H_2_O [8] | NO3- | H2S | 10640 | 570 | 14.83 | 15.02 | 15.16 | 15.20 | 15.35 |
| 40 | 4H_2_S(aq) + NO_3_^-^ + 2H^+^ --> 4SULFUR + NH_4_^+^ + 3H_2_O [8] | NO3- | H2S | 12560 | -560 | 8.44 | 8.26 | 8.12 | 8.08 | 7.94 |
| 41 | H_2_S(aq) + NO_3_^-^ + H_2_O --> SO_4_^-2^ + NH_4_^+^ [8] | NO3- | H2S | 11970 | 80 | 12.56 | 12.58 | 12.60 | 12.61 | 12.63 |
| 42 | 4PYRITE + NO_3_^-^ + 10H^+^ --> 8SULFUR + 4Fe^+2^ + NH_4_^+^ + 3H_2_O [8] | NO3- | Pyrite | 13920 | -1520 | 2.75 | 2.25 | 1.86 | 1.77 | 1.37 |
| 43 | 4PYRITE + 7NO_3_^-^ + 6H^+^ + 11H_2_O --> 4Fe^+2^ + 8SO_4_^-2^ + 7NH_4_^+^ [56] | NO3- | Pyrite | 12100 | 40 | 12.39 | 12.41 | 12.42 | 12.42 | 12.43 |
| 44 | 4SULFUR + 3NO_3_^-^ + 7H_2_O --> 4SO_4_^-2^ + 3NH_4_^+^ + 2H^+^ [24] | NO3- | S | 11790 | 300 | 14.00 | 14.09 | 14.17 | 14.19 | 14.27 |
| 45 | 8Fe^+2^ + NO_3_^-^ + 9H_2_O --> 4HEMATITE + NH_4_^+^ + 14H^+^ [8] | NO3- | Fe+2 | -2700 | 2180 | 13.32 | 14.04 | 14.59 | 14.73 | 15.30 |
| 46 | 12Fe^+2^ + NO_3_^-^ + 13H_2_O --> 4MAGNETITE + NH_4_^+^ + 22H^+^ [8] | NO3- | Fe+2 | -11900 | 3410 | 13.16 | 14.28 | 15.15 | 15.36 | 16.26 |
| 47 | 8Fe^+2^ + NO_3_^-^ + 13H_2_O --> 8GOETHITE + NH_4_^+^ + 14H^+^ [8] | NO3- | Fe+2 | -2640 | 2180 | 13.38 | 14.10 | 14.65 | 14.79 | 15.36 |
| 48 | 8MAGNETITE + NO_3_^-^ + 2H^+^ + H_2_O --> 12HEMATITE + NH_4_^+^ [8] | NO3- | Magnetite | 15720 | -300 | 13.52 | 13.42 | 13.34 | 13.32 | 13.24 |
| 49 | 8MAGNETITE + NO_3_^-^ + 2H^+^ + 13H_2_O --> 24GOETHITE + NH_4_^+^ [8] | NO3- | Magnetite | 15970 | -310 | 13.69 | 13.59 | 13.51 | 13.49 | 13.41 |
| 50 | 4CH_4_(g) + 3NO_3_^-^ + 6H^+^ --> 4CO(g) + 3NH_4_^+^ + 5H_2_O [24] | NO3- | CH4 | 14200 | -220 | 12.58 | 12.51 | 12.45 | 12.44 | 12.38 |
| 51 | CH_4_(g) + NO_3_^-^ + 2H^+^ --> CO_2_(g) + NH_4_^+^ + H_2_O [8] | NO3- | CH4 | 15180 | -310 | 12.90 | 12.80 | 12.72 | 12.70 | 12.62 |
| 52 | CH_4_(g) + NO_3_^-^ + H^+^ --> HCO_3_^-^ + NH_4_^+^ [8] | NO3- | CH4 | 17015 | -555 | 12.94 | 12.75 | 12.61 | 12.58 | 12.43 |
| 53 | 4CO(g) + NO_3_^-^ + 2H^+^ + H_2_O --> 4CO_2_(g) + NH_4_^+^ [8] | NO3- | CO | 17670 | -390 | 14.80 | 14.68 | 14.58 | 14.55 | 14.45 |
| *54* | *H_2_O + NO_3_^-^ + 2H^+^ --> NH_4_^+^ + 2O_2_(aq) [8]* | *NO3-* | *N/At* | *-7440* | *-320* | *-9.79* | *-9.90* | *-9.98* | *-10.00* | *-10.08* |
| 55 | 3H_2_(g) + NO_2_^-^ + 2H^+^ --> NH_4_^+^ + 2H_2_O [6] | NO2- | H2 | 15230 | -250 | 13.39 | 13.31 | 13.25 | 13.23 | 13.17 |
| 56 | 6H_2_S(aq) + NO_2_^-^ + 3Fe^+2^ --> 3PYRITE + NH_4_^+^ + 4H^+^ + 2H_2_O [6] | NO2- | H2S | 10400 | 510 | 14.15 | 14.32 | 14.45 | 14.48 | 14.61 |
| 57 | 3H_2_S(aq) + NO_2_^-^ + 2H^+^ --> 3SULFUR + NH_4_^+^ + 2H_2_O [6] | NO2- | H2S | 12470 | -620 | 7.91 | 7.71 | 7.55 | 7.51 | 7.35 |
| 58 | 3H_2_S(aq) + 4NO_2_^-^ + 2H^+^ + 4H_2_O --> 3SO_4_^-2^ + 4NH_4_^+^ [24] | NO2- | H2S | 12040 | -30 | 11.82 | 11.81 | 11.80 | 11.80 | 11.79 |
| **59** | **3PYRITE + NO_2_^-^ + 8H^+^ --> 6SULFUR + 3Fe^+2^ + NH_4_^+^ + 2H_2_O [6]** | **NO2-** | **Pyrite** | **14030** | **-1630** | **2.05** | **1.51** | **1.10** | **1.00** | **0.57** |
| 60 | 3PYRITE + 7NO_2_^-^ + 8H^+^ + 10H_2_O --> 3Fe^+2^ + 6SO_4_^-2^ + | NO2- | Pyrite | 12240 | -100 | 11.51 | 11.47 | 11.45 | 11.44 | 11.41 |
| 61 | SULFUR + NO_2_^-^ + 2H_2_O --> SO_4_^-2^ + NH_4_^+^ [6] | NO2- | S | 11880 | 175 | 13.17 | 13.22 | 13.27 | 13.28 | 13.32 |
| 62 | 6Fe^+2^ + NO_2_^-^ + 7H_2_O --> 3HEMATITE + NH_4_^+^ + 10H^+^ [6] | NO2- | Fe+2 | -2615 | 2025 | 12.27 | 12.93 | 13.45 | 13.57 | 14.11 |
| 63 | 9Fe^+2^ + NO_2_^-^ + 10H_2_O --> 3MAGNETITE + NH_4_^+^ + 16H^+^ [6] | NO2- | Fe+2 | -11830 | 3250 | 12.06 | 13.12 | 13.95 | 14.15 | 15.01 |
| 64 | 6Fe^+2^ + NO_2_^-^ + 10H_2_O --> 6GOETHITE + NH_4_^+^ + 10H^+^ [6] | NO2- | Fe+2 | -2530 | 2025 | 12.35 | 13.02 | 13.53 | 13.66 | 14.19 |
| 65 | 6MAGNETITE + NO_2_^-^ + 2H^+^ + H_2_O --> 9HEMATITE + NH_4_^+^ [6] | NO2- | Magnetite | 15820 | -420 | 12.73 | 12.60 | 12.49 | 12.46 | 12.35 |
| 66 | 6MAGNETITE + NO_2_^-^ + 2H^+^ + 10H_2_O --> 18GOETHITE + NH_4_^+^ [6] | NO2- | Magnetite | 16120 | -430 | 12.96 | 12.82 | 12.71 | 12.68 | 12.57 |
| 67 | CH_4_(g) + NO_2_^-^ + 2H^+^ --> CO(g) + NH_4_^+^ + H_2_O [6] | NO2- | CH4 | 13950 | -330 | 11.52 | 11.42 | 11.33 | 11.31 | 11.23 |
| 68 | 3CH_4_(g) + 4NO_2_^-^ + 8H^+^ --> 3CO_2_(g) + 4NH_4_^+^ + 2H_2_O [24] | NO2- | CH4 | 14880 | -365 | 12.20 | 12.08 | 11.98 | 11.96 | 11.87 |
| 69 | 3CH_4_(g) + 4NO_2_^-^ + 5H^+^ + H_2_O --> 3HCO_3_^-^ + 4NH_4_^+^ [24] | NO2- | CH4 | 17890 | -800 | 12.01 | 11.75 | 11.54 | 11.49 | 11.28 |
| 70 | 3CO(g) + NO_2_^-^ + 2H^+^ + H_2_O --> 3CO_2_(g) + NH_4_^+^ [6] | NO2- | CO | 17600 | -560 | 13.48 | 13.30 | 13.16 | 13.12 | 12.98 |
| *71* | *H_2_O + NO_2_^-^ + 2H^+^ --> NH_4_^+^ + (3/2)O_2_(aq) [6]* | *NO2-* | *N/A* | *-7410* | *-430* | *-10.57* | *-10.71* | *-10.82* | *-10.85* | *-10.96* |
| *72* | *4NO_2_^-^ + H_2_O + 2H^+^ --> NH_4_^+^ + 3NO_3_^-^ [6]* | *NO2-* | *N/A* | *-20* | *-400* | *-2.96* | *-3.09* | *-3.19* | *-3.22* | *-3.32* |
| 73 | H_2_(g) + 2SULFUR + Fe^+2^ --> PYRITE + 2H^+^ [2] | S | H2 | 2450 | 1200 | 11.27 | 11.66 | 11.97 | 12.04 | 12.36 |
| *74* | *NH_4_^+^ + 6SULFUR + 3Fe^+2^ + 2H_2_O --> 3PYRITE + NO_2_^-^ + 8H^+^ [6]* | *S* | *NH4+* | *-14030* | *1640* | *-1.98* | *-1.44* | *-1.02* | *-0.92* | *-0.49* |
| *75* | *NH_4_^+^ + 8SULFUR + 4Fe^+2^ + 3H_2_O --> 4PYRITE + NO_3_^-^ + 10H^+^ [8]* | *S* | *NH4+* | *-13920* | *1520* | *-2.75* | *-2.25* | *-1.86* | *-1.77* | *-1.37* |
| *76* | *NO_2_^-^ + 2SULFUR + Fe^+2^ + H_2_O --> PYRITE + NO_3_^-^ + 2H^+^ [2]* | *S* | *NO2-* | *-13920* | *1210* | *-5.03* | *-4.63* | *-4.32* | *-4.25* | *-3.93* |
| 77 | 3Fe^+2^ + 2SULFUR + 3H_2_O --> PYRITE + HEMATITE + 6H^+^ [2] | S | Fe+2 | -16720 | 3650 | 10.11 | 11.30 | 12.24 | 12.46 | 13.42 |
| 78 | 4Fe^+2^ + 2SULFUR + 4H_2_O --> PYRITE + MAGNETITE + 8H^+^ [2] | S | Fe+2 | -25950 | 4870 | 9.84 | 11.44 | 12.68 | 12.99 | 14.26 |
| 79 | 3Fe^+2^ + 2SULFUR + 4H_2_O --> PYRITE + 2GOETHITE + 6H^+^ [2] | S | Fe+2 | -16630 | 3650 | 10.20 | 11.39 | 12.33 | 12.55 | 13.51 |
| 80 | 2MAGNETITE + 2SULFUR + Fe^+2^ + H_2_O --> PYRITE + 3HEMATITE + 2H^+^ [2] | S | Magnetite | 1760 | 1220 | 10.73 | 11.13 | 11.44 | 11.51 | 11.83 |
| 81 | 2MAGNETITE + 2SULFUR + Fe^+2^ + 4H_2_O --> PYRITE + 6GOETHITE + 2H^+^ [2] | S | Magnetite | 2010 | 1230 | 11.05 | 11.45 | 11.77 | 11.84 | 12.17 |
| 82 | CH_4_(g) + 6SULFUR + 3Fe^+2^ + H_2_O --> 3PYRITE + CO(g) + 6H^+^ [6] | S | CH4 | 800 | 1230 | 9.84 | 10.24 | 10.56 | 10.63 | 10.96 |
| 83 | CH_4_(g) + 8SULFUR + 4Fe^+2^ + 2H_2_O --> 4PYRITE + CO_2_(g) + 8H^+^ [8] | S | CH4 | 1670 | 1150 | 10.12 | 10.50 | 10.79 | 10.86 | 11.17 |
| 84 | CH_4_(g) + 8SULFUR + 4Fe^+2^ + 3H_2_O --> 4PYRITE + HCO_3_^-^ +9H^+^ [8] | S | CH4 | -450 | 1530 | 10.80 | 11.30 | 11.69 | 11.78 | 12.18 |
| 85 | CO(g) + 2SULFUR + Fe^+2^ + H_2_O --> PYRITE + CO_2_(g) + 2H^+^ [2] | S | CO | 3880 | 1100 | 11.97 | 12.33 | 12.61 | 12.67 | 12.96 |
| *86* | *H_2_O + 2SULFUR + Fe^+2^ --> PYRITE + 2H^+^ + (1/2)O_2_(aq) [2]* | *S* | *N/A* | *-21570* | *1205* | *-12.71* | *-12.32* | *-12.01* | *-11.94* | *-11.62* |
| 87 | 7SULFUR + 3Fe^+2^ + 4H_2_O --> 3PYRITE + SO_4_^-2^ + 8H^+^ [6] | S | N/A | -2120 | 1790 | 11.04 | 11.62 | 12.08 | 12.19 | 12.66 |
| 88 | SULFUR + H_2_S(aq) + Fe^+2^ --> PYRITE + 2H^+^ [1] | S | H2S | -3680 | 2130 | 11.98 | 12.67 | 13.22 | 13.35 | 13.91 |
| 89 | H_2_(g) + SULFUR --> H_2_S(aq) [2] | S | H2 | 5010 | 0 | 5.01 | 5.01 | 5.01 | 5.01 | 5.01 |
| *90* | *NH_4_^+^ + 3SULFUR + 2H_2_O --> 3H_2_S(aq) + NO_2_^-^ + 2H^+^ [6]* | *S* | *NH4+* | *-12470* | *620* | *-7.91* | *-7.71* | *-7.55* | *-7.51* | *-7.35* |
| *91* | *NH_4_^+^ + 4SULFUR + 2H_2_O --> 4H_2_S(aq) + NO_3_^-^ + 2H^+^ [8]* | *S* | *NH4+* | *-12560* | *560* | *-8.44* | *-8.26* | *-8.12* | *-8.08* | *-7.94* |
| *92* | *NO_2_^-^ + SULFUR + H_2_O --> H_2_S(aq) + NO_3_^-^ [2]* | *S* | *NO2-* | *-12810* | *300* | *-10.61* | *-10.51* | *-10.43* | *-10.41* | *-10.33* |
| 93 | 2Fe^+2^ + SULFUR + 3H_2_O --> HEMATITE + H_2_S(aq) + 4H^+^ [2] | S | Fe+2 | -14860 | 2590 | 4.18 | 5.03 | 5.69 | 5.85 | 6.53 |
| 94 | 3Fe^+2^ + SULFUR + 4H_2_O --> MAGNETITE + H_2_S(aq) + 6H^+^ [2] | S | Fe+2 | -24100 | 3800 | 3.83 | 5.08 | 6.05 | 6.28 | 7.28 |
| 95 | 2Fe^+2^ + SULFUR + 4H_2_O --> 2GOETHITE + H_2_S(aq) + 4H^+^ [2] | S | Fe+2 | -14780 | 2590 | 4.26 | 5.11 | 5.77 | 5.93 | 6.61 |
| 96 | 2MAGNETITE + SULFUR + H_2_O --> 3HEMATITE + H_2_S(aq) [2] | S | Magnetite | 3190 | 250 | 5.03 | 5.11 | 5.17 | 5.19 | 5.25 |
| 97 | 2MAGNETITE + SULFUR + 4H_2_O --> 6GOETHITE + H_2_S(aq) [2] | S | Magnetite | 3520 | 240 | 5.28 | 5.36 | 5.42 | 5.44 | 5.50 |
| 98 | CH_4_(g) + 3SULFUR + H_2_O --> CO(g) + 3H_2_S(aq) [6] | S | CH4 | 3320 | 30 | 3.54 | 3.55 | 3.56 | 3.56 | 3.57 |
| 99 | CH_4_(g) + 4SULFUR + 2H_2_O --> CO_2_(g) + 4H_2_S(aq) [8] | S | CH4 | 4140 | -40 | 3.85 | 3.83 | 3.82 | 3.82 | 3.81 |
| 100 | CH_4_(g) + 4SULFUR + 3H_2_O --> HCO_3_^-^ + 4H_2_S(aq) + H^+^ [8] | S | CH4 | 2700 | 230 | 4.39 | 4.47 | 4.52 | 4.54 | 4.60 |
| 101 | CO(g) + SULFUR + H_2_O --> CO_2_(g) + H_2_S(aq) [2] | S | CO | 6500 | -150 | 5.40 | 5.35 | 5.31 | 5.30 | 5.26 |
| *102* | *H_2_O + SULFUR --> H_2_S(aq) + (1/2)O2(aq) [2]* | *S* | *N/A* | *-20150* | *260* | *-18.24* | *-18.15* | *-18.09* | *-18.07* | *-18.00* |
| 103 | 4SULFUR + 4H_2_O --> SO_4_-2 + 3H_2_S(aq) + 2H^+^ [6] | S | N/A | -810 | 850 | 5.44 | 5.72 | 5.93 | 5.99 | 6.21 |
| *104* | *H_2_(g) + PYRITE + 2H^+^ --> Fe^+2^ + 2H_2_S(aq) [2]* | *Pyrite* | *H2* | *7380* | *-1130* | *-0.93* | *-1.30* | *-1.58* | *-1.65* | *-1.95* |
| *105* | *NH_4_^+^ + 3PYRITE + 4H^+^ + 2H_2_O --> 6H_2_S(aq) + NO_2_^-^ + 3Fe^+2^ [6]* | *Pyrite* | *NH4+* | *-10400* | *-510* | *-14.15* | *-14.32* | *-14.45* | *-14.48* | *-14.61* |
| *106* | *NH_4_^+^ + 4PYRITE + 6H^+^ + 3H_2_O --> 8H_2_S(aq) + NO_3_^-^ + 4Fe^+2^ [8]* | *Pyrite* | *NH4+* | *-10640* | *-570* | *-14.83* | *-15.02* | *-15.16* | *-15.20* | *-15.35* |
| *107* | *NO_2_^-^ + PYRITE + 2H^+^ + H_2_O --> 2H_2_S(aq) + Fe^+2^ + NO_3_^-^ [2]* | *Pyrite* | *NO2-* | *-11130* | *-780* | *-16.86* | *-17.12* | *-17.32* | *-17.37* | *-17.57* |
| *108* | *Fe^+2^ + PYRITE + 3H_2_O --> HEMATITE + 2H_2_S(aq) + 2H^+^ [2]* | *Pyrite* | *Fe+2* | *-13020* | *1520* | *-1.85* | *-1.35* | *-0.96* | *-0.87* | *-0.47* |
| *109* | *2Fe^+2^ + PYRITE + 4H_2_O --> MAGNETITE + 2H_2_S(aq) + 4H^+^ [2]* | *Pyrite* | *Fe+2* | *-22260* | *2740* | *-2.12* | *-1.22* | *-0.52* | *-0.35* | *0.36* |
| *110* | *Fe^+2^ + PYRITE + 4H_2_O --> 2GOETHITE + 2H_2_S(aq) + 2H^+^ [2]* | *Pyrite* | *Fe+2* | *-12940* | *1520* | *-1.77* | *-1.27* | *-0.88* | *-0.79* | *-0.39* |
| *111* | *MAGNETITE + PYRITE + 2H_2_O --> 2HEMATITE + 2H_2_S(aq) [2]* | *Pyrite* | *Magnetite* | *-4695* | *520* | *-0.87* | *-0.70* | *-0.57* | *-0.54* | *-0.40* |
| *112* | *MAGNETITE + PYRITE + 4H_2_O --> 4GOETHITE + 2H_2_S(aq) [2]* | *Pyrite* | *Magnetite* | *-4470* | *510* | *-0.72* | *-0.55* | *-0.42* | *-0.39* | *-0.26* |
| *113* | *CH_4_(g) + 3PYRITE + 6H^+^ + H_2_O --> CO(g) + 3Fe^+2^ + 6H_2_S(aq) [8]* | *Pyrite* | *CH4* | *5700* | *-1120* | *-2.53* | *-2.90* | *-3.18* | *-3.25* | *-3.55* |
| *114* | *CH_4_(g) + 4PYRITE + 8H^+^ + 2H_2_O --> CO_2_(g) + 4Fe^+2^ + 8H_2_S(aq) [8]* | *Pyrite* | *CH4* | *6500* | *-1180* | *-2.17* | *-2.56* | *-2.86* | *-2.93* | *-3.24* |
| *115* | *CH_4_(g) + 4PYRITE + 7H^+^ + 3H_2_O --> 4Fe^+2^ + HCO_3_^-^ + 8H_2_S(aq) [8]* | *Pyrite* | *CH4* | *4820* | *-870* | *-1.57* | *-1.86* | *-2.08* | *-2.14* | *-2.36* |
| *116* | *CO(g) + PYRITE + 2H^+^ + H_2_O --> Fe^+2^ + CO_2_(g) + 2H_2_S(aq) [2]* | *Pyrite* | *CO* | *8860* | *-1285* | *-0.58* | *-1.01* | *-1.33* | *-1.41* | *-1.75* |
| *117* | *H_2_O + PYRITE + 2H^+^ --> Fe^+2^ + 2H_2_S(aq) + (1/2)O_2_(aq) [2]* | *Pyrite* | *N/A* | *-17540* | *-980* | *-24.74* | *-25.06* | *-25.31* | *-25.38* | *-25.63* |
| *118* | *4PYRITE + 6H^+^ + 4H_2_O --> SO_4_^-2^ + 7H_2_S(aq) + 4Fe^+2^ [7]* | *Pyrite* | *N/A* | *1865* | *-595* | *-2.51* | *-2.70* | *-2.86* | *-2.89* | *-3.05* |
| *119* | *3H_2_(g) + SO_4_^-2^ + 2H^+^ --> SULFUR + 4H_2_O [6]* | *SO4-2* | *H2* | *4290* | *-540* | *0.32* | *0.14* | *0.01* | *-0.03* | *-0.17* |
| *120* | *SO_4_^-2^ + NH_4_^+^ --> SULFUR + NO_2_^-^ + 2H_2_O [6]* | *SO4-2* | *NH4+* | *-11880* | *-175* | *-13.17* | *-13.22* | *-13.27* | *-13.28* | *-13.32* |
| *121* | *4SO_4_^-2^ + 3NH_4_^+^ + 2H^+^ --> 4SULFUR + 3NO_3_^-^ + 7H_2_O [24]* | *SO4-2* | *NH4+* | *-11790* | *-300* | *-14.00* | *-14.09* | *-14.17* | *-14.19* | *-14.27* |
| *122* | *SO_4_^-2^ + 3NO_2_^-^ + 2H^+^ --> SULFUR + 3NO_3_^-^ + H_2_O [6]* | *SO4-2* | *NO2-* | *-11630* | *-620* | *-16.19* | *-16.39* | *-16.55* | *-16.59* | *-16.75* |
| **123** | **SO_4_^-2^ + 9Fe^+2^ + 8H_2_O --> SULFUR + 3MAGNETITE + 16H^+^ [6]** | **SO4-2** | **Fe+2** | **-23830** | **3080** | **-1.19** | **-0.18** | **0.60** | **0.79** | **1.60** |
| **124** | **6Fe^+2^ + SO_4_^-2^ + 5H_2_O --> SULFUR + 3HEMATITE + 10H^+^ [6]** | **SO4-2** | **Fe+2** | **-14600** | **1870** | **-0.86** | **-0.24** | **0.23** | **0.35** | **0.84** |
| **125** | **6Fe^+2^ + SO_4_^-2^ + 8H_2_O --> SULFUR + 6GOETHITE + 10H^+^ [6]** | **SO4-2** | **Fe+2** | **-14510** | **1860** | **-0.84** | **-0.23** | **0.25** | **0.36** | **0.85** |
| *126* | *6MAGNETITE + SO_4_^-2^ + 2H^+^ --> SULFUR + 9HEMATITE + H_2_O [6]* | *SO4-2* | *Magnetite* | *3990* | *-600* | *-0.42* | *-0.62* | *-0.77* | *-0.81* | *-0.96* |
| *127* | *6MAGNETITE + SO_4_^-2^ + 2H^+^ + 8H_2_O --> SULFUR + 18GOETHITE [6]* | *SO4-2* | *Magnetite* | *4330* | *-610* | *-0.15* | *-0.35* | *-0.51* | *-0.55* | *-0.71* |
| *128* | *CH_4_(g) + SO_4_^-2^ + 2H^+^ --> SULFUR + CO(g) + 3H_2_O [6]* | *SO4-2* | *CH4* | *2590* | *-550* | *-1.45* | *-1.63* | *-1.77* | *-1.81* | *-1.95* |
| *129* | *3CH_4_(g) + 4SO_4_^-2^ + 8H^+^ --> 4SULFUR + 3CO_2_(g) + 10H_2_O [24]* | *SO4-2* | *CH4* | *3390* | *-590* | *-0.95* | *-1.14* | *-1.29* | *-1.33* | *-1.48* |
| *130* | *3CH_4_(g) + 4SO_4_^-2^ + 5H^+^ --> 4SULFUR + 3HCO_3_^-^ + 7H_2_O [24]* | *SO4-2* | *CH4* | *4670* | *-750* | *-0.84* | *-1.09* | *-1.28* | *-1.33* | *-1.52* |
| **131** | **3CO(g) + SO_4_^-2^ + 2H^+^ --> SULFUR + 3CO_2_(g) + H_2_O [6]** | **SO4-2** | **CO** | **5700** | **-700** | **0.56** | **0.33** | **0.15** | **0.10** | **-0.08** |
| *132* | *SO_4_^-2^ + 2H^+^ --> SULFUR + (3/2)O_2_(aq)+ H_2_O [6]* | *SO4-2* | *N/A* | *-19400* | *-590* | *-23.74* | *-23.93* | *-24.08* | *-24.12* | *-24.27* |
| *133* | *SO_4_^-2^ + 3H_2_S(aq) + 2H^+^ --> 4SULFUR + 4H_2_O [6]* | *SO4-2* | *H2S* | *800* | *-850* | *-5.45* | *-5.73* | *-5.94* | *-6.00* | *-6.22* |
| 134 | 7H_2_(g) + 2SO_4_^-2^ + Fe^+2^ + 2H^+^ --> PYRITE + 8H_2_O [14] | SO4-2 | H2 | 3900 | -250 | 2.06 | 1.98 | 1.92 | 1.90 | 1.84 |
| *135* | *3Fe^+2^ + 6SO_4_^-2^ + 7NH_4_^+^ --> 3PYRITE + 7NO_2_^-^ + 8H^+^ + 10H_2_O [42]* | *SO4-2* | *NH4+* | *-12240* | *100* | *-11.51* | *-11.47* | *-11.45* | *-11.44* | *-11.41* |
| *136* | *4Fe^+2^ + 8SO_4_^-2^ + 7NH_4_^+^ --> 4PYRITE + 7NO_3_^-^ + 6H^+^ + 11H_2_O [56]* | *SO4-2* | *NH4+* | *-12100* | *-40* | *-12.39* | *-12.41* | *-12.42* | *-12.42* | *-12.43* |
| *137* | *PYRITE + 7NO_3_- + H_2_O --> Fe+2 + 2 SO_4_-2 + 7 NO_2_- + H_2_O [14]* | *SO4-2* | *NO2-* | *-12100* | *-340* | *-14.60* | *-14.71* | *-14.80* | *-14.82* | *-14.91* |
| **138** | **2SO4-2 + 22Fe+2 + 20H2O --> PYRITE + 7MAGNETITE + 40H+ [14]** | **SO4-2** | **Fe+2** | **-24130** | **3330** | **0.35** | **1.44** | **2.29** | **2.49** | **3.37** |
| **139** | **2SO4-2 + 15Fe+2 + 13H2O --> PYRITE + 7HEMATITE + 26H+ [14]** | **SO4-2** | **Fe+2** | **-14890** | **2120** | **0.69** | **1.39** | **1.93** | **2.06** | **2.61** |
| **140** | **2SO4-2 + 15Fe+2 + 20H2O --> PYRITE + 14GOETHITE + 26H+ [14]** | **SO4-2** | **Fe+2** | **-14810** | **2120** | **0.77** | **1.47** | **2.01** | **2.14** | **2.69** |
| 141 | 14MAGNETITE + Fe^+2^ + 2SO_4_^-2^ + 2H^+^ --> PYRITE + 21HEMATITE +H_2_O [14] | SO4-2 | Magnetite | 3580 | -310 | 1.30 | 1.20 | 1.12 | 1.10 | 1.02 |
| 142 | 14MAGNETITE + Fe^+2^ + 2SO_4_^-2^ + 2H^+^ + 20H_2_O -->PYRITE + 42GOETHITE [14] | SO4-2 | Magnetite | 3830 | -310 | 1.55 | 1.45 | 1.37 | 1.35 | 1.27 |
| **143** | **7CH_4_(g) + 6SO_4_^-2^ + 6H^+^ + 3Fe^+2^ --> 3PYRITE + 7CO(g) + 17H_2_O [42]** | **SO4-2** | **CH4** | **2320** | **-290** | **0.19** | **0.09** | **0.02** | **0.00** | **-0.07** |
| 144 | 7CH_4_(g) + 8SO_4_^-2^ + 8H^+^ + 4Fe^+2^ --> 4PYRITE + 7CO_2_(g) + 18H_2_O [56] | SO4-2 | CH4 | 3120 | -330 | 0.69 | 0.59 | 0.50 | 0.48 | 0.40 |
| 145 | 7CH_4_(g) + 8SO_4_^-2^ + H^+^ + 4Fe^+2^ --> 4PYRITE + 7HCO^-^ + 11H_2_O [56] | SO4-2 | CH4 | 3840 | -410 | 0.83 | 0.69 | 0.59 | 0.56 | 0.45 |
| 146 | 7CO(g) + 2SO_4_^-2^ + 2H^+^ + Fe^+2^ --> PYRITE + 7CO_2_(g) + H_2_O [14] | SO4-2 | CO | 5390 | -420 | 2.30 | 2.17 | 2.06 | 2.03 | 1.92 |
| *147* | *2 SO_4_^-2^ + Fe^+2^ + 2H^+^ --> PYRITE + (7/2)O_2_(aq) + H_2_O [14]* | *SO4-2* | *N/A* | *-19680* | *-340* | *-22.18* | *-22.29* | *-22.38* | *-22.40* | *-22.49* |
| 148 | SO_4_^-2^ + 7H_2_S(aq) + 4Fe^+2^ --> 4PYRITE + 6H^+^ + 4H_2_O [7] | SO4-2 | H2S | -1870 | 590 | 2.47 | 2.66 | 2.81 | 2.85 | 3.00 |
| 149 | 4H_2_(g) + SO_4_^-2^ + 2H^+^ --> H_2_S(aq) + 4H_2_O [8] | SO4-2 | H2 | 4460 | -400 | 1.52 | 1.39 | 1.29 | 1.26 | 1.16 |
| *150* | *3SO_4_^-2^ + 4NH_4_^+^ --> 3H_2_S(aq) + 4NO_2_^-^ + 2H^+^ + 4H_2_O [24]* | *SO4-2* | *NH4+* | *-12040* | *30* | *-11.82* | *-11.81* | *-11.80* | *-11.80* | *-11.79* |
| *151* | *SO_4_^-2^ + NH_4_^+^ --> H_2_S(aq) + NO_3_^-^ + H_2_O [8]* | *SO4-2* | *NH4+* | *-11970* | *-80* | *-12.56* | *-12.58* | *-12.60* | *-12.61* | *-12.63* |
| *152* | *SO_4_^-2^ + 4NO_2_^-^ + 2H^+^ --> H_2_S(aq) + 4NO_3_^-^ [8]* | *SO4-2* | *NO2-* | *-11900* | *-400* | *-14.84* | *-14.97* | *-15.07* | *-15.10* | *-15.20* |
| **153** | **SO_4_^-2^ + 12Fe^+2^ + 12H_2_O --> 4MAGNETITE + H_2_S(aq) + 22H^+^ [8]** | **SO4-2** | **Fe+2** | **-23890** | **3260** | **0.07** | **1.14** | **1.97** | **2.17** | **3.03** |
| **154** | **SO_4_^-2^ + 8Fe^+2^ + 8H_2_O --> 4HEMATITE + H_2_S(aq) + 14H^+^ [8]** | **SO4-2** | **Fe+2** | **-14650** | **2040** | **0.34** | **1.01** | **1.53** | **1.66** | **2.19** |
| **155** | **SO_4_^-2^ + 8Fe^+2^ + 12H_2_O --> 8GOETHITE + H_2_S(aq) + 14H^+^ [8]** | **SO4-2** | **Fe+2** | **-14570** | **2040** | **0.42** | **1.09** | **1.61** | **1.74** | **2.27** |
| 156 | 8MAGNETITE + SO_4_^-2^ + 2H^+^ --> 12HEMATITE + H_2_S(aq) [8] | SO4-2 | Magnetite | 3790 | -380 | 1.00 | 0.87 | 0.78 | 0.75 | 0.65 |
| 157 | 8MAGNETITE + SO_4_^-2^ + 2H^+^ + 12H_2_O --> 24GOETHITE + H_2_S(aq) [8] | SO4-2 | Magnetite | 4130 | -400 | 1.19 | 1.06 | 0.96 | 0.93 | 0.83 |
| *158* | *4CH_4_(g) + 3SO_4_^-2^ + 6H^+^ --> 4CO(g) + 3H_2_S(aq) + 8H_2_O [24]* | *SO4-2* | *CH4* | *2780* | *-410* | *-0.23* | *-0.37* | *-0.47* | *-0.50* | *-0.61* |
| **159** | **CH_4_(g) + SO_4_^-2^ + 2H^+^ --> CO_2_(g) + H_2_S(aq) + 2H_2_O [8]** | **SO4-2** | **CH4** | **3580** | **-450** | **0.27** | **0.12** | **0.01** | **-0.02** | **-0.14** |
| **160** | **CH_4_(g) + SO_4_^-2^ + H^+^ --> HCO_3_^-^ + H_2_S(aq) + H_2_O [8]** | **SO4-2** | **CH4** | **4180** | **-510** | **0.43** | **0.26** | **0.13** | **0.10** | **-0.03** |
| **161** | **4CO(g) + SO_4_^-2^ + 2H^+^ --> 4CO_2_(g) + H_2_S(aq) [8]** | **SO4-2** | **CO** | **5960** | **-590** | **1.62** | **1.43** | **1.28** | **1.24** | **1.09** |
| *162* | *SO_4_^-2^ + 2H^+^ --> H_2_S(aq) + 2O_2_(aq) [8]* | *SO4-2* | *N/A* | *-19590* | *-375* | *-22.35* | *-22.47* | *-22.56* | *-22.59* | *-22.69* |
| **163** | **MAGNETITE + H_2_(g) + 6H^+^ --> 3Fe^+2^ + 4H_2_O [2]** | **Magnetite** | **H2** | **26620** | **-3330** | **2.14** | **1.05** | **0.20** | **0.00** | **-0.88** |
| *164* | *3MAGNETITE + NH_4_^+^ + 16H^+^ --> 9Fe^+2^ + NO_2_^-^ + 10H_2_O [6]* | *Magnetite* | *NH4+* | *11830* | *-3250* | *-12.06* | *-13.12* | *-13.95* | *-14.15* | *-15.01* |
| *165* | *4MAGNETITE + NH_4_^+^ + 22H^+^ --> 12Fe^+2^ + NO_3_^-^ + 13H_2_O [8]* | *Magnetite* | *NH4+* | *11900* | *-3410* | *-13.16* | *-14.28* | *-15.15* | *-15.36* | *-16.26* |
| *166* | *MAGNETITE + NO_2_^-^ + 6H^+^ --> 3Fe^+2^ + NO_3_^-^ + 3H_2_O [2]* | *Magnetite* | *NO2-* | *11950* | *-3700* | *-15.25* | *-16.46* | *-17.40* | *-17.63* | *-18.60* |
| **167** | **MAGNETITE + 2H_2_S(aq) + 4H^+^ --> PYRITE + 2Fe^+2^ + 4H_2_O [2]** | **Magnetite** | **H2S** | **22260** | **-2740** | **2.12** | **1.22** | **0.52** | **0.35** | **-0.36** |
| *168* | *MAGNETITE + H_2_S(aq) + 6H^+^ --> 3Fe^+2^ + SULFUR + 4H_2_O [2]* | *Magnetite* | *H2S* | *24100* | *-3800* | *-3.83* | *-5.08* | *-6.05* | *-6.28* | *-7.28* |
| *169* | *4MAGNETITE + H_2_S(aq) + 22H^+^ --> SO_4_^-2^ + 12Fe^+2^ + 12H_2_O [8]* | *Magnetite* | *H2S* | *23890* | *-3260* | *-0.07* | *-1.14* | *-1.97* | *-2.17* | *-3.03* |
| *170* | *PYRITE + MAGNETITE + 8H^+^ --> 4Fe^+2^ + 2SULFUR + 4H_2_O [2]* | *Magnetite* | *Pyrite* | *25950* | *-4870* | *-9.84* | *-11.44* | *-12.68* | *-12.99* | *-14.26* |
| *171* | *PYRITE + 7MAGNETITE + 40H^+^ --> 2SO_4_^-2^ + 22Fe^+2^ + 20H_2_O [14]* | *Magnetite* | *Pyrite* | *24130* | *-3330* | *-0.35* | *-1.44* | *-2.29* | *-2.49* | *-3.37* |
| *172* | *SULFUR + 3MAGNETITE + 16H^+^ --> SO_4_^-2^ + 9Fe^+2^ + 8H_2_O [6]* | *Magnetite* | *S* | *23830* | *-3075* | *1.23* | *0.22* | *-0.56* | *-0.75* | *-1.56* |
| *173* | *3MAGNETITE + CH_4_(g) + 18H^+^ --> CO(g) + 9Fe^+2^ + 11H_2_O [6]* | *Magnetite* | *CH4* | *24840* | *-3470* | *-0.66* | *-1.80* | *-2.69* | *-2.90* | *-3.81* |
| *174* | *4MAGNETITE + CH_4_(g) + 24H^+^ --> CO_2_(g) + 12Fe^+2^ + 14H_2_O [8]* | *Magnetite* | *CH4* | *25400* | *-3370* | *0.63* | *-0.47* | *-1.33* | *-1.54* | *-2.43* |
| *175* | *4MAGNETITE + CH_4_(g) + 23H^+^ --> HCO_3_^-^ + 12Fe^+2^ + 13H_2_O [8]* | *Magnetite* | *CH4* | *33500* | *-4620* | *-0.46* | *-1.97* | *-3.15* | *-3.44* | *-4.65* |
| *176* | *MAGNETITE + CO(g) + 6H^+^ --> CO_2_(g) + 3Fe^+2^ + 3H_2_O [2]* | *Magnetite* | *CO* | *27930* | *-3600* | *1.47* | *0.29* | *-0.63* | *-0.85* | *-1.80* |
| *177* | *MAGNETITE + 6H^+^ --> 3Fe^+2^ + (1/2)O_2_(aq) + 3H_2_O [2]* | *Magnetite* | *O2 as product* | *4770* | *-3720* | *-22.57* | *-23.79* | *-24.74* | *-24.97* | *-25.95* |
| **178** | **HEMATITE + H_2_(g) + 4H^+^ --> 2Fe^+2^ + 3H_2_O [2]** | **Hematite** | **H2** | **17870** | **-2200** | **1.70** | **0.98** | **0.42** | **0.28** | **-0.30** |
| *179* | *3HEMATITE + NH_4_^+^ + 10H+ --> 6Fe^+2^ + NO_2_^-^ + 7H_2_O [6]* | *Hematite* | *NH4+* | *2615* | *-2025* | *-12.27* | *-12.93* | *-13.45* | *-13.57* | *-14.11* |
| *180* | *4HEMATITE + NH_4_^+^ + 14H^+^ --> 8Fe^+2^ + NO_3_^-^ + 9H_2_O [8]* | *Hematite* | *NH4+* | *2930* | *-2210* | *-13.31* | *-14.04* | *-14.60* | *-14.74* | *-15.32* |
| *181* | *HEMATITE + NO_2_^-^ + 4H^+^ --> 2Fe^+2^ + NO_3_^-^ + 2H_2_O [2]* | *Hematite* | *NO2-* | *3020* | *-2515* | *-15.47* | *-16.29* | *-16.93* | *-17.09* | *-17.75* |
| **182** | **HEMATITE + 2H_2_S(aq) + 2H^+^ --> PYRITE + Fe^+2^ + 3H_2_O [2]** | **Hematite** | **H2S** | **13020** | **-1520** | **1.85** | **1.35** | **0.96** | **0.87** | **0.47** |
| *183* | *HEMATITE + H_2_S(aq) + 4H^+^ --> 2Fe^+2^ + SULFUR + 3H_2_O [2]* | *Hematite* | *H2S* | *14860* | *-2590* | *-4.18* | *-5.03* | *-5.69* | *-5.85* | *-6.53* |
| *184* | *4HEMATITE + H_2_S(aq) + 14H^+^ --> SO_4_^-2^ + 8Fe^+2^ + 8H_2_O [8]* | *Hematite* | *H2S* | *14660* | *-2050* | *-0.41* | *-1.08* | *-1.60* | *-1.73* | *-2.27* |
| *185* | *PYRITE + HEMATITE + 6H^+^ --> 3Fe^+2^ + 2SULFUR + 3H_2_O [2]* | *Hematite* | *Pyrite* | *16710* | *-3650* | *-10.12* | *-11.31* | *-12.25* | *-12.47* | *-13.43* |
| *186* | *PYRITE + 7HEMATITE + 26H^+^ --> 2SO_4_^-2^ + 15Fe^+2^ + 13H_2_O [14]* | *Hematite* | *Pyrite* | *14890* | *-2115* | *-0.66* | *-1.35* | *-1.89* | *-2.02* | *-2.57* |
| **187** | **SULFUR + 3HEMATITE + 10H^+^ --> 6Fe^+2^ + SO_4_^-2^ + 5H_2_O [6]** | **Hematite** | **S** | **14590** | **-1860** | **0.92** | **0.31** | **-0.17** | **-0.28** | **-0.77** |
| *188* | *3HEMATITE + CH_4_(g) + 12H^+^ --> CO(g) + 6Fe^+2^ + 8H_2_O [6]* | *Hematite* | *CH4* | *16130* | *-2295* | *-0.74* | *-1.49* | *-2.08* | *-2.22* | *-2.82* |
| **189** | **4HEMATITE + CH_4_(g) + 16H^+^ --> CO_2_(g) + 8Fe^+2^ + 10H_2_O [8]** | **Hematite** | **CH4** | **16770** | **-2250** | **0.23** | **-0.51** | **-1.08** | **-1.22** | **-1.81** |
| *190* | *4HEMATITE + CH_4_(g) + 15H^+^ --> HCO_3_^-^ + 8Fe^+2^ + 9H_2_O [8]* | *Hematite* | *CH4* | *21980* | *-3040* | *-0.36* | *-1.36* | *-2.14* | *-2.32* | *-3.12* |
| **191** | **HEMATITE + CO(g) + 4H^+^ --> CO_2_(g) + 2Fe^+2^ + 2H_2_O [2]** | **Hematite** | **CO** | **19215** | **-2430** | **1.35** | **0.56** | **-0.06** | **-0.21** | **-0.85** |
| *192* | *HEMATITE + 4H^+^ --> 2Fe^+2^ + (1/2)O_2_(aq) + 2H_2_O [2]* | *Hematite* | *N/A* | *-4570* | *-2490* | *-22.87* | *-23.69* | *-24.32* | *-24.48* | *-25.13* |
| **193** | **3HEMATITE + H_2_(g) --> 2MAGNETITE + H_2_O [2]** | **Hematite** | **H2** | **830** | **-50** | **0.46** | **0.45** | **0.43** | **0.43** | **0.42** |
| *194* | *9HEMATITE + NH_4_^+^ --> 6MAGNETITE + NO_2_^-^ + 2H^+^ + H_2_O* | *Hematite* | *NH4+* | *-15820* | *420* | *-12.73* | *-12.60* | *-12.49* | *-12.46* | *-12.35* |
| *195* | *12HEMATITE + NH_4_^+^ --> 8MAGNETITE + NO_3_^-^ + 2H^+^ + H_2_* | *Hematite* | *NH4+* | *-15720* | *300* | *-13.52* | *-13.42* | *-13.34* | *-13.32* | *-13.24* |
| *196* | *3HEMATITE + NO_2_- --> 2MAGNETITE + NO_3_^-^ [2]* | *Hematite* | *NO2-* | *-15640* | *-20* | *-15.79* | *-15.79* | *-15.80* | *-15.80* | *-15.81* |
| 197 | 2HEMATITE + 2H_2_S(aq) --> PYRITE + MAGNETITE + 2H_2_O [2] | Hematite | H2S | 4700 | -520 | 0.88 | 0.71 | 0.57 | 0.54 | 0.41 |
| *198* | *3HEMATITE + H_2_S(aq) --> 2MAGNETITE + SULFUR + H_2_O [2]* | *Hematite* | *H2S* | *-3190* | *-250* | *-5.03* | *-5.11* | *-5.17* | *-5.19* | *-5.25* |
| *199* | *12HEMATITE + H_2_S(aq) --> 8MAGNETITE + SO_4_^-2^ + 2H^+^ [8]* | *Hematite* | *H2S* | *-3790* | *380* | *-1.00* | *-0.87* | *-0.78* | *-0.75* | *-0.65* |
| *200* | *PYRITE + 3HEMATITE + 2H^+^ --> 2MAGNETITE + 2SULFUR [2]* | *Hematite* | *Pyrite* | *-1760* | *-1220* | *-10.73* | *-11.13* | *-11.44* | *-11.51* | *-11.83* |
| *201* | *PYRITE + 21HEMATITE + H_2_O --> 14MAGNETITE + Fe^+2^ + 2SO_4_^-2^ + 2H^+^ [14]* | *Hematite* | *Pyrite* | *-3580* | *310* | *-1.30* | *-1.20* | *-1.12* | *-1.10* | *-1.02* |
| 202 | SULFUR + 9HEMATITE + H_2_O --> 6MAGNETITE + SO_4_^-2^ + 2H^+^ [14] | Hematite | S | -3990 | 600 | 0.42 | 0.62 | 0.77 | 0.81 | 0.96 |
| *203* | *9HEMATITE + CH_4_(g) --> 6MAGNETITE + CO(g) + 2H_2_O [6]* | *Hematite* | *CH4* | *-1240* | *20* | *-1.09* | *-1.09* | *-1.08* | *-1.08* | *-1.07* |
| *204* | *12HEMATITE + CH_4_(g) --> 8MAGNETITE + CO_2_(g) + 2H_2_O [8]* | *Hematite* | *CH4* | *-510* | *-10* | *-0.58* | *-0.59* | *-0.59* | *-0.59* | *-0.59* |
| *205* | *12HEMATITE + CH_4_(g) --> 8MAGNETITE + HCO_3_^-^ + H^+^ + H_2_O [8]* | *Hematite* | *CH4* | *-940* | *100* | *-0.21* | *-0.17* | *-0.15* | *-0.14* | *-0.11* |
| 206 | 3HEMATITE + CO(g) --> 2MAGNETITE + CO_2_(g) [2] | Hematite | CO | 1880 | -150 | 0.78 | 0.73 | 0.69 | 0.68 | 0.64 |
| *207* | *3HEMATITE --> 2MAGNETITE + (1/2)O_2_(aq) [2]* | *Hematite* | *N/A* | *-23400* | *10* | *-23.33* | *-23.32* | *-23.32* | *-23.32* | *-23.32* |
| **208** | **GOETHITE + (1/2)H_2_(g) + 2H^+^ --> Fe^+2^ + 2H_2_O [1]** | **Goethite** | **H2** | **17860** | **-2220** | **1.54** | **0.81** | **0.25** | **0.11** | **-0.47** |
| *209* | *6GOETHITE + NH_4_^+^ + 10H^+^ --> 6Fe^+2^ + NO_2_^-^ + 10H_2_O [6]* | *Goethite* | *NH4+* | *2530* | *-2025* | *-12.35* | *-13.02* | *-13.53* | *-13.66* | *-14.19* |
| *210* | *8GOETHITE + NH_4_^+^ + 14H^+^ --> 8Fe^+2^ + NO_3_^-^ + 13H_2_O [8]* | *Goethite* | *NH4+* | *2640* | *-2180* | *-13.38* | *-14.10* | *-14.65* | *-14.79* | *-15.36* |
| *211* | *2GOETHITE + NO_2_^-^ + 4H^+^ --> 2Fe^+2^ + NO_3_^-^ + 3H_2_O [2]* | *Goethite* | *NO2-* | *2660* | *-2480* | *-15.57* | *-16.38* | *-17.01* | *-17.17* | *-17.82* |
| **212** | **2GOETHITE + 2H_2_S(aq) + 2H^+^ --> PYRITE + Fe^+2^ + 4H_2_O [2]** | **Goethite** | **H2S** | **12940** | **-1525** | **1.73** | **1.23** | **0.84** | **0.75** | **0.35** |
| *213* | *2GOETHITE + H_2_S(aq) + 4H^+^ --> 2Fe^+2^ + SULFUR + 4H_2_O [2]* | *Goethite* | *H2S* | *14780* | *-2590* | *-4.26* | *-5.11* | *-5.77* | *-5.93* | *-6.61* |
| *214* | *8GOETHITE + H_2_S(aq) + 14H^+^ --> SO_4_^-2^ + 8Fe^+2^ + 12H_2_O [8]* | *Goethite* | *H2S* | *14570* | *-2040* | *-0.42* | *-1.09* | *-1.61* | *-1.74* | *-2.27* |
| *215* | *PYRITE + 2GOETHITE + 6H^+^ --> 3Fe^+2^ + 2SULFUR + 4H_2_O [2]* | *Goethite* | *Pyrite* | *16630* | *-3650* | *-10.20* | *-11.39* | *-12.33* | *-12.55* | *-13.51* |
| *216* | *PYRITE + 14GOETHITE + 26H^+^ --> 2SO_4_^-2^ + 15Fe^+2^ +20H_2_O [14]* | *Goethite* | *Pyrite* | *14810* | *-2120* | *-0.77* | *-1.47* | *-2.01* | *-2.14* | *-2.69* |
| **217** | **SULFUR + 6GOETHITE + 10H^+^ --> 6Fe^+2^ + SO_4_^-2^ + 8H_2_O [6]** | **Goethite** | **S** | **14510** | **-1860** | **0.84** | **0.23** | **-0.25** | **-0.36** | **-0.85** |
| *218* | *6GOETHITE + CH_4_(g) + 12H^+^ --> CO(g) + 6Fe^+2^ + 11H_2_O [6]* | *Goethite* | *CH4* | *16130* | *-2300* | *-0.78* | *-1.53* | *-2.12* | *-2.26* | *-2.86* |
| **219** | **8GOETHITE + CH_4_(g) + 16H^+^ --> CO_2_(g) + 8Fe^+2^ + 14H_2_O [8]** | **Goethite** | **CH4** | **16770** | **-2260** | **0.16** | **-0.58** | **-1.16** | **-1.30** | **-1.89** |
| *220* | *8GOETHITE + CH_4_(g) + 15H^+^ --> HCO_3_- + 8Fe^+2^ + 13H_2_O [8]* | *Goethite* | *CH4* | *21420* | *-2965* | *-0.37* | *-1.35* | *-2.10* | *-2.29* | *-3.06* |
| **221** | **2GOETHITE + CO(g) + 4H^+^ --> CO_2_(g) + 2Fe^+2^ + 3H_2_O [2]** | **Goethite** | **CO** | **19210** | **-2440** | **1.28** | **0.48** | **-0.15** | **-0.30** | **-0.94** |
| *222* | *GOETHITE + 2H^+^ --> Fe^+2^ + (1/4)O_2_(aq)+ (3/2)H_2_O [1]* | *Goethite* | *N/A* | *-4670* | *-2490* | *-22.97* | *-23.79* | *-24.42* | *-24.58* | *-25.23* |
| 223 | 6GOETHITE + H_2_(g) --> 2MAGNETITE + 4H_2_O [2] | Goethite | H2 | 600 | -60 | 0.16 | 0.14 | 0.12 | 0.12 | 0.10 |
| *224* | *18GOETHITE + NH_4_^+^ --> 6MAGNETITE + NO_2_^-^ + 2H^+^ + 10H_2_O [6]* | *Goethite* | *NH4+* | *-16120* | *430* | *-12.96* | *-12.82* | *-12.71* | *-12.68* | *-12.57* |
| *225* | *24GOETHITE + NH_4_^+^ --> 8MAGNETITE + NO_3_^-^ + 2H^+^ + 13H_2_O [8]* | *Goethite* | *NH4+* | *-15970* | *310* | *-13.69* | *-13.59* | *-13.51* | *-13.49* | *-13.41* |
| *226* | *6GOETHITE + NO_2_^-^ --> 2MAGNETITE + NO_3_^-^ + H_2_O [2]* | *Goethite* | *NO2-* | *-15950* | *0* | *-15.95* | *-15.95* | *-15.95* | *-15.95* | *-15.95* |
| **227** | **4GOETHITE + 2H_2_S(aq) --> PYRITE + MAGNETITE + 4H_2_O [2]** | **Goethite** | **H2S** | **4470** | **-510** | **0.72** | **0.55** | **0.42** | **0.39** | **0.26** |
| *228* | *6GOETHITE + H_2_S(aq) --> 2MAGNETITE + SULFUR + 4H_2_O [2]* | *Goethite* | *H2S* | *-3520* | *-240* | *-5.28* | *-5.36* | *-5.42* | *-5.44* | *-5.50* |
| *229* | *24GOETHITE + H_2_S(aq) --> 8MAGNETITE + SO_4_^-2^ + 2H^+^ +12H_2_O [8]* | *Goethite* | *H2S* | *-4130* | *400* | *-1.19* | *-1.06* | *-0.96* | *-0.93* | *-0.83* |
| *230* | *6GOETHITE + 2H^+^ + PYRITE --> 2MAGNETITE + 2SULFUR + Fe^+2^ + 4H_2_O [2]* | *Goethite* | *Pyrite* | *-2190* | *-1160* | *-10.72* | *-11.10* | *-11.39* | *-11.46* | *-11.77* |
| *231* | *42GOETHITE + PYRITE --> 14MAGNETITE + Fe^+2^ + 2SO_4_^-2^ + 2H^+^ + 20H_2_O [14]* | *Goethite* | *Pyrite* | *-3860* | *320* | *-1.51* | *-1.40* | *-1.32* | *-1.30* | *-1.22* |
| **232** | **SULFUR + 18GOETHITE --> 6MAGNETITE + SO_4_^-2^ + 2H^+^ + 8H_2_O [6]** | **Goethite** | **S** | **-4330** | **610** | **0.15** | **0.35** | **0.51** | **0.55** | **0.71** |
| *233* | *18GOETHITE + CH_4_(g) --> 6MAGNETITE + CO(g) + 11H_2_O [6]* | *Goethite* | *CH4* | *-1285* | *-5* | *-1.32* | *-1.32* | *-1.32* | *-1.32* | *-1.33* |
| *234* | *24GOETHITE + CH_4_(g) --> 8MAGNETITE + CO_2_(g) + 14H_2_O [8]* | *Goethite* | *CH4* | *-580* | *-40* | *-0.87* | *-0.89* | *-0.90* | *-0.90* | *-0.91* |
| **235** | **24GOETHITE + CH_4_(g) --> 8MAGNETITE + H^+^ + HCO_3_^-^ + 13H_2_O [8]** | **Goethite** | **CH4** | **-2610** | **330** | **-0.18** | **-0.08** | **0.01** | **0.03** | **0.11** |
| 236 | 6GOETHITE + CO(g) --> 2MAGNETITE + CO_2_(g) + 3H_2_O [2] | Goethite | CO | 1840 | -180 | 0.52 | 0.46 | 0.41 | 0.40 | 0.35 |
| *237* | *6GOETHITE --> 2MAGNETITE + (1/2)O_2_(aq)+ 3H_2_O [2]* | *Goethite* | *N/A* | *-23775* | *35* | *-23.52* | *-23.51* | *-23.50* | *-23.50* | *-23.49* |
| 238 | CO(g) + 3H_2_(g) --> CH_4_(g) + H_2_O | CO | H2 | 1950 | -90 | 1.29 | 1.26 | 1.24 | 1.23 | 1.21 |
| *239* | *CO(g) + NH_4_^+^ + H_2_O --> CH_4_(g) + NO_2_^-^ + 2H^+^ [6]* | *CO* | *NH4+* | *-13950* | *330* | *-11.52* | *-11.42* | *-11.33* | *-11.31* | *-11.23* |
| *240* | *4CO(g) + 3NH_4_^+^ + 5H_2_O --> 4CH_4_(g) + 3NO_3_^-^ + 6H^+^ [24]* | *CO* | *NH4+* | *-14200* | *220* | *-12.58* | *-12.51* | *-12.45* | *-12.44* | *-12.38* |
| *241* | *CO(g) + 3NO_2_^-^ + 2H_2_O --> CH_4_(g) + 3NO_3_^-^ [6]* | *CO* | *NO2-* | *-15160* | *-30* | *-15.38* | *-15.39* | *-15.40* | *-15.40* | *-15.41* |
| 242 | CO(g) + 3Fe^+2^ + 6H_2_S(aq) --> 3PYRITE + CH_4_(g) + 6H^+^ +H_2_O [6] | CO | H2S | -5700 | 1120 | 2.53 | 2.90 | 3.18 | 3.25 | 3.55 |
| *243* | *CO(g) + 3H_2_S(aq) --> CH_4_(g) + 3SULFUR + H_2_O [6]* | *CO* | *H2S* | *-3320* | *-30* | *-3.54* | *-3.55* | *-3.56* | *-3.56* | *-3.57* |
| 244 | 4CO(g) + 3H_2_S(aq) + 8H_2_O --> 4CH_4_(g) + 3SO_4_^-2^ + 6H^+^ [24] | CO | H2S | -2780 | 410 | 0.23 | 0.37 | 0.47 | 0.50 | 0.61 |
| *245* | *3PYRITE + CO(g) + 6H^+^ --> CH_4_(g) + 6SULFUR + 3Fe^+2^ + H_2_O [6]* | *CO* | *Pyrite* | *-800* | *-1230* | *-9.84* | *-10.24* | *-10.56* | *-10.63* | *-10.96* |
| *246* | *3PYRITE + 7CO(g) + 17H_2_O --> 7CH_4_(g) + 6SO_4_^-2^ + 6H^+^ + 3Fe^+2^ [42]* | *CO* | *Pyrite* | *-2320* | *290* | *-0.19* | *-0.09* | *-0.02* | *0.00* | *0.07* |
| 247 | SULFUR + CO(g) + 3H_2_O --> CH_4_(g) + SO_4_^-2^ + 2H^+^ [6] | CO | S | -2590 | 550 | 1.45 | 1.63 | 1.77 | 1.81 | 1.95 |
| **248** | **CO(g) + 9Fe^+2^ + 11H_2_O --> 3MAGNETITE + CH_4_(g) + 18H^+^ [6]** | **CO** | **Fe+2** | **-24840** | **3470** | **0.66** | **1.80** | **2.69** | **2.90** | **3.81** |
| **249** | **CO(g) + 6Fe^+2^ + 8H_2_O --> 3HEMATITE + CH_4_(g) + 12H^+^ [6]** | **CO** | **Fe+2** | **-16130** | **2300** | **0.78** | **1.53** | **2.12** | **2.26** | **2.86** |
| 250 | CO(g) + 6Fe^+2^ + 11H_2_O --> 6GOETHITE + CH_4_(g) + 12H^+^ [6] | CO | Fe+2 | -16130 | 2300 | 0.78 | 1.53 | 2.12 | 2.26 | 2.86 |
| 251 | 6MAGNETITE + CO(g) + 2H_2_O --> 9HEMATITE + CH_4_(g) [6] | CO | Magnetite | 1240 | -30 | 1.02 | 1.01 | 1.00 | 1.00 | 0.99 |
| 252 | 6MAGNETITE + CO(g) + 11H_2_O --> 18GOETHITE + CH_4_(g) [6] | CO | Magnetite | 1285 | 5 | 1.32 | 1.32 | 1.32 | 1.32 | 1.33 |
| *253* | *CO(g) + 2H_2_O --> CH_4_(g) + (3/2)O_2_(aq) [6]* | *CO* | *N/A* | *-22000* | *-25* | *-22.18* | *-22.19* | *-22.20* | *-22.20* | *-22.21* |
| 254 | 4CO(g) + 2H_2_O --> CH_4_(g) + 3CO_2_(g) [6] | CO | *N/A* | 3120 | -170 | 1.87 | 1.81 | 1.77 | 1.76 | 1.72 |
| *255* | *CO_2_(g) + H_2_(g) --> CO(g) + H_2_O* | *CO2* | *H2* | *-1630* | *170* | *-0.38* | *-0.32* | *-0.28* | *-0.27* | *-0.23* |
| *256* | *3CO_2_(g) + NH_4_^+^ --> 3CO(g) + NO_2_^-^ + 2H^+^ + H_2_O [6]* | *CO2* | *NH4+* | *-17600* | *560* | *-13.48* | *-13.30* | *-13.16* | *-13.12* | *-12.98* |
| *257* | *4CO_2_(g) + NH_4_^+^ --> 4CO(g) + NO_3_^-^ + 2H^+^ + H_2_O [8]* | *CO2* | *NH4+* | *-17670* | *390* | *-14.80* | *-14.68* | *-14.58* | *-14.55* | *-14.45* |
| *258* | *4CO(g) + NO_3_^-^ + 2H^+^ + H_2_O --> 4CO_2_(g) + NH_4_^+^ [8]* | *CO2* | *NO2-* | *-18640* | *140* | *-17.61* | *-17.57* | *-17.53* | *-17.52* | *-17.48* |
| **259** | **Fe^+2^ + CO_2_(g) + 2H_2_S(aq) --> CO(g) + PYRITE + 2H^+^ + H_2_O [2]** | **CO2** | **H2S** | **-8860** | **1285** | **0.58** | **1.01** | **1.33** | **1.41** | **1.75** |
| *260* | *CO_2_(g) + H_2_S(aq) --> CO(g) + SULFUR + H_2_O [2]* | *CO2* | *H2S* | *-6500* | *150* | *-5.40* | *-5.35* | *-5.31* | *-5.30* | *-5.26* |
| *261* | *4CO_2_(g) + H_2_S(aq) --> 4CO(g) + SO_4_^-2^ + 2H^+^ [8]* | *CO2* | *H2S* | *-5960* | *590* | *-1.62* | *-1.43* | *-1.28* | *-1.24* | *-1.09* |
| *262* | *PYRITE + CO_2_(g) + 2H^+^ --> CO(g) + 2SULFUR + Fe^+2^ + H_2_O [2]* | *CO2* | *Pyrite* | *-3880* | *-1100* | *-11.97* | *-12.33* | *-12.61* | *-12.67* | *-12.96* |
| *263* | *PYRITE + 7CO_2_(g) + H_2_O --> 7CO(g) + 2SO_4_^-2^ + 2H^+^ + Fe^+2^ [14]* | *CO2* | *Pyrite* | *-5390* | *420* | *-2.30* | *-2.17* | *-2.06* | *-2.03* | *-1.92* |
| **264** | **SULFUR + 3CO_2_(g) + H_2_O --> 3CO(g) + SO_4_^-2^ + 2H^+^ [6]** | **CO2** | **S** | **-5700** | **700** | **-0.56** | **-0.33** | **-0.15** | **-0.10** | **0.08** |
| **265** | **CO_2_(g) + 3Fe^+2^ + 3H_2_O --> MAGNETITE + CO(g) + 6H^+^ [2]** | **CO2** | **Fe+2** | **-27930** | **3600** | **-1.47** | **-0.29** | **0.63** | **0.85** | **1.80** |
| **266** | **CO_2_(g) + 2Fe^+2^ + 2H_2_O --> HEMATITE + CO(g) + 4H^+^ [2]** | **CO2** | **Fe+2** | **-19220** | **2430** | **-1.36** | **-0.56** | **0.06** | **0.21** | **0.84** |
| **267** | **CO_2_(g) + 2Fe^+2^ + 3H_2_O --> 2GOETHITE + CO(g) + 4H^+^ [2]** | **CO2** | **Fe+2** | **-19210** | **2440** | **-1.28** | **-0.48** | **0.15** | **0.30** | **0.94** |
| *268* | *2MAGNETITE + CO_2_(g) --> 3HEMATITE + CO(g) [2]* | *CO2* | *Magnetite* | *-1880* | *150* | *-0.78* | *-0.73* | *-0.69* | *-0.68* | *-0.64* |
| *269* | *2MAGNETITE + CO_2_(g) + 3H_2_O --> 6GOETHITE + CO(g) [2]* | *CO2* | *Magnetite* | *-1840* | *180* | *-0.52* | *-0.46* | *-0.41* | *-0.40* | *-0.35* |
| *270* | *CO_2_(g) --> CO(g) + (1/2)O_2_(aq) [2]* | *CO2* | *N/A* | *-25960* | *260* | *-24.05* | *-23.96* | *-23.90* | *-23.88* | *-23.81* |
| *271* | *3CO_2_(g) + CH_4_(g) --> 4CO(g) + 2H_2_O [8]* | *CO2* | *CH4* | *-3120* | *170* | *-1.87* | *-1.81* | *-1.77* | *-1.76* | *-1.72* |
| 272 | CO_2_(g) + 4H_2_(g) --> CH_4(_g) + 2H_2_O [8] | CO2 | H2 | 1040 | 10 | 1.11 | 1.12 | 1.12 | 1.12 | 1.12 |
| *273* | *3CO_2_(g) + 4NH_4_^+^ + 2H_2_O --> 3CH_4_(g) + 4NO_2_^-^ + 8H^+^ [24]* | *CO2* | *NH4+* | *-14880* | *365* | *-12.20* | *-12.08* | *-11.98* | *-11.96* | *-11.87* |
| *274* | *CO_2_(g) + NH_4_^+^ + H_2_O --> CH_4_(g) + NO_3_^-^ + 2H^+^ [8]* | *CO2* | *NH4+* | *-15180* | *310* | *-12.90* | *-12.80* | *-12.72* | *-12.70* | *-12.62* |
| *275* | *CO_2_(g) + 4NO_2_^-^ + H_2_O --> CH_4_(g) + 4NO_3_^-^ [8]* | *CO2* | *NO2-* | *-16480* | *180* | *-15.16* | *-15.10* | *-15.05* | *-15.04* | *-14.99* |
| 276 | CO_2_(g) + 4Fe^+2^ + 8H_2_S(aq) --> 4PYRITE + CH_4_(g) + 8H^+^ + 2H_2_O [8] | CO2 | H2S | -6500 | 1180 | 2.17 | 2.56 | 2.86 | 2.93 | 3.24 |
| *277* | *CO_2_(g) + 4H_2_S(aq) --> CH_4_(g) + 4SULFUR + 2H_2_O [8]* | *CO2* | *H2S* | *-4140* | *40* | *-3.85* | *-3.83* | *-3.82* | *-3.82* | *-3.81* |
| **278** | **CO_2_(g) + H_2_S(aq) + 2H_2_O --> CH_4_(g) + SO_4_^-2^ + 2H^+^ [8]** | **CO2** | **H2S** | **-3580** | **450** | **-0.27** | **-0.12** | **-0.01** | **0.02** | **0.14** |
| *279* | *4PYRITE + CO_2_(g) + 8H^+^ --> CH_4_(g) + 8SULFUR + 4Fe^+2^ 2H_2_O [8]* | *CO2* | *Pyrite* | *-1670* | *-1150* | *-10.12* | *-10.50* | *-10.79* | *-10.86* | *-11.17* |
| *280* | *4PYRITE + 7CO_2_(g) + 18H_2_O --> 7CH_4_(g) + 8SO_4_^-2^ + 8H^+^ + 4Fe^+2^ [56]* | *CO2* | *Pyrite* | *-3120* | *330* | *-0.69* | *-0.59* | *-0.50* | *-0.48* | *-0.40* |
| 281 | 4SULFUR + 3CO_2_(g) + 10H_2_O --> 3CH_4_(g) + 4SO_4_^-2^ + 8H^+^ [24] | CO2 | S | -3390 | 590 | 0.95 | 1.14 | 1.29 | 1.33 | 1.48 |
| **282** | **CO_2_(g) + 12Fe^+2^ + 14H_2_O --> 4MAGNETITE + CH_4_(g) + 24H^+^ [8]** | **CO2** | **Fe+2** | **-25400** | **3370** | **-0.63** | **0.47** | **1.33** | **1.54** | **2.43** |
| **283** | **CO_2_(g) + 8Fe^+2^ + 10H_2_O --> 4HEMATITE + CH_4_(g) + 16H^+^ [8]** | **CO2** | **Fe+2** | **-16770** | **2250** | **-0.23** | **0.51** | **1.08** | **1.22** | **1.81** |
| **284** | **CO_2_(g) + 8Fe^+2^ + 14H_2_O --> 8GOETHITE + CH_4_(g) + 16H^+^ [8]** | **CO2** | **Fe+2** | **-16770** | **2260** | **-0.16** | **0.58** | **1.16** | **1.30** | **1.89** |
| 285 | 8MAGNETITE + CO_2_(g) + 2H_2_O --> 12HEMATITE + CH_4_(g) [8] | CO2 | Magnetite | 520 | 10 | 0.59 | 0.60 | 0.60 | 0.60 | 0.60 |
| 286 | 8MAGNETITE + CO_2_(g) + 14H_2_O --> 24GOETHITE + CH_4_(g) [8] | CO2 | Magnetite | 580 | 40 | 0.87 | 0.89 | 0.90 | 0.90 | 0.91 |
| *287* | *CO_2_(g) + 2H_2_O --> CH_4_(g) + 2O_2_(aq) [8]* | *CO2* | *N/A* | *-23000* | *40* | *-22.71* | *-22.69* | *-22.68* | *-22.68* | *-22.67* |
