# Supplementary material for: Coordinating Environmental Genomics and Geochemistry Reveals Metabolic Transitions in a Hot Spring Ecosystem
Source: PLoS One. 2012 Jun 4;7(6):e38108. doi: 10.1371/journal.pone.0038108 (PMC3367023; doi:10.1371/journal.pone.0038108)
Supplement: Table S4 — Reactions for each site ranked in order from highest to lowest Affinity values. Ranking number is given in far left column. Reaction numbers correspond to those in Table S3. (DOC) [file pone.0038108.s007.doc]

**Supplemental Table 4.** Reactions for each site ranked in order from highest to lowest Affinity values. Ranking number is given in far left column. Reaction numbers correspond to those in Supplemental Table 3.

| **Ranking** | **Site 1 rxn #** | **Affinity site 1 [kcal/mol e-]** | **Site 2 rxn #** | **Affinity site 2 [kcal/mol e-]** | **Site 3 rxn #** | **Affinity site 3 [kcal/mol e-]** | **Site 4 rxn #** | **Affinity site 4 [kcal/mol e-]** | **Site 5 rxn #** | **Affinity site 5 [kcal/mol e-]** |
| --- | --- | --- | --- | --- | --- | --- | --- | --- | --- | --- |
| 1 | 5 | 24.74 | 5 | 25.06 | 5 | 25.31 | 5 | 25.38 | 12 | 25.95 |
| 2 | 19 | 24.05 | 19 | 23.96 | 12 | 24.74 | 12 | 24.97 | 5 | 25.63 |
| 3 | 10 | 23.74 | 10 | 23.93 | 13 | 24.42 | 13 | 24.58 | 13 | 25.23 |
| 4 | 1 | 23.67 | 12 | 23.79 | 11 | 24.32 | 11 | 24.48 | 11 | 25.13 |
| 5 | 15 | 23.52 | 13 | 23.79 | 10 | 24.08 | 10 | 24.12 | 10 | 24.27 |
| 6 | 14 | 23.33 | 11 | 23.69 | 19 | 23.90 | 19 | 23.88 | 19 | 23.81 |
| 7 | 13 | 22.97 | 1 | 23.62 | 1 | 23.58 | 1 | 23.57 | 1 | 23.53 |
| 8 | 18 | 22.95 | 15 | 23.51 | 15 | 23.50 | 15 | 23.50 | 15 | 23.49 |
| 9 | 11 | 22.87 | 14 | 23.32 | 14 | 23.32 | 14 | 23.32 | 14 | 23.32 |
| 10 | 17 | 22.71 | 18 | 22.93 | 18 | 22.91 | 18 | 22.90 | 18 | 22.88 |
| 11 | 12 | 22.57 | 17 | 22.69 | 17 | 22.68 | 17 | 22.68 | 7 | 22.69 |
| 12 | 7 | 22.35 | 7 | 22.47 | 7 | 22.56 | 7 | 22.59 | 17 | 22.67 |
| 13 | 16 | 22.18 | 9 | 22.29 | 9 | 22.38 | 9 | 22.40 | 9 | 22.49 |
| 14 | 9 | 22.18 | 16 | 22.19 | 16 | 22.20 | 16 | 22.20 | 16 | 22.21 |
| 15 | 6 | 18.24 | 6 | 18.15 | 6 | 18.09 | 6 | 18.07 | 28 | 18.60 |
| 16 | 35 | 17.61 | 35 | 17.57 | 35 | 17.53 | 28 | 17.63 | 6 | 18.00 |
| 17 | 21 | 16.86 | 21 | 17.12 | 28 | 17.40 | 35 | 17.52 | 29 | 17.73 |
| 18 | 26 | 16.19 | 28 | 16.46 | 21 | 17.32 | 21 | 17.37 | 27 | 17.66 |
| 19 | 31 | 15.95 | 26 | 16.39 | 29 | 16.93 | 29 | 17.09 | 21 | 17.57 |
| 20 | 30 | 15.79 | 29 | 16.30 | 27 | 16.86 | 27 | 17.02 | 35 | 17.48 |
| 21 | 20 | 15.68 | 27 | 16.23 | 26 | 16.55 | 26 | 16.59 | 26 | 16.75 |
| 22 | 29 | 15.49 | 31 | 15.95 | 31 | 15.95 | 31 | 15.95 | 46 | 16.26 |
| 23 | 27 | 15.42 | 30 | 15.79 | 30 | 15.80 | 30 | 15.80 | 31 | 15.95 |
| 24 | 32 | 15.38 | 20 | 15.62 | 20 | 15.57 | 20 | 15.56 | 30 | 15.81 |
| 25 | 28 | 15.25 | 32 | 15.39 | 32 | 15.40 | 32 | 15.40 | 20 | 15.51 |
| 26 | 33 | 15.16 | 33 | 15.10 | 39 | 15.16 | 46 | 15.36 | 32 | 15.41 |
| 27 | 23 | 14.84 | 39 | 15.02 | 46 | 15.15 | 39 | 15.20 | 47 | 15.36 |
| 28 | 39 | 14.83 | 23 | 14.97 | 23 | 15.07 | 23 | 15.10 | 39 | 15.35 |
| 29 | 53 | 14.80 | 25 | 14.71 | 33 | 15.05 | 33 | 15.04 | 45 | 15.30 |
| 30 | 25 | 14.60 | 53 | 14.68 | 25 | 14.80 | 25 | 14.82 | 23 | 15.20 |
| 31 | 34 | 14.19 | 56 | 14.32 | 47 | 14.65 | 47 | 14.79 | 63 | 15.01 |
| 32 | 56 | 14.15 | 46 | 14.28 | 45 | 14.59 | 45 | 14.73 | 33 | 14.99 |
| 33 | 44 | 14.00 | 47 | 14.10 | 53 | 14.58 | 53 | 14.55 | 25 | 14.91 |
| 34 | 38 | 13.73 | 44 | 14.09 | 56 | 14.45 | 56 | 14.48 | 56 | 14.61 |
| 35 | 49 | 13.69 | 45 | 14.04 | 44 | 14.17 | 44 | 14.19 | 53 | 14.45 |
| 36 | 48 | 13.52 | 34 | 13.74 | 63 | 13.95 | 63 | 14.15 | 44 | 14.27 |
| 37 | 70 | 13.48 | 38 | 13.64 | 38 | 13.58 | 64 | 13.66 | 78 | 14.26 |
| 38 | 55 | 13.39 | 49 | 13.59 | 64 | 13.53 | 62 | 13.57 | 64 | 14.19 |
| 39 | 47 | 13.38 | 48 | 13.42 | 49 | 13.51 | 38 | 13.56 | 62 | 14.11 |
| 40 | 45 | 13.32 | 55 | 13.31 | 62 | 13.45 | 49 | 13.49 | 88 | 13.91 |
| 41 | 61 | 13.17 | 70 | 13.30 | 34 | 13.38 | 88 | 13.35 | 79 | 13.51 |
| 42 | 46 | 13.16 | 61 | 13.22 | 48 | 13.34 | 48 | 13.32 | 38 | 13.49 |
| 43 | 66 | 12.96 | 63 | 13.12 | 61 | 13.27 | 34 | 13.30 | 77 | 13.42 |
| 44 | 52 | 12.94 | 64 | 13.02 | 55 | 13.25 | 61 | 13.28 | 49 | 13.41 |
| 45 | 51 | 12.90 | 62 | 12.93 | 88 | 13.22 | 55 | 13.23 | 61 | 13.32 |
| 46 | 65 | 12.73 | 66 | 12.82 | 70 | 13.16 | 70 | 13.12 | 48 | 13.24 |
| 47 | 8 | 12.68 | 51 | 12.80 | 51 | 12.72 | 78 | 12.99 | 55 | 13.17 |
| 48 | 50 | 12.58 | 52 | 12.75 | 66 | 12.71 | 51 | 12.70 | 70 | 12.98 |
| 49 | 41 | 12.56 | 88 | 12.67 | 78 | 12.68 | 66 | 12.68 | 85 | 12.96 |
| 50 | 43 | 12.39 | 65 | 12.60 | 52 | 12.61 | 85 | 12.67 | 34 | 12.93 |
| 51 | 64 | 12.35 | 41 | 12.58 | 85 | 12.61 | 41 | 12.61 | 87 | 12.66 |
| 52 | 62 | 12.27 | 50 | 12.51 | 41 | 12.60 | 52 | 12.58 | 41 | 12.63 |
| 53 | 68 | 12.20 | 43 | 12.41 | 65 | 12.49 | 79 | 12.55 | 51 | 12.62 |
| 54 | 63 | 12.06 | 85 | 12.33 | 50 | 12.45 | 65 | 12.46 | 66 | 12.57 |
| 55 | 69 | 12.01 | 8 | 12.28 | 43 | 12.42 | 77 | 12.46 | 52 | 12.43 |
| 56 | 88 | 11.98 | 68 | 12.08 | 79 | 12.33 | 50 | 12.44 | 43 | 12.43 |
| 57 | 85 | 11.97 | 58 | 11.81 | 77 | 12.24 | 43 | 12.42 | 50 | 12.38 |
| 58 | 58 | 11.82 | 69 | 11.75 | 87 | 12.08 | 87 | 12.19 | 73 | 12.36 |
| 59 | 67 | 11.52 | 73 | 11.66 | 68 | 11.98 | 73 | 12.04 | 65 | 12.35 |
| 60 | 60 | 11.51 | 87 | 11.62 | 8 | 11.97 | 68 | 11.96 | 84 | 12.18 |
| 61 | 73 | 11.27 | 60 | 11.47 | 73 | 11.97 | 8 | 11.90 | 81 | 12.17 |
| 62 | 81 | 11.05 | 81 | 11.45 | 58 | 11.80 | 81 | 11.84 | 68 | 11.87 |
| 63 | 87 | 11.04 | 78 | 11.44 | 81 | 11.77 | 58 | 11.80 | 80 | 11.83 |
| 64 | 84 | 10.80 | 67 | 11.42 | 84 | 11.69 | 84 | 11.78 | 58 | 11.79 |
| 65 | 80 | 10.73 | 79 | 11.39 | 69 | 11.54 | 80 | 11.51 | 8 | 11.58 |
| 66 | 22 | 10.61 | 77 | 11.30 | 60 | 11.45 | 69 | 11.49 | 60 | 11.41 |
| 67 | 2 | 10.57 | 84 | 11.30 | 80 | 11.44 | 60 | 11.44 | 69 | 11.28 |
| 68 | 79 | 10.20 | 80 | 11.13 | 67 | 11.33 | 67 | 11.31 | 67 | 11.23 |
| 69 | 83 | 10.12 | 2 | 10.71 | 2 | 10.82 | 83 | 10.86 | 83 | 11.17 |
| 70 | 77 | 10.11 | 22 | 10.51 | 83 | 10.79 | 2 | 10.85 | 2 | 10.96 |
| 71 | 78 | 9.84 | 83 | 10.50 | 82 | 10.56 | 82 | 10.63 | 82 | 10.96 |
| 72 | 82 | 9.84 | 82 | 10.24 | 22 | 10.43 | 22 | 10.41 | 22 | 10.33 |
| 73 | 3 | 9.79 | 3 | 9.90 | 3 | 9.98 | 3 | 10.00 | 3 | 10.08 |
| 74 | 40 | 8.44 | 40 | 8.26 | 40 | 8.12 | 40 | 8.08 | 40 | 7.94 |
| 75 | 57 | 7.91 | 57 | 7.71 | 57 | 7.55 | 57 | 7.51 | 4 | 7.44 |
| 76 | 4 | 7.47 | 4 | 7.46 | 4 | 7.45 | 4 | 7.45 | 57 | 7.35 |
| 77 | 103 | 5.44 | 103 | 5.72 | 94 | 6.05 | 94 | 6.28 | 94 | 7.28 |
| 78 | 101 | 5.40 | 97 | 5.36 | 103 | 5.93 | 103 | 5.99 | 95 | 6.61 |
| 79 | 97 | 5.28 | 101 | 5.35 | 95 | 5.77 | 95 | 5.93 | 93 | 6.53 |
| 80 | 96 | 5.03 | 96 | 5.11 | 93 | 5.69 | 93 | 5.85 | 103 | 6.21 |
| 81 | 24 | 5.03 | 95 | 5.11 | 97 | 5.42 | 97 | 5.44 | 97 | 5.50 |
| 82 | 89 | 5.01 | 94 | 5.08 | 101 | 5.31 | 101 | 5.30 | 101 | 5.26 |
| 83 | 100 | 4.39 | 93 | 5.03 | 96 | 5.17 | 96 | 5.19 | 96 | 5.25 |
| 84 | 95 | 4.26 | 89 | 5.01 | 89 | 5.01 | 89 | 5.01 | 89 | 5.01 |
| 85 | 93 | 4.18 | 24 | 4.63 | 100 | 4.52 | 100 | 4.54 | 100 | 4.60 |
| 86 | 99 | 3.85 | 100 | 4.47 | 24 | 4.32 | 24 | 4.25 | 24 | 3.93 |
| 87 | 94 | 3.83 | 99 | 3.83 | 99 | 3.82 | 99 | 3.82 | 248 | 3.81 |
| 88 | 98 | 3.54 | 98 | 3.55 | 98 | 3.56 | 98 | 3.56 | 99 | 3.81 |
| 89 | 37 | 2.88 | 37 | 3.00 | 242 | 3.18 | 242 | 3.25 | 98 | 3.57 |
| 90 | 42 | 2.75 | 242 | 2.90 | 37 | 3.10 | 37 | 3.13 | 242 | 3.55 |
| 91 | 242 | 2.53 | 148 | 2.66 | 276 | 2.86 | 276 | 2.93 | 138 | 3.37 |
| 92 | 148 | 2.47 | 276 | 2.56 | 148 | 2.81 | 248 | 2.90 | 276 | 3.24 |
| 93 | 146 | 2.30 | 42 | 2.25 | 248 | 2.69 | 148 | 2.85 | 37 | 3.23 |
| 94 | 276 | 2.17 | 146 | 2.17 | 138 | 2.29 | 138 | 2.49 | 153 | 3.03 |
| 95 | 163 | 2.14 | 134 | 1.98 | 249 | 2.12 | 249 | 2.26 | 148 | 3.00 |
| 96 | 167 | 2.12 | 254 | 1.81 | 250 | 2.12 | 250 | 2.26 | 249 | 2.86 |
| 97 | 134 | 2.06 | 248 | 1.80 | 146 | 2.06 | 153 | 2.17 | 250 | 2.86 |
| 98 | 59 | 2.05 | 247 | 1.63 | 140 | 2.01 | 140 | 2.14 | 140 | 2.69 |
| 99 | 254 | 1.87 | 249 | 1.53 | 153 | 1.97 | 139 | 2.06 | 139 | 2.61 |
| 100 | 182 | 1.85 | 250 | 1.53 | 139 | 1.93 | 146 | 2.03 | 282 | 2.43 |
| 101 | 212 | 1.73 | 59 | 1.51 | 134 | 1.92 | 134 | 1.90 | 155 | 2.27 |
| 102 | 178 | 1.70 | 140 | 1.47 | 42 | 1.86 | 247 | 1.81 | 154 | 2.19 |
| 103 | 161 | 1.62 | 142 | 1.45 | 247 | 1.77 | 42 | 1.77 | 247 | 1.95 |
| 104 | 142 | 1.55 | 138 | 1.44 | 254 | 1.77 | 254 | 1.76 | 146 | 1.92 |
| 105 | 208 | 1.54 | 161 | 1.43 | 155 | 1.61 | 155 | 1.74 | 284 | 1.89 |
| 106 | 149 | 1.52 | 149 | 1.39 | 154 | 1.53 | 154 | 1.66 | 134 | 1.84 |
| 107 | 176 | 1.47 | 139 | 1.39 | 142 | 1.37 | 282 | 1.54 | 283 | 1.81 |
| 108 | 247 | 1.45 | 182 | 1.35 | 282 | 1.33 | 259 | 1.41 | 265 | 1.80 |
| 109 | 191 | 1.35 | 252 | 1.32 | 259 | 1.33 | 142 | 1.35 | 259 | 1.75 |
| 110 | 252 | 1.32 | 238 | 1.26 | 252 | 1.32 | 281 | 1.33 | 254 | 1.72 |
| 111 | 141 | 1.30 | 212 | 1.23 | 281 | 1.29 | 252 | 1.32 | 123 | 1.60 |
| 112 | 238 | 1.29 | 167 | 1.22 | 149 | 1.29 | 284 | 1.30 | 281 | 1.48 |
| 113 | 221 | 1.28 | 141 | 1.20 | 161 | 1.28 | 149 | 1.26 | 42 | 1.37 |
| 114 | 172 | 1.23 | 153 | 1.14 | 238 | 1.24 | 161 | 1.24 | 252 | 1.33 |
| 115 | 157 | 1.19 | 281 | 1.14 | 284 | 1.16 | 238 | 1.23 | 142 | 1.27 |
| 116 | 272 | 1.11 | 272 | 1.12 | 141 | 1.12 | 283 | 1.22 | 238 | 1.21 |
| 117 | 251 | 1.02 | 155 | 1.09 | 272 | 1.12 | 272 | 1.12 | 149 | 1.16 |
| 118 | 156 | 1.00 | 157 | 1.06 | 59 | 1.10 | 141 | 1.10 | 272 | 1.12 |
| 119 | 281 | 0.95 | 163 | 1.05 | 283 | 1.08 | 251 | 1.00 | 161 | 1.09 |
| 120 | 187 | 0.92 | 154 | 1.01 | 251 | 1.00 | 59 | 1.00 | 141 | 1.02 |
| 121 | 197 | 0.88 | 251 | 1.01 | 182 | 0.96 | 157 | 0.93 | 251 | 0.99 |
| 122 | 286 | 0.87 | 259 | 1.01 | 157 | 0.96 | 286 | 0.90 | 202 | 0.96 |
| 123 | 217 | 0.84 | 178 | 0.98 | 286 | 0.90 | 182 | 0.87 | 267 | 0.94 |
| 124 | 145 | 0.83 | 286 | 0.89 | 212 | 0.84 | 265 | 0.85 | 286 | 0.91 |
| 125 | 206 | 0.78 | 156 | 0.87 | 156 | 0.78 | 202 | 0.81 | 125 | 0.85 |
| 126 | 249 | 0.78 | 208 | 0.81 | 202 | 0.77 | 123 | 0.79 | 266 | 0.84 |
| 127 | 250 | 0.78 | 206 | 0.73 | 206 | 0.69 | 156 | 0.75 | 124 | 0.84 |
| 128 | 140 | 0.77 | 197 | 0.71 | 265 | 0.63 | 212 | 0.75 | 157 | 0.83 |
| 129 | 227 | 0.72 | 145 | 0.69 | 123 | 0.60 | 206 | 0.68 | 232 | 0.71 |
| 130 | 144 | 0.69 | 202 | 0.62 | 285 | 0.60 | 285 | 0.60 | 156 | 0.65 |
| 131 | 139 | 0.69 | 285 | 0.60 | 145 | 0.59 | 145 | 0.56 | 206 | 0.64 |
| 132 | 248 | 0.66 | 144 | 0.59 | 197 | 0.57 | 232 | 0.55 | 244 | 0.61 |
| 133 | 174 | 0.63 | 284 | 0.58 | 167 | 0.52 | 197 | 0.54 | 285 | 0.60 |
| 134 | 285 | 0.59 | 191 | 0.56 | 232 | 0.51 | 244 | 0.50 | 59 | 0.57 |
| 135 | 259 | 0.58 | 227 | 0.55 | 144 | 0.50 | 144 | 0.48 | 182 | 0.47 |
| 136 | 131 | 0.56 | 283 | 0.51 | 244 | 0.47 | 193 | 0.43 | 145 | 0.45 |
| 137 | 236 | 0.52 | 221 | 0.48 | 193 | 0.43 | 236 | 0.40 | 193 | 0.42 |
| 138 | 193 | 0.46 | 282 | 0.47 | 227 | 0.42 | 227 | 0.39 | 197 | 0.41 |
| 139 | 160 | 0.43 | 236 | 0.46 | 178 | 0.42 | 125 | 0.36 | 144 | 0.40 |
| 140 | 155 | 0.42 | 193 | 0.45 | 236 | 0.41 | 167 | 0.35 | 109 | 0.36 |
| 141 | 202 | 0.42 | 244 | 0.37 | 208 | 0.25 | 124 | 0.35 | 236 | 0.35 |
| 142 | 138 | 0.35 | 232 | 0.35 | 125 | 0.25 | 267 | 0.30 | 212 | 0.35 |
| 143 | 154 | 0.34 | 131 | 0.33 | 124 | 0.23 | 178 | 0.28 | 227 | 0.26 |
| 144 | 119 | 0.32 | 187 | 0.31 | 163 | 0.20 | 266 | 0.21 | 278 | 0.14 |
| 145 | 159 | 0.27 | 176 | 0.29 | 131 | 0.15 | 223 | 0.12 | 235 | 0.11 |
| 146 | 244 | 0.23 | 160 | 0.26 | 267 | 0.15 | 208 | 0.11 | 223 | 0.10 |
| 147 | 189 | 0.23 | 217 | 0.23 | 160 | 0.13 | 131 | 0.10 | 264 | 0.08 |
| 148 | 143 | 0.19 | 172 | 0.22 | 223 | 0.12 | 160 | 0.10 | 246 | 0.07 |
| 149 | 219 | 0.16 | 119 | 0.14 | 266 | 0.06 | 235 | 0.03 | 160 | -0.03 |
| 150 | 223 | 0.16 | 223 | 0.14 | 143 | 0.02 | 278 | 0.02 | 143 | -0.07 |
| 151 | 232 | 0.15 | 159 | 0.12 | 159 | 0.01 | 143 | 0.00 | 131 | -0.08 |
| 152 | 153 | 0.07 | 143 | 0.09 | 235 | 0.01 | 246 | 0.00 | 205 | -0.11 |
| 153 | 169 | -0.07 | 235 | -0.08 | 119 | 0.01 | 163 | 0.00 | 159 | -0.14 |
| 154 | 127 | -0.15 | 246 | -0.09 | 278 | -0.01 | 159 | -0.02 | 119 | -0.17 |
| 155 | 284 | -0.16 | 278 | -0.12 | 246 | -0.02 | 119 | -0.03 | 255 | -0.23 |
| 156 | 235 | -0.18 | 205 | -0.17 | 191 | -0.06 | 264 | -0.10 | 112 | -0.26 |
| 157 | 246 | -0.19 | 123 | -0.18 | 221 | -0.15 | 205 | -0.14 | 178 | -0.30 |
| 158 | 205 | -0.21 | 125 | -0.23 | 205 | -0.15 | 191 | -0.21 | 269 | -0.35 |
| 159 | 283 | -0.23 | 124 | -0.24 | 264 | -0.15 | 255 | -0.27 | 167 | -0.36 |
| 160 | 158 | -0.23 | 265 | -0.29 | 187 | -0.17 | 187 | -0.28 | 110 | -0.39 |
| 161 | 278 | -0.27 | 255 | -0.32 | 217 | -0.25 | 221 | -0.30 | 280 | -0.40 |
| 162 | 171 | -0.35 | 264 | -0.33 | 255 | -0.28 | 109 | -0.35 | 111 | -0.40 |
| 163 | 190 | -0.36 | 127 | -0.35 | 269 | -0.41 | 217 | -0.36 | 108 | -0.47 |
| 164 | 220 | -0.37 | 158 | -0.37 | 112 | -0.42 | 112 | -0.39 | 208 | -0.47 |
| 165 | 255 | -0.38 | 269 | -0.46 | 158 | -0.47 | 269 | -0.40 | 74 | -0.49 |
| 166 | 184 | -0.41 | 174 | -0.47 | 280 | -0.50 | 280 | -0.48 | 204 | -0.59 |
| 167 | 126 | -0.42 | 267 | -0.48 | 127 | -0.51 | 158 | -0.50 | 158 | -0.61 |
| 168 | 214 | -0.42 | 189 | -0.51 | 109 | -0.52 | 111 | -0.54 | 268 | -0.64 |
| 169 | 175 | -0.46 | 112 | -0.55 | 172 | -0.56 | 127 | -0.55 | 199 | -0.65 |
| 170 | 269 | -0.52 | 266 | -0.56 | 111 | -0.57 | 204 | -0.59 | 127 | -0.71 |
| 171 | 264 | -0.56 | 219 | -0.58 | 204 | -0.59 | 268 | -0.68 | 187 | -0.77 |
| 172 | 204 | -0.58 | 280 | -0.59 | 176 | -0.63 | 199 | -0.75 | 229 | -0.83 |
| 173 | 116 | -0.58 | 204 | -0.59 | 268 | -0.69 | 172 | -0.75 | 217 | -0.85 |
| 174 | 282 | -0.63 | 126 | -0.62 | 126 | -0.77 | 110 | -0.79 | 191 | -0.85 |
| 175 | 186 | -0.66 | 111 | -0.70 | 199 | -0.78 | 126 | -0.81 | 163 | -0.88 |
| 176 | 173 | -0.66 | 268 | -0.73 | 110 | -0.88 | 176 | -0.85 | 234 | -0.91 |
| 177 | 280 | -0.69 | 199 | -0.87 | 234 | -0.90 | 108 | -0.87 | 221 | -0.94 |
| 178 | 112 | -0.72 | 234 | -0.89 | 229 | -0.96 | 234 | -0.90 | 126 | -0.96 |
| 179 | 188 | -0.74 | 116 | -1.01 | 108 | -0.96 | 74 | -0.92 | 201 | -1.02 |
| 180 | 216 | -0.77 | 229 | -1.06 | 74 | -1.02 | 229 | -0.93 | 203 | -1.07 |
| 181 | 218 | -0.78 | 184 | -1.08 | 189 | -1.08 | 203 | -1.08 | 261 | -1.09 |
| 182 | 268 | -0.78 | 203 | -1.09 | 203 | -1.08 | 201 | -1.10 | 231 | -1.22 |
| 183 | 125 | -0.84 | 130 | -1.09 | 201 | -1.12 | 189 | -1.22 | 233 | -1.33 |
| 184 | 130 | -0.84 | 214 | -1.09 | 219 | -1.16 | 261 | -1.24 | 75 | -1.37 |
| 185 | 124 | -0.86 | 129 | -1.14 | 261 | -1.28 | 219 | -1.30 | 129 | -1.48 |
| 186 | 111 | -0.87 | 169 | -1.14 | 130 | -1.28 | 231 | -1.30 | 130 | -1.52 |
| 187 | 234 | -0.87 | 201 | -1.20 | 129 | -1.29 | 233 | -1.32 | 172 | -1.56 |
| 188 | 104 | -0.93 | 109 | -1.22 | 231 | -1.32 | 130 | -1.33 | 271 | -1.72 |
| 189 | 129 | -0.95 | 110 | -1.27 | 233 | -1.32 | 129 | -1.33 | 116 | -1.75 |
| 190 | 199 | -1.00 | 104 | -1.30 | 116 | -1.33 | 116 | -1.41 | 176 | -1.80 |
| 191 | 203 | -1.09 | 233 | -1.32 | 174 | -1.33 | 174 | -1.54 | 189 | -1.81 |
| 192 | 229 | -1.19 | 220 | -1.35 | 104 | -1.58 | 104 | -1.65 | 219 | -1.89 |
| 193 | 123 | -1.19 | 186 | -1.35 | 184 | -1.60 | 184 | -1.73 | 263 | -1.92 |
| 194 | 267 | -1.28 | 108 | -1.35 | 214 | -1.61 | 214 | -1.74 | 104 | -1.95 |
| 195 | 201 | -1.30 | 190 | -1.36 | 271 | -1.77 | 271 | -1.76 | 128 | -1.95 |
| 196 | 233 | -1.32 | 231 | -1.40 | 128 | -1.77 | 75 | -1.77 | 184 | -2.27 |
| 197 | 266 | -1.36 | 261 | -1.43 | 75 | -1.86 | 128 | -1.81 | 214 | -2.27 |
| 198 | 128 | -1.45 | 171 | -1.44 | 186 | -1.89 | 186 | -2.02 | 115 | -2.36 |
| 199 | 265 | -1.47 | 74 | -1.44 | 169 | -1.97 | 263 | -2.03 | 174 | -2.43 |
| 200 | 231 | -1.51 | 216 | -1.47 | 216 | -2.01 | 115 | -2.14 | 186 | -2.57 |
| 201 | 115 | -1.57 | 188 | -1.49 | 263 | -2.06 | 216 | -2.14 | 216 | -2.69 |
| 202 | 261 | -1.62 | 218 | -1.53 | 188 | -2.08 | 169 | -2.17 | 188 | -2.82 |
| 203 | 110 | -1.77 | 128 | -1.63 | 115 | -2.08 | 188 | -2.22 | 218 | -2.86 |
| 204 | 108 | -1.85 | 173 | -1.80 | 220 | -2.10 | 218 | -2.26 | 169 | -3.03 |
| 205 | 271 | -1.87 | 271 | -1.81 | 218 | -2.12 | 220 | -2.29 | 118 | -3.05 |
| 206 | 74 | -1.98 | 115 | -1.86 | 190 | -2.14 | 190 | -2.32 | 220 | -3.06 |
| 207 | 109 | -2.12 | 175 | -1.97 | 171 | -2.29 | 171 | -2.49 | 190 | -3.12 |
| 208 | 114 | -2.17 | 263 | -2.17 | 173 | -2.69 | 118 | -2.89 | 114 | -3.24 |
| 209 | 263 | -2.30 | 75 | -2.25 | 118 | -2.86 | 173 | -2.90 | 72 | -3.32 |
| 210 | 118 | -2.51 | 114 | -2.56 | 114 | -2.86 | 114 | -2.93 | 171 | -3.37 |
| 211 | 113 | -2.53 | 118 | -2.70 | 175 | -3.15 | 72 | -3.22 | 113 | -3.55 |
| 212 | 75 | -2.75 | 113 | -2.90 | 113 | -3.18 | 113 | -3.25 | 243 | -3.57 |
| 213 | 72 | -2.96 | 72 | -3.09 | 72 | -3.19 | 175 | -3.44 | 277 | -3.81 |
| 214 | 243 | -3.54 | 243 | -3.55 | 243 | -3.56 | 243 | -3.56 | 173 | -3.81 |
| 215 | 168 | -3.83 | 277 | -3.83 | 277 | -3.82 | 277 | -3.82 | 76 | -3.93 |
| 216 | 277 | -3.85 | 76 | -4.63 | 76 | -4.32 | 76 | -4.25 | 175 | -4.65 |
| 217 | 183 | -4.18 | 183 | -5.03 | 198 | -5.17 | 198 | -5.19 | 198 | -5.25 |
| 218 | 213 | -4.26 | 168 | -5.08 | 260 | -5.31 | 260 | -5.30 | 260 | -5.26 |
| 219 | 76 | -5.03 | 213 | -5.11 | 228 | -5.42 | 228 | -5.44 | 228 | -5.50 |
| 220 | 198 | -5.03 | 198 | -5.11 | 183 | -5.69 | 183 | -5.85 | 133 | -6.22 |
| 221 | 228 | -5.28 | 260 | -5.35 | 213 | -5.77 | 213 | -5.93 | 183 | -6.53 |
| 222 | 260 | -5.40 | 228 | -5.36 | 133 | -5.94 | 133 | -6.00 | 213 | -6.61 |
| 223 | 133 | -5.45 | 133 | -5.73 | 168 | -6.05 | 168 | -6.28 | 168 | -7.28 |
| 224 | 36 | -7.47 | 36 | -7.46 | 36 | -7.45 | 36 | -7.45 | 90 | -7.35 |
| 225 | 90 | -7.91 | 90 | -7.71 | 90 | -7.55 | 90 | -7.51 | 36 | -7.44 |
| 226 | 91 | -8.44 | 91 | -8.26 | 91 | -8.12 | 91 | -8.08 | 91 | -7.94 |
| 227 | 54 | -9.79 | 54 | -9.90 | 54 | -9.98 | 54 | -10.00 | 54 | -10.08 |
| 228 | 245 | -9.84 | 245 | -10.24 | 92 | -10.43 | 92 | -10.41 | 92 | -10.33 |
| 229 | 170 | -9.84 | 279 | -10.50 | 245 | -10.56 | 245 | -10.63 | 245 | -10.96 |
| 230 | 185 | -10.12 | 92 | -10.51 | 279 | -10.79 | 71 | -10.85 | 71 | -10.96 |
| 231 | 279 | -10.12 | 71 | -10.71 | 71 | -10.82 | 279 | -10.86 | 279 | -11.17 |
| 232 | 215 | -10.20 | 230 | -11.10 | 239 | -11.33 | 239 | -11.31 | 239 | -11.23 |
| 233 | 71 | -10.57 | 200 | -11.13 | 230 | -11.39 | 135 | -11.44 | 135 | -11.41 |
| 234 | 92 | -10.61 | 185 | -11.31 | 200 | -11.44 | 230 | -11.46 | 86 | -11.62 |
| 235 | 230 | -10.72 | 215 | -11.39 | 135 | -11.45 | 200 | -11.51 | 230 | -11.77 |
| 236 | 200 | -10.73 | 239 | -11.42 | 150 | -11.80 | 150 | -11.80 | 150 | -11.79 |
| 237 | 135 | -11.51 | 170 | -11.44 | 273 | -11.98 | 86 | -11.94 | 200 | -11.83 |
| 238 | 239 | -11.52 | 135 | -11.47 | 86 | -12.01 | 273 | -11.96 | 273 | -11.87 |
| 239 | 150 | -11.82 | 150 | -11.81 | 185 | -12.25 | 136 | -12.42 | 194 | -12.35 |
| 240 | 262 | -11.97 | 273 | -12.08 | 215 | -12.33 | 240 | -12.44 | 240 | -12.38 |
| 241 | 164 | -12.06 | 86 | -12.32 | 136 | -12.42 | 194 | -12.46 | 136 | -12.43 |
| 242 | 273 | -12.20 | 262 | -12.33 | 240 | -12.45 | 185 | -12.47 | 224 | -12.57 |
| 243 | 179 | -12.27 | 136 | -12.41 | 194 | -12.49 | 215 | -12.55 | 274 | -12.62 |
| 244 | 209 | -12.35 | 240 | -12.51 | 151 | -12.60 | 151 | -12.61 | 151 | -12.63 |
| 245 | 136 | -12.39 | 151 | -12.58 | 262 | -12.61 | 262 | -12.67 | 262 | -12.96 |
| 246 | 151 | -12.56 | 194 | -12.60 | 170 | -12.68 | 224 | -12.68 | 256 | -12.98 |
| 247 | 240 | -12.58 | 274 | -12.80 | 224 | -12.71 | 274 | -12.70 | 195 | -13.24 |
| 248 | 86 | -12.71 | 224 | -12.82 | 274 | -12.72 | 170 | -12.99 | 120 | -13.32 |
| 249 | 194 | -12.73 | 179 | -12.93 | 256 | -13.16 | 256 | -13.12 | 225 | -13.41 |
| 250 | 274 | -12.90 | 209 | -13.02 | 120 | -13.27 | 120 | -13.28 | 185 | -13.43 |
| 251 | 224 | -12.96 | 164 | -13.12 | 195 | -13.34 | 195 | -13.32 | 215 | -13.51 |
| 252 | 165 | -13.16 | 120 | -13.22 | 179 | -13.45 | 225 | -13.49 | 179 | -14.11 |
| 253 | 120 | -13.17 | 256 | -13.30 | 225 | -13.51 | 179 | -13.57 | 209 | -14.19 |
| 254 | 180 | -13.31 | 195 | -13.42 | 209 | -13.53 | 209 | -13.66 | 170 | -14.26 |
| 255 | 210 | -13.38 | 225 | -13.59 | 164 | -13.95 | 164 | -14.15 | 121 | -14.27 |
| 256 | 256 | -13.48 | 180 | -14.04 | 121 | -14.17 | 121 | -14.19 | 257 | -14.45 |
| 257 | 195 | -13.52 | 121 | -14.09 | 105 | -14.45 | 105 | -14.48 | 105 | -14.61 |
| 258 | 225 | -13.69 | 210 | -14.10 | 257 | -14.58 | 257 | -14.55 | 137 | -14.91 |
| 259 | 121 | -14.00 | 165 | -14.28 | 180 | -14.60 | 180 | -14.74 | 275 | -14.99 |
| 260 | 105 | -14.15 | 105 | -14.32 | 210 | -14.65 | 210 | -14.79 | 164 | -15.01 |
| 261 | 137 | -14.60 | 257 | -14.68 | 137 | -14.80 | 137 | -14.82 | 152 | -15.20 |
| 262 | 257 | -14.80 | 137 | -14.71 | 275 | -15.05 | 275 | -15.04 | 180 | -15.32 |
| 263 | 106 | -14.83 | 152 | -14.97 | 152 | -15.07 | 152 | -15.10 | 106 | -15.35 |
| 264 | 152 | -14.84 | 106 | -15.02 | 165 | -15.15 | 106 | -15.20 | 210 | -15.36 |
| 265 | 275 | -15.16 | 275 | -15.10 | 106 | -15.16 | 165 | -15.36 | 241 | -15.41 |
| 266 | 166 | -15.25 | 241 | -15.39 | 241 | -15.40 | 241 | -15.40 | 196 | -15.81 |
| 267 | 241 | -15.38 | 196 | -15.79 | 196 | -15.80 | 196 | -15.80 | 226 | -15.95 |
| 268 | 181 | -15.47 | 226 | -15.95 | 226 | -15.95 | 226 | -15.95 | 165 | -16.26 |
| 269 | 211 | -15.57 | 181 | -16.29 | 122 | -16.55 | 122 | -16.59 | 122 | -16.75 |
| 270 | 196 | -15.79 | 211 | -16.38 | 181 | -16.93 | 181 | -17.09 | 258 | -17.48 |
| 271 | 226 | -15.95 | 122 | -16.39 | 211 | -17.01 | 211 | -17.17 | 107 | -17.57 |
| 272 | 122 | -16.19 | 166 | -16.46 | 107 | -17.32 | 107 | -17.37 | 181 | -17.75 |
| 273 | 107 | -16.86 | 107 | -17.12 | 166 | -17.40 | 258 | -17.52 | 211 | -17.82 |
| 274 | 258 | -17.61 | 258 | -17.57 | 258 | -17.53 | 166 | -17.63 | 102 | -18.00 |
| 275 | 102 | -18.24 | 102 | -18.15 | 102 | -18.09 | 102 | -18.07 | 166 | -18.60 |
| 276 | 147 | -22.18 | 253 | -22.19 | 253 | -22.20 | 253 | -22.20 | 253 | -22.21 |
| 277 | 253 | -22.18 | 147 | -22.29 | 147 | -22.38 | 147 | -22.40 | 147 | -22.49 |
| 278 | 162 | -22.35 | 162 | -22.47 | 162 | -22.56 | 162 | -22.59 | 287 | -22.67 |
| 279 | 177 | -22.57 | 287 | -22.69 | 287 | -22.68 | 287 | -22.68 | 162 | -22.69 |
| 280 | 287 | -22.71 | 207 | -23.32 | 207 | -23.32 | 207 | -23.32 | 207 | -23.32 |
| 281 | 192 | -22.87 | 237 | -23.51 | 237 | -23.50 | 237 | -23.50 | 237 | -23.49 |
| 282 | 222 | -22.97 | 192 | -23.69 | 270 | -23.90 | 270 | -23.88 | 270 | -23.81 |
| 283 | 207 | -23.33 | 222 | -23.79 | 132 | -24.08 | 132 | -24.12 | 132 | -24.27 |
| 284 | 237 | -23.52 | 177 | -23.79 | 192 | -24.32 | 192 | -24.48 | 192 | -25.13 |
| 285 | 132 | -23.74 | 132 | -23.93 | 222 | -24.42 | 222 | -24.58 | 222 | -25.23 |
| 286 | 270 | -24.05 | 270 | -23.96 | 177 | -24.74 | 177 | -24.97 | 117 | -25.63 |
| 287 | 117 | -24.74 | 117 | -25.06 | 117 | -25.31 | 117 | -25.38 | 177 | -25.95 |
