# Supplementary material for: Coordinating Environmental Genomics and Geochemistry Reveals Metabolic Transitions in a Hot Spring Ecosystem
Source: PLoS One. 2012 Jun 4;7(6):e38108. doi: 10.1371/journal.pone.0038108 (PMC3367023; doi:10.1371/journal.pone.0038108)
Supplement: Table S5 — Counts of functional genes found in consensus genomes (non-normalized to the size of the datasets). (DOC) [file pone.0038108.s008.doc]

**Supplemental Table 5.** Counts of functional genes found in consensus genomes (non-normalized to the size of the datasets).

| **Site #** |  |  | **1** |  |  |  |  | **2** |  |  |  | **3** |  |  |  | **4** |  |  |  |  | **5** |  |  |
| --- | --- | --- | --- | --- | --- | --- | --- | --- | --- | --- | --- | --- | --- | --- | --- | --- | --- | --- | --- | --- | --- | --- | --- |
| **Taxon #** | **Aquificae** | **Crenarchaeota** | **Deinococcus-Thermus** | **Proteobacteria** | **Unassigned** | **Aquificae** | **Crenarchaeota** | **Proteobacteria** | **Firmicutes** | **Unassigned** | **Aquificae** | **Deinococcus-Thermus** | **Unassigned** | **Proteobacteria** | **Firmicutes** | **Chloroflexi** | **Cyanobacteria** | **Unassigned** | **Deinococcus-Thermus** | **Proteobacteria** | **Chloroflexi** | **Cyanobacteria** | **Unassigned** |
| sulfite dehydrogenase [sor]: E.C. 1.8.2.1 |  |  | 1 |  |  |  |  |  |  |  |  | 3 |  |  |  |  |  |  |  |  |  |  |  |
| sulfite oxidase [so]; E.C. 1.8.3.1 |  |  | 1 |  |  |  |  |  |  |  |  | 3 |  |  |  |  |  |  |  |  |  |  |  |
| sulfate adenylyltransferase (ADP) [APAT]: E.C. 2.7.7.5 | 12 |  |  | 1 | 2 | 5 |  | 1 |  | 4 |  |  | 4 | 3 |  | 2 | 2 | 7 |  | 2 | 2 | 1 | 13 |
| 3'(2'), 5'-bisphosphate nucleotidase: E.C. 3.1.3.7 |  |  | 1 |  |  |  |  |  |  | 1 |  | 1 | 1 |  | 1 | 5 | 6 | 2 |  | 2 | 1 | 4 | 1 |
| soxA | 10 |  |  |  |  | 5 | 1 |  |  | 2 |  |  | 1 |  |  |  |  |  |  |  |  |  |  |
| soxB | 13 |  |  |  |  | 7 |  |  |  | 1 | 2 | 5 |  |  |  |  |  |  | 2 |  |  |  |  |
| soxC |  |  | 1 |  |  |  |  |  |  |  |  | 3 |  |  |  |  |  |  | 2 |  |  |  |  |
| soxD |  |  |  |  |  |  |  |  |  |  |  | 2 |  |  |  |  |  |  |  |  |  |  |  |
| soxX | 10 |  |  |  | 1 | 4 |  |  |  | 2 |  |  | 2 |  |  |  |  |  |  |  |  |  |  |
| soxY | 7 |  |  |  |  | 6 |  |  |  |  |  |  |  |  |  |  |  |  |  |  |  |  |  |
| soxZ | 6 |  |  |  |  | 5 |  |  |  |  |  |  |  |  |  |  |  |  |  |  |  |  |  |
| thiosulfate-quinione-oxidoreductase [tqr] |  |  |  |  |  |  |  |  |  |  |  |  |  |  |  |  |  |  | 1 |  |  |  | 1 |
| sqr | 16 |  | 1 |  |  | 4 |  |  |  |  | 1 |  |  |  |  |  |  |  |  |  |  |  |  |
| sulfide dehydrogenase (flavocytochrome) [fcsd] | 18 |  |  |  | 2 | 6 |  |  |  | 4 | 1 | 3 |  |  |  |  |  |  | 2 |  |  |  |  |
| sulfur oxidizing protein [sox]: E.C. 3.1.3.5 | 13 |  |  |  |  | 6 |  |  |  | 2 | 1 | 6 | 1 |  |  |  |  | 1 | 2 |  |  |  |  |
| sulfite reductase sironeme [dsr]: E.C. 1.8.1.2 & 1.8.7.1 |  |  |  |  |  |  |  |  |  |  |  |  | 1 | 1 |  |  | 6 | 2 |  | 1 |  | 3 | 4 |
| sulfite reductase siroheme [dsr]: E.C. 1.8.99.3 |  | 2 |  |  | 1 |  | 5 |  |  |  |  |  |  |  |  |  |  |  |  |  |  |  |  |
| dsr oxidoreductase |  | 3 |  |  | 1 |  | 5 |  |  |  |  |  |  |  |  |  |  |  |  |  |  |  |  |
| sulfite reductase [dsr]: E.C. 1.8.1.2 |  |  |  | 1 | 2 |  |  | 1 |  | 3 |  | 2 | 1 | 2 | 1 | 4 | 6 | 2 |  | 2 | 7 | 2 | 3 |
| phosphoadenosine phosphosulfate reductase [apr]: E.C. 1.8.4.8 |  |  |  |  |  |  | 3 |  |  | 2 |  | 1 |  | 1 |  |  | 1 | 1 |  | 1 | 1 | 1 |  |
| sulfite reductase [dsr]: E.C. 1.8.7.1 |  |  |  | 1 |  |  |  | 1 |  | 1 |  | 2 | 1 | 1 |  |  | 6 | 2 |  | 1 |  | 2 | 3 |
| sulfite reductase [dsr]: E.C. 1.8.99.2 |  | 4 |  |  |  |  | 5 |  |  |  |  |  |  |  |  |  |  |  |  |  |  |  |  |
| 3'-phosphoadenosine 5'-phosphosulfate synthase [apr]: E.C. 2.7.1.25 | 3 |  |  | 1 |  |  | 4 | 1 | 1 | 4 |  |  |  | 1 | 1 | 6 | 9 | 5 |  | 2 | 5 | 4 | 4 |
| 3'-phosphoadenosine 5'-phosphosulfate synthase sat]: E.C. 2.7.7.4 | 20 | 3 | 1 | 3 | 2 | 11 | 3 | 3 | 1 | 4 | 3 | 2 | 6 | 2 | 1 | 14 | 15 | 6 | 1 | 5 | 9 | 6 | 9 |
|  |  |  |  |  |  |  |  |  |  |  |  |  |  |  |  |  |  |  |  |  |  |  |  |
| nitrogenase [nif]: E.C. 1.18.6.1 |  |  |  |  |  |  |  |  |  |  |  |  |  |  |  | 10 | 18 | 9 |  |  | 11 | 5 | 7 |
| ferredoxin-nitrate reductase [nar]: E.C. 1.7.7.2 | 3 | 2 |  | 1 | 2 | 1 | 1 | 5 |  | 5 |  |  | 1 | 5 |  | 1 | 11 | 8 |  | 3 | 2 | 4 | 8 |
| nitrate reductase [nar]: E.C. 1.7.99.4 | 3 | 4 | 2 | 3 | 4 | 1 | 6 | 10 |  | 7 |  | 6 | 1 | 15 |  | 6 | 11 | 13 | 2 | 10 | 4 | 4 | 12 |
| ferredoxin-nitrite reductase [nir]: E.C. 1.7.1.1 | 27 | 3 | 2 | 3 | 4 | 10 | 2 | 8 |  | 8 | 2 | 6 | 3 | 10 |  | 8 | 11 | 10 |  | 7 | 8 | 4 | 11 |
| nitrite reductase [nir]: E.C. 1.7.1.4 | 4 |  |  | 2 |  | 1 |  | 3 |  | 9 |  |  | 4 | 6 |  | 1 | 4 | 1 |  | 4 | 1 | 1 | 1 |
| nitrite reductase [nir]: E.C. 1.7.2.1 |  |  |  |  |  |  |  |  |  |  |  |  |  |  |  |  |  |  |  |  |  |  | 2 |
| formate-dependent nitrite reductase: E.C. 1.7.2.2 |  |  |  |  |  |  |  |  |  |  |  |  |  |  |  |  |  |  |  |  |  |  | 1 |
| ferredoxin-nitrite reductase [nir]: E.C. 1.7.7.1 | 4 |  |  |  |  | 1 |  | 1 |  | 11 |  | 2 | 4 |  |  |  | 4 |  |  |  |  | 1 | 1 |
| nitric-oxide reductase [nor]: E.C. 1.7.99.7 |  |  |  |  |  |  |  |  |  |  |  |  |  |  |  |  |  | 3 |  |  |  |  | 2 |
| norD |  |  |  |  |  |  |  |  |  |  |  |  |  |  |  |  |  | 2 |  |  |  |  | 4 |
| norQ |  |  |  |  |  |  |  |  |  |  |  |  |  |  |  |  |  | 2 |  |  |  |  | 2 |
| nitrous-oxide reductase [nos]: E.C. 1.7.99.6 |  |  |  |  |  |  |  |  |  | 4 |  |  | 3 |  |  |  |  | 1 |  |  |  |  | 2 |
| hydroxylamine reductase: E.C. 1.7.99.1 |  |  |  |  |  |  |  | 1 |  |  |  |  |  | 2 |  |  |  |  |  | 1 |  |  | 1 |
| hydroxylamine oxidase [hao]: E.C. 1.7.3.4 |  |  |  |  |  |  |  |  |  |  |  |  |  |  |  |  |  |  |  |  |  |  | 1 |
|  |  |  |  |  |  |  |  |  |  |  |  |  |  |  |  |  |  |  |  |  |  |  |  |
| Citryl-CoA synthetase small subunit: E.C. 6.2.1.5 | 9 |  | 1 |  | 8 | 6 | 1 | 1 |  | 9 | 2 | 2 | 1 |  |  | 3 |  | 13 | 1 |  | 2 |  | 10 |
| Citryl-CoA synthetase large subunit: E.C. 6.2.1.5 | 13 | 1 |  |  | 6 | 9 | 1 |  |  | 6 |  | 2 | 1 |  |  | 6 |  | 21 | 1 |  | 3 |  | 13 |
| Citryl-CoA lyase: E.C. 2.3.3.1 | 7 |  |  |  | 1 | 3 |  |  |  |  |  |  |  |  |  |  |  |  |  |  |  |  |  |
| pyruvate ferredoxin oxidoreductase, alpha [OorA] |  | 4 |  |  |  |  | 4 |  |  | 1 |  |  |  |  |  |  |  | 1 |  |  |  |  |  |
| pyruvate ferredoxin oxidoreductase, beta [OorB] |  | 6 |  |  |  |  | 5 |  |  | 2 |  |  |  |  |  |  |  |  |  |  |  |  | 1 |
| pyruvate ferredoxin oxidoreductase, gamma [OorC] |  | 4 |  |  |  |  | 3 |  |  |  |  |  |  |  |  |  |  |  |  |  |  |  | 2 |
| pyruvate ferredoxin oxidoreductase,delta [OorD] |  | 2 |  |  |  |  | 3 |  |  | 2 |  |  |  |  |  |  |  |  |  |  |  |  |  |
| Rubisco, large SU: E.C. 3.1.3.77 & 4.1.1.39 |  |  |  |  | 1 |  |  |  |  |  |  |  | 1 |  |  |  | 5 | 2 |  |  | 1 | 2 | 2 |
| CO dehydrogenase (aerobic): E.C. 1.2.99.2 & 1.17.1.4 |  | 6 |  |  | 4 |  | 4 |  |  | 39 |  | 10 | 1 |  |  | 14 |  | 12 |  |  | 12 |  | 11 |
| 3-hydroxypropionate/malyl-CoA cycle |  |  |  |  |  |  |  |  |  |  |  |  |  |  |  |  |  |  |  |  |  |  |  |
| propionyl CoA synthase | 3 | 7 | 3 |  | 23 | 3 | 15 |  |  | 31 | 1 | 1 | 10 | 2 |  | 21 |  | 34 |  | 1 | 20 |  | 39 |
| malonate semialdehyde reductase |  |  |  |  |  |  |  |  |  |  |  |  |  |  |  | 11 |  | 1 |  |  | 11 |  |  |
| 3-hydroxypropionate/4-hydroxybutyrate |  |  |  |  |  |  |  |  |  |  |  |  |  |  |  |  |  |  |  |  |  |  |  |
| 4-hydroxybutyryl-CoA dehydratase: E.C. 1.14.13.3 |  |  | 2 |  | 1 |  |  |  |  | 5 |  | 1 | 5 | 1 |  | 5 |  |  |  | 1 | 3 |  | 1 |
